# Fluorescent Ligands Enable Target Engagement Studies for the Intracellular Allosteric Binding Site of the Chemokine Receptor CXCR2

**DOI:** 10.1021/acs.jmedchem.3c00769

**Published:** 2023-07-18

**Authors:** Max E. Huber, Silas Wurnig, Lara Toy, Corinna Weiler, Nicole Merten, Evi Kostenis, Finn K. Hansen, Matthias Schiedel

**Affiliations:** † Department of Chemistry and Pharmacy, Medicinal Chemistry, Friedrich-Alexander-University Erlangen-Nürnberg, Nikolaus-Fiebiger-Straße 10, 91058 Erlangen, Germany; ‡ Department of Pharmaceutical & Cell Biological Chemistry, Pharmaceutical Institute, University of Bonn, An der Immenburg 4, 53121 Bonn, Germany; § Molecular, Cellular and Pharmacobiology Section, Institute for Pharmaceutical Biology, University of Bonn, Nussallee 6, 53115 Bonn, Germany; ∥ Institute of Medicinal and Pharmaceutical Chemistry, Technische Universität Braunschweig, Beethovenstraße 55, 38106 Braunschweig, Germany

## Abstract

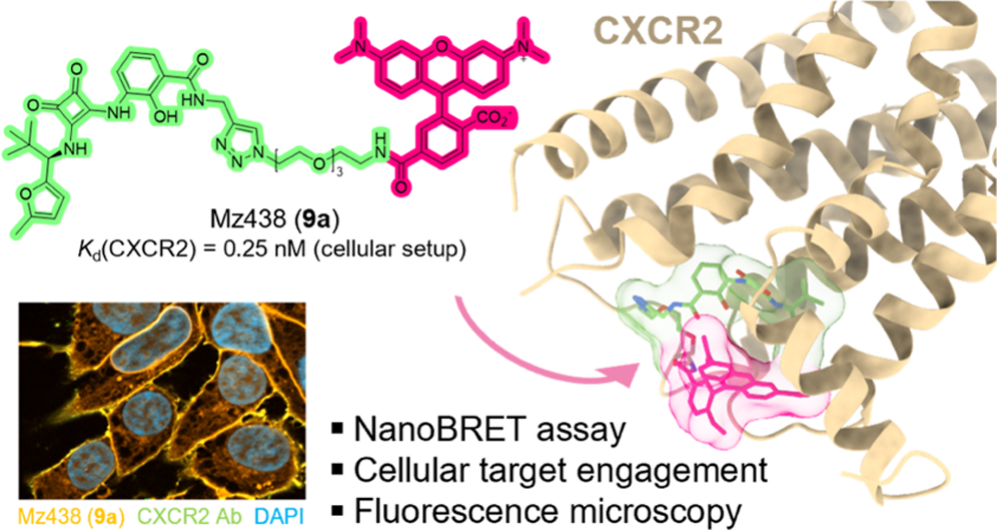

Herein, we report the structure-based development of fluorescent ligands targeting the intracellular allosteric binding site (IABS) of CXC chemokine receptor 2 (CXCR2), a G protein-coupled receptor (GPCR) that has been pursued as a drug target in oncology and inflammation. Starting from the cocrystallized intracellular CXCR2 antagonist 00767013 (**1**), tetramethylrhodamine (TAMRA)-labeled CXCR2 ligands were designed, synthesized, and tested for their suitability as fluorescent reporters to probe binding to the IABS of CXCR2. By means of these studies, we developed Mz438 (**9a**) as a high-affinity and selective fluorescent CXCR2 ligand, enabling cell-free as well as cellular NanoBRET-based binding studies in a nonisotopic and high-throughput manner. Further, we show that **9a** can be used as a tool to visualize intracellular target engagement for CXCR2 via fluorescence microscopy. Thus, our small-molecule-based fluorescent CXCR2 ligand **9a** represents a promising tool for future studies of CXCR2 pharmacology.

## Introduction

**Figure 1:**
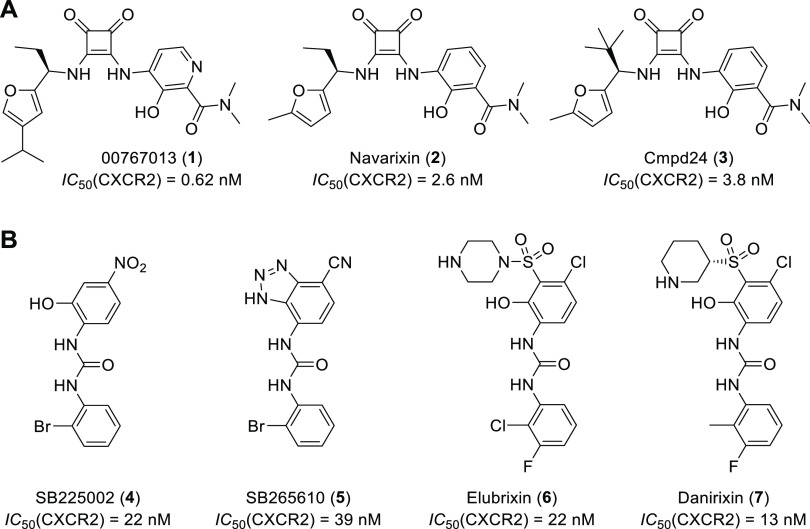
Chemical structures and reported IC_50_ values of known intracellular CXCR2 antagonists **1**–**7**. (A) Squaramide-based CXCR2 antagonists. (B) CXCR2 antagonists based on an *N*,*N*′-diarylurea scaffold. References: 00767013 (**1**),^4^ navarixin (**2**),^14^ cmpd24 (**3**),^14^ SB225002 (**4**),^15^ SB265610 (**5**),^16^ elubrixin (**6**),^17^ and danirixin (**7**).^18^

G protein-coupled receptors (GPCRs) are pharmaceutically highly relevant as they represent the drug targets for approximately one-third of all available medications.^1^ The vast majority of the reported GPCR ligands binds to an orthosteric site, which is located within the helical bundle, facing the extracellular side of the receptor.^2^ Apart from the orthosteric site, an intracellular allosteric binding site (IABS) was recently identified by X-ray cocrystallography for several GPCRs, including the chemokine receptors CCR2,^3^ CXCR2,^4^ CCR7,^5^ and CCR9^6^ as well as for the β-2 adrenergic receptor (β_2_AR).^7^ CXCR2 was cocrystallized with the intracellular small-molecule antagonist 00767013 (**1**, Figure 1). Further, a druggable IABS has been suggested for several other GPCRs.^8^ Ligands targeting this allosteric binding site feature a new dual mechanism of specific GPCR modulation, which is characterized by stabilization of the inactive receptor conformation resulting in a negative cooperativity with the orthosteric agonist and a steric blockage of intracellular transducer (G protein and/or β-arrestin) binding.^3,9,10^ Taking advantage of this new approach of GPCR antagonism is especially attractive for GPCR families, for which the development of orthosteric antagonists only showed very limited therapeutic success, such as the chemokine receptors.^11^

The chemokine receptor CXCR2 is broadly expressed in a range of leukocytes, most notably in polymorphonuclear (PMN) cells, and is implicated in neutrophil trafficking and recruitment.^12^ CXCR2-mediated signaling is associated with the pathogenesis of various diseases, including inflammatory diseases affecting the lungs such as chronic obstructive pulmonary disease (COPD) and asthma bronchiale, other inflammatory diseases like ulcerative colitis and psoriasis, and also cancer, thus making CXCR2 a promising target for pharmaceutical intervention. This has prompted intense efforts in the development of small-molecule inhibitors of CXCR2, which are extensively reviewed elsewhere.^13^ A selection of high-affinity antagonists (**1**–**7**) that are known to bind to the IABS of CXCR2 is shown in Figure 1.

In the late 2000’s, site-directed mutagenesis studies provided first evidence for an intracellular allosteric binding mode of both squaramide- and diarylurea-based CXCR2 antagonists, as mutations to the intracellular face of the receptor were detrimental to antagonist binding.^19,20^ In 2020, a cocrystal structure of CXCR2 and **1** (PDB: 6LFL), a close structural analogue of navarixin (**2**), finally validated the intracellular binding mode of squaramide-based CXCR2 antagonists.^4^**2** even progressed to phase II clinical trials for the treatment of several inflammatory diseases, including COPD, asthma, and psoriasis, thus, highlighting the therapeutic potential of intracellular CXCR2 antagonists.^21^ However, similar to other clinically tested intracellular CXCR2 antagonists like **6** and **7**, all trials with **2** were terminated after phase II due to limited efficacy.^13^ Thus, new approaches to improve the therapeutic efficacy of intracellular CXCR2 antagonism are urgently needed.

With the disclosure of the crystal structure of CXCR2 in complex with **1**,^4^ Liu et al. laid the foundation for the structure-based development of novel CXCR2 antagonists with improved therapeutic efficacies. In addition to detailed structural insights into receptor–ligand interactions gained by the **1**-CXCR2 cocrystal structure,^4^ molecular tools that enable an unambiguous identification and characterization of intracellular CXCR2 ligands are of utmost importance for drug discovery campaigns in this area. With the radioligands [^3^H]navarixin (also referred to as [^3^H]Sch527123) and [^3^H]SB265610, Salchow et al. have therefore reported highly valuable molecular tools that can be utilized to detect binding to the IABS of CXCR2.^19^ However, radioligand binding assays are accompanied by several disadvantages, such as high infrastructure requirements according to radiation protection measures, the production of radioactive waste, and often laborious (heterogeneous) assay protocols, including washing steps, to remove the unbound radioligand prior to the assay readout. The latter is also an important reason why radioligand binding assays are often not well-suited for a continuous readout, the detection of low-affinity binders, and cellular target engagement studies investigating the binding to intracellular binding sites. Recently, we reported the development of fluorescent tracers targeting the IABS of CCR2 and CCR9.^22,23^ These molecular tools were successfully applied for cell-free and cellular binding studies using the nonisotopic NanoBRET technology. Herein, we aimed at developing a fluorescently labeled intracellular CXCR2 ligand to provide a nonisotopic molecular tool that allows to directly study ligand binding to the IABS of CXCR2 both in a cell-free and cellular environment.

## Results

**Figure 2:**
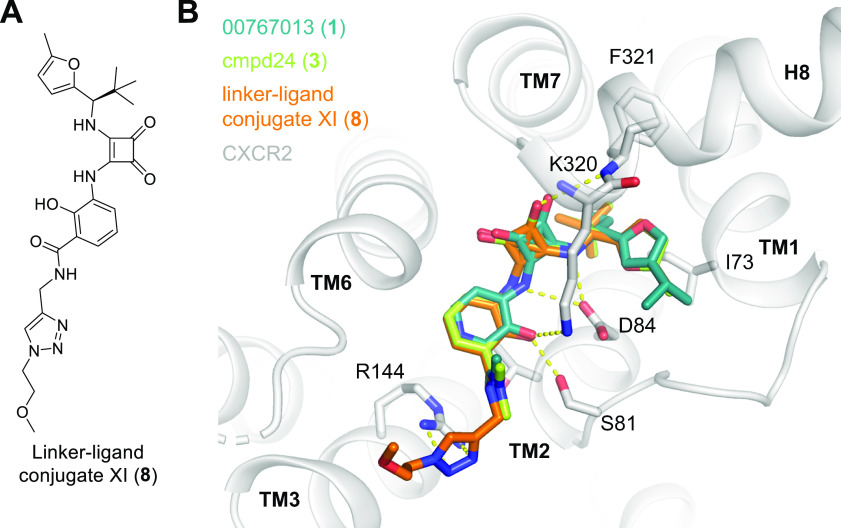
Design of fluorescent ligands targeting the intracellular allosteric binding site of CXCR2. (A) Chemical structure of CXCR2 ligand–linker conjugate XI (**8**) that was identified by molecular docking as a suitable template for the design of fluorescent ligands. (B) Overlay of the reported binding mode of 00767013 (**1**, PDB ID: 6LFL)^4^ with the predicted binding modes for cmpd24 (**3**) and the cmpd24-derived ligand–linker conjugate XI (**8**). H-bond interactions are indicated as dashed yellow lines. Docking scores (AutoDock Vina): **1** = −10.3; **3** = −10.6; and **8** = −10.1.

For the structure-based design of fluorescently labeled intracellular CXCR2 ligands, we used the **1**-CXCR2 cocrystal structure (PDB: 6LFL)^4^ as a starting point. Docking studies with ligand–linker conjugates derived from **1** and also from other reported squaramide-based intracellular CXCR2 antagonists, including **2** and **3**,^14^ indicated that especially the benzamide moiety of **3** will allow the installation of a linker unit while retaining receptor affinity (Figures 2 and S1). Moreover, our docking experiments revealed promising properties for triazole-containing linkers, which enable a straightforward conjugation with a fluorophore by Cu(I)-catalyzed Huisgen cycloaddition.^24−26^ Further, our docking studies predicted that ligand–linker conjugates with a secondary benzamide will bind with similar or even higher affinities compared to tertiary benzamides (Figure S1). Thus, we based the design of our fluorescent CXCR2 ligands on the ligand–linker conjugate XI (**8**, Figures 2 and S1). Finally, the fact that the distal methyl groups at the dimethylbenzamide moiety of **1** were shown to be already solvent-exposed (Figure 2) suggested that a rather short linker of three poly(ethylene glycol) (PEG) units should be sufficient for the design of our fluorescent probes.

**Scheme 1:**
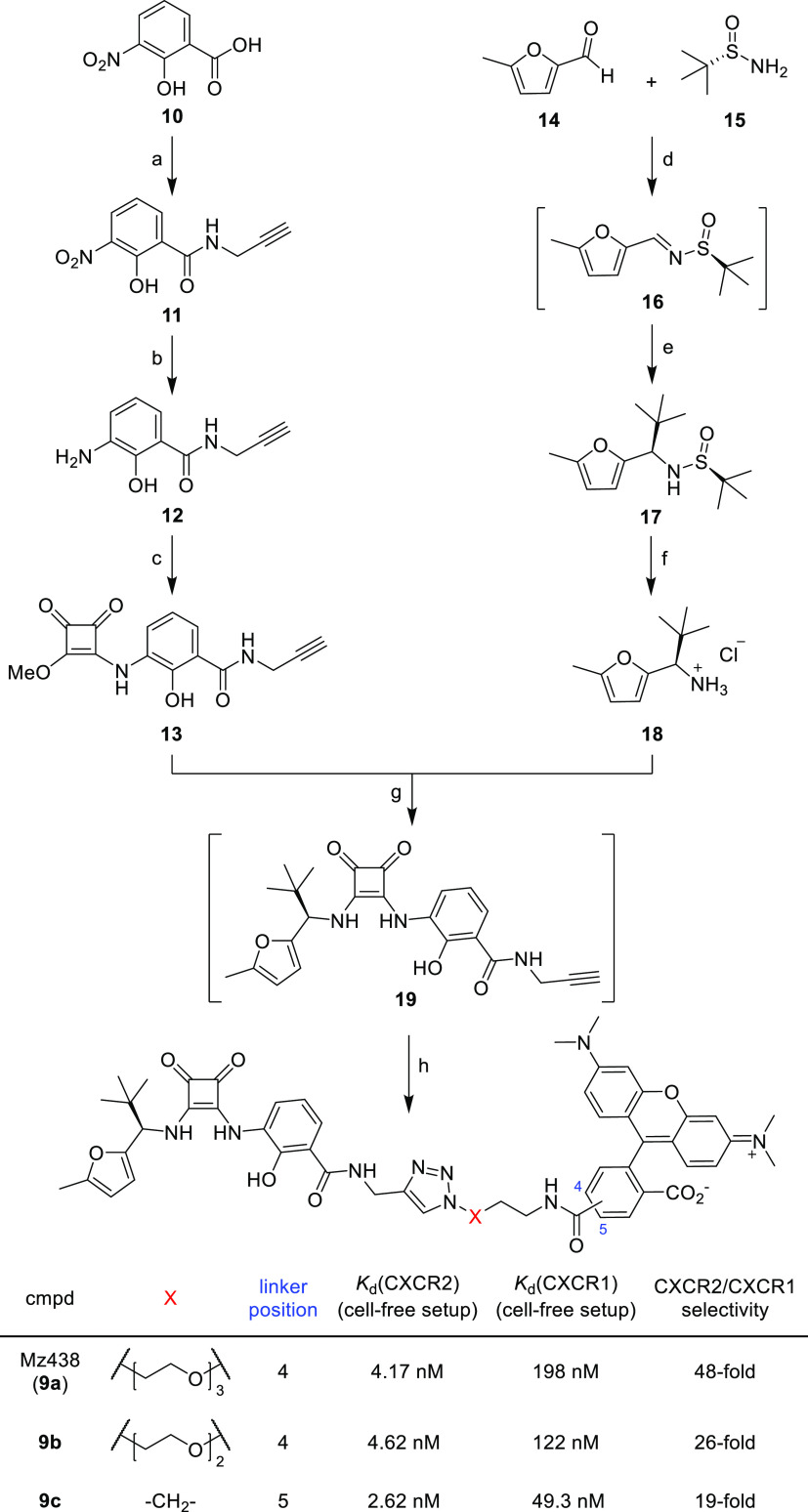
Synthesis of Fluorescent CXCR2 Antagonists **9a**–**c** Reagents and conditions: (a) Propargylamine, PyBroP, *N*,*N*-diisopropylethylamine (DIPEA), CH_2_Cl_2_, rt, overnight, 79% yield. (b) SnCl_2_·2 H_2_O, MeOH, reflux, 3 h, 89% yield. (c) Dimethyl squarate, MeOH, rt, 3 days, 91% yield. (d) Ti(OEt)_4_, CH_2_Cl_2_, rt, overnight. (e) *tert-*Butyl magnesium chloride, tetrahydrofuran (THF), 0 °C to rt, 3 days, 54% yield over two steps. (f) HCl (in Et_2_O), MeOH, rt, 3 h, 46% yield. (g) DIPEA, MeOH, rt, 7 days, 38% yield (h) for **9a**-**b**: azido-functionalized TAMRA analogue, CuSO_4_·5 H_2_O, sodium ascorbate, tris(benzyltriazolylmethyl)-amine (TBTA), water/*tert*-BuOH/*N*,*N*-dimethylformamide (DMF) mixture (1:1:1 (v/v)), rt, 1 h, 30–50% yield; for **9c**: 5-TAMRA-azide, CuSO_4_·5H_2_O, sodium ascorbate, water/*tert*-BuOH/DMF mixture (1:1:1 (v/v)), rt, 1 h, 41% yield.

Following the suggestions from our docking studies, we established a synthesis route toward the squaramide-based fluorescent CXCR2 ligand Mz438 (**9a**, Scheme 1). To provide initial insights into linker length influences on CXCR2 affinity and selectivity, we also synthesized **9b**,**c**.

In general, our synthetic approach refers to previously published protocols for the synthesis of intracellular squaramide-based CXCR2 antagonists.^14,27,28^ In order to enable a late-stage conjugation of the CXCR2-targeted unit with a cell-permeable TAMRA-based fluorophore, which has widely been applied as an acceptor fluorophore for NanoBRET assays,^22,23,29,30^*via* straightforward Cu(I)-catalyzed azide–alkyne cycloaddition (CuAAC),^24−26^ we installed a propargyl handle in the first step of the reaction sequence by means of amide bond formation. To this end, benzoic acid **10** was activated using PyBroP prior to its reaction with propargylamine. The aromatic nitro group of the formed propargyl amide **11** was then reduced to obtain the primary aromatic amine **12**. Coupling of **12** with dimethyl squarate resulted in **13**, which upon treatment with the chiral amine **18** afforded the clickable CXCR2 ligand **19**, which was directly reacted with an azido-functionalized TAMRA analogue, to finally yield the fluorescent CXCR2 probes **9a**–**c**. The optically pure amine fragment **18** was synthesized using a diastereoselective chiral addition route.^31,32^ Condensation of 5-methylfurfuryl aldehyde (**14**) with (*S*)-2-methylpropane-2-sulfinamide (**15**) afforded the imine intermediate **16**, which was directly reacted with *tert*-butyl magnesium chloride to generate **17**. ^1^H NMR, ^13^C NMR, and high-performance liquid chromatography (HPLC) experiments (see the Supporting Information) confirmed that **17** was obtained as a diastereoselective addition product. Subsequent hydrolysis of sulfinamide **17** yielded the enantiopure amine **18**.

**Figure 3:**
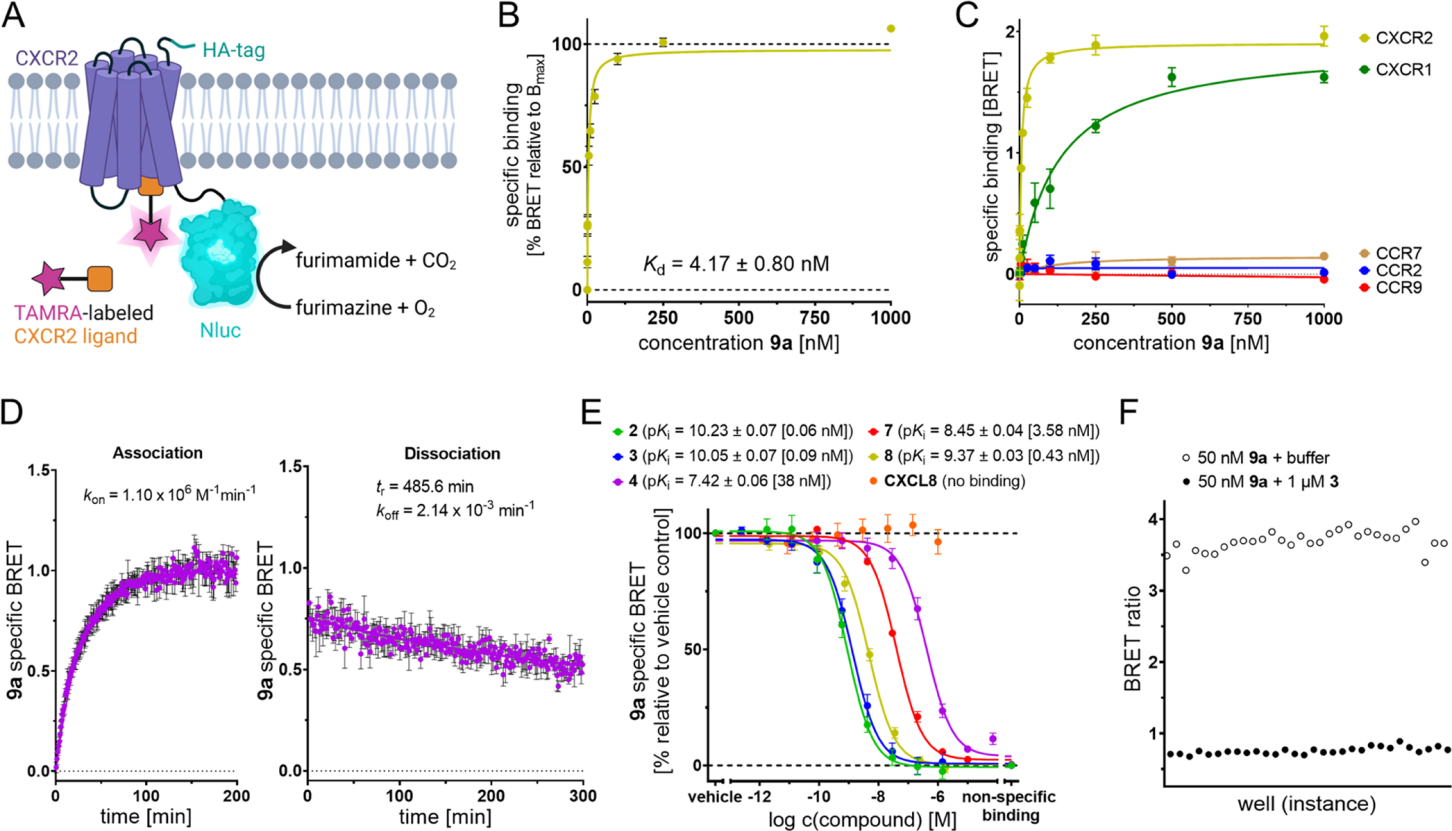
Application of **9a** as a fluorescent tool for cell-free NanoBRET binding studies targeting the IABS of CXCR2. (A) Cartoon representation of the NanoBRET strategy to detect binding to the IABS of CXCR2. (B) Specific saturation binding curve of **9a** in a NanoBRET-based assay using CXCR2_Nluc membranes (mean ± standard error of the mean (SEM), triplicate measurement, *n* = 9). See Figure S2F for a more detailed view on the concentration range from 0 to 100 nM. (C) Comparison of representative specific binding curves of the fluorescent ligand **9a** binding to membrane preparations from HEK293T cells expressing the respective C-terminally Nluc-tagged chemokine receptor (CCR2, CCR7, CCR9, CXCR1, and CXCR2). The experiments were performed in triplicate (*n* ≥ 3). See Figures S5C–E, S4D, and S2E for representative curves for total, specific, and nonspecific binding of **9a**. (D) Kinetic binding studies at room temperature. Representative association and dissociation curves with **9a** (50 nM) using CXCR2_Nluc membranes. Further information on kinetic binding studies is provided in Figure S2G and Table S1A,B. (E) Competition binding curves and detected p*K*_i_ values (mean ± SEM, triplicate measurement, *n* ≥ 3) for the known intracellular allosteric CXCR2 antagonists **2**–**4** and **7**, the ligand–linker conjugate XI (**8**), and the extracellular orthosteric agonist CXCL8, obtained with **9a** (50 nM) and CXCR2_Nluc membranes. *K*_i_ values are given in square brackets. (F) *Z*′-factor determination for the cell-free NanoBRET-based setup using **9a** (50 nM) and CXCR2_Nluc membranes (30-fold determination).

To evaluate the binding affinity of the synthesized fluorescent CXCR2 ligands **9a**–**c**, we developed a NanoBRET-based binding assay (Figures 3A–F and S2A–I). To this end, we fused CXCR2 at its intracellular C-terminus to a small and bright luciferase variant (nanoluciferase, Nluc, Figures 3A and S2A).^33^ Successful surface expression of the 3xHA-CXCR2-Nluc fusion protein (hereafter referred to as CXCR2_Nluc), was confirmed by enzyme-linked immunosorbent assay (ELISA) directed against an N-terminal 3xHA-tag (Figure S2B). In saturation binding experiments using membranes from HEK293T cells transiently expressing CXCR2_Nluc, **9a** showed a low nanomolar affinity with a *K*_d_ value of 4.17 ± 0.80 nM (Figures 3B and S2E,F). For **9b,c**, we detected similar affinities with *K*_d_ values of 4.62 ± 0.87 and 2.62 ± 0.78 nM, respectively (Scheme 1 and Figure S3A–D). This indicates a minor influence of linker lengths on CXCR2 affinity. First, selectivity studies revealed that **9a–c** also bind with sub-micromolar affinities to the closely related CXCR1 (for **9a**: *K*_d_(CXCR1) = 198 ± 29 nM; for **9b**: *K*_d_(CXCR1) = 122 ± 5.6 nM; for **9c**: *K*_d_(CXCR1) = 49.3 ± 0.7 nM; see Figure S4A–F). This was expected, given by the low CXCR2/CXCR1 selectivity of the parent ligand **3**.^14^ However, **9a** still shows a ∼50-fold selectivity for CXCR2 compared to CXCR1. Due to its high CXCR2 selectivity, we selected **9a** for further studies. In a broader selectivity screening, no significant binding of **9a** was detected to other chemokine receptors with a reported druggable IABS, such as CCR2, CCR7, and CCR9, indicating selective binding of **9a** to CXCR2 (Figures 3C and S5A–E). Kinetic binding studies with **9a** revealed a very slow dissociation of the ligand–receptor complex by exhibiting a rate constant of *k*_off_ = 2.14 ± 0.13 × 10^–3^ min^–1^ and a remarkably long residence time of *t*_r_ = 486 ± 28 min. For the association, we detected a rate constant of *k*_on_ = 1.10 ± 0.14 × 10^6^ M^–1^ min^–1^, resulting in a kinetic *K*_d_ value of 1.94 ± 0.27 nM (Figure 3D).

This is in good agreement with the *K*_d_ value derived from equilibrium saturation binding studies (Figure 3B). Overall, these results indicate that our squaramide-based fluorescent probe mainly gains its high CXCR2 affinity by very slow off-kinetics.

**Figure 4:**
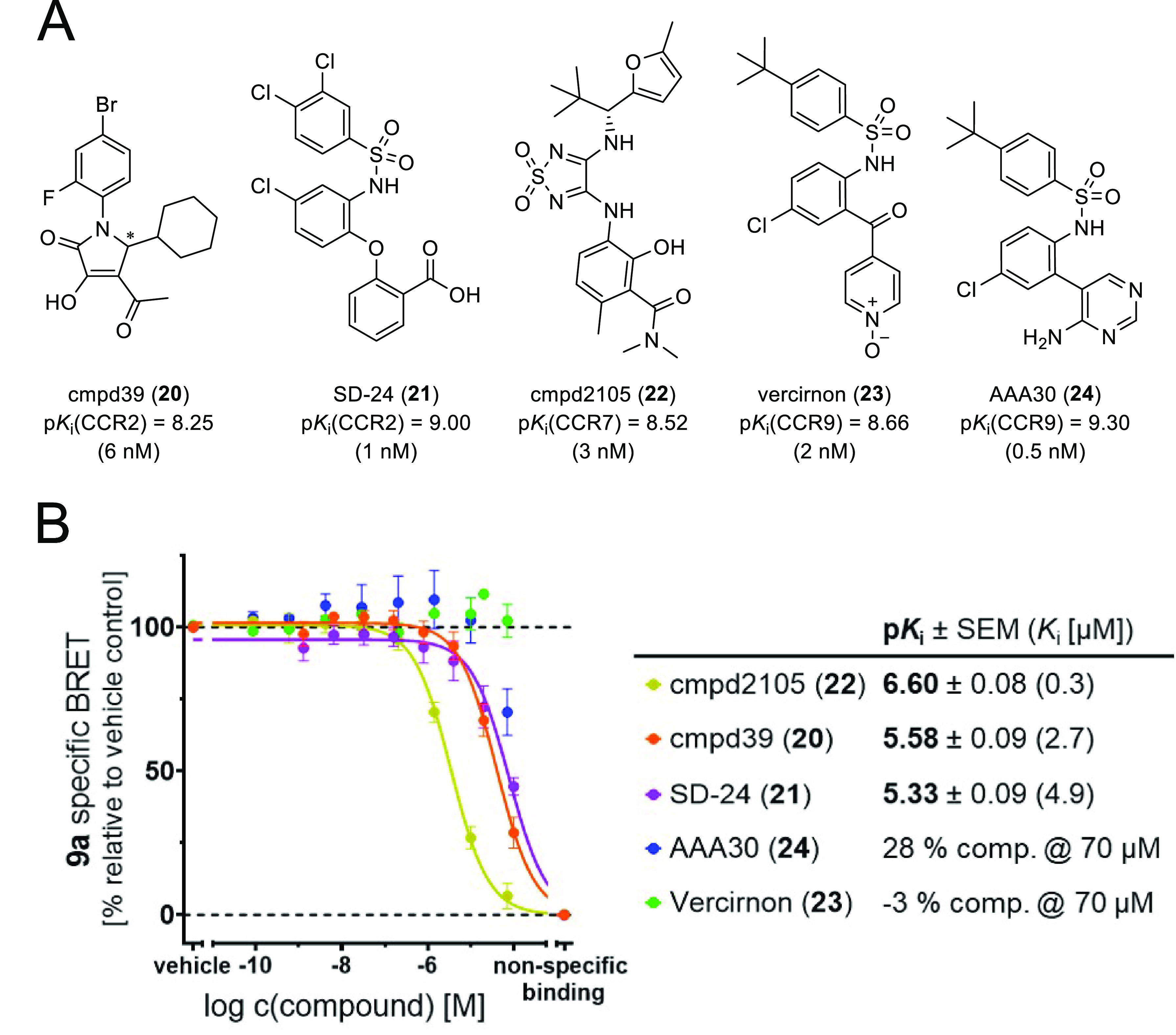
(A) Chemical structures and p*K*_i_ values (*K*_i_ values given in brackets) of known intracellular chemokine receptor antagonists **20**–**24**.^5,10,23,35,37^ (B) Competition binding curves and p*K*_i_ values (mean ± SEM, triplicate measurement, *n* = 3) for **20**–**24** obtained with **9a** (50 nM) and CXCR2_Nluc membranes. For representative competition binding curves of the single experiments, see Figure S2I.

In a next step, we aimed to investigate the suitability of **9a** as a molecular tool to study the binding of nonfluorescent ligands to the IABS of CXCR2. To this end, we set up membrane-based competition experiments with **9a** and the known intracellular CXCR2 antagonists **2**–**4** and **7** (Figures 3E and S2H). In very good agreement with the previously reported picomolar affinity for radiolabeled **2** (*K*_d_ = 0.05 nM),^34^ we obtained a p*K*_i_ value for **2** of 10.2 ± 0.1 (0.06 nM). For **3**, we detected a highly similar but slightly weaker affinity (p*K*_i_ = 10.1 ± 0.1 (0.09 nM)) compared to **2**, which is consistent with the highly similar IC_50_ values for these two compounds reported by Dwyer et al. (see Figure 1).^14^ For **4** and **7**, we determined p*K*_i_ values of 7.42 ± 0.06 (38.6 nM) and 8.45 ± 0.04 (3.58 nM), thus providing further evidence that *N*,*N*′-diarylurea-based CXCR2 antagonists are targeting the IABS as well. The orthosteric CXCR2 agonist CXCL8, also referred to as IL-8, showed no competition with **9a** (Figures 3E and S2H), thereby confirming the previously reported noncompetitive binding mode of intracellular allosteric chemokine receptor antagonists.^9,23^ To rationalize the design of our fluorescent CXCR2 ligand **9a** in a retrospective manner, we synthesized and tested the ligand–linker conjugate XI (**8**, Scheme S1, Figures 3E and S2H). Using our competition assay, we detected a sub-nanomolar CXCR2 affinity for **8** (p*K*_i_ = 9.37 ± 0.03 (0.43 nM)), thus corroborating the structure-based design of **9a**. In order to study the promiscuity of the IABS of CXCR2, we tested a selection of known intracellular allosteric antagonists targeting other chemokine receptors for their competition with **9a** (Figure 4A,B). All of these ligands, including the CCR2-targeted cmpd39 (**20**),^35^ and SD-24 (**21**),^10,36^ the CCR7-targeted cmpd2105 (**22**),^5^ as well as the CCR9-targeted vercirnon (**23**)^23,37^ and AAA30 (**24**),^23^ which were reported as antagonists with sub-nanomolar to single-digit nanomolar affinities for their targeted receptor, showed strongly reduced binding to CXCR2 (*K*_i_ ≥ 261 nM). This highlights the high potential of the IABS of CXCR2 as a target site for the development of highly selective drugs. In order to assess the quality of our cell-free NanoBRET assay and its suitability for high-throughput screening (HTS) approaches, we determined a *Z*′-factor according to Zhang et al.^38^ This resulted in a *Z*′-factor of 0.80 (Figure 3F), thereby indicating that our cell-free NanoBRET assay can be considered excellent and suitable for HTS approaches.

**Figure 5:**
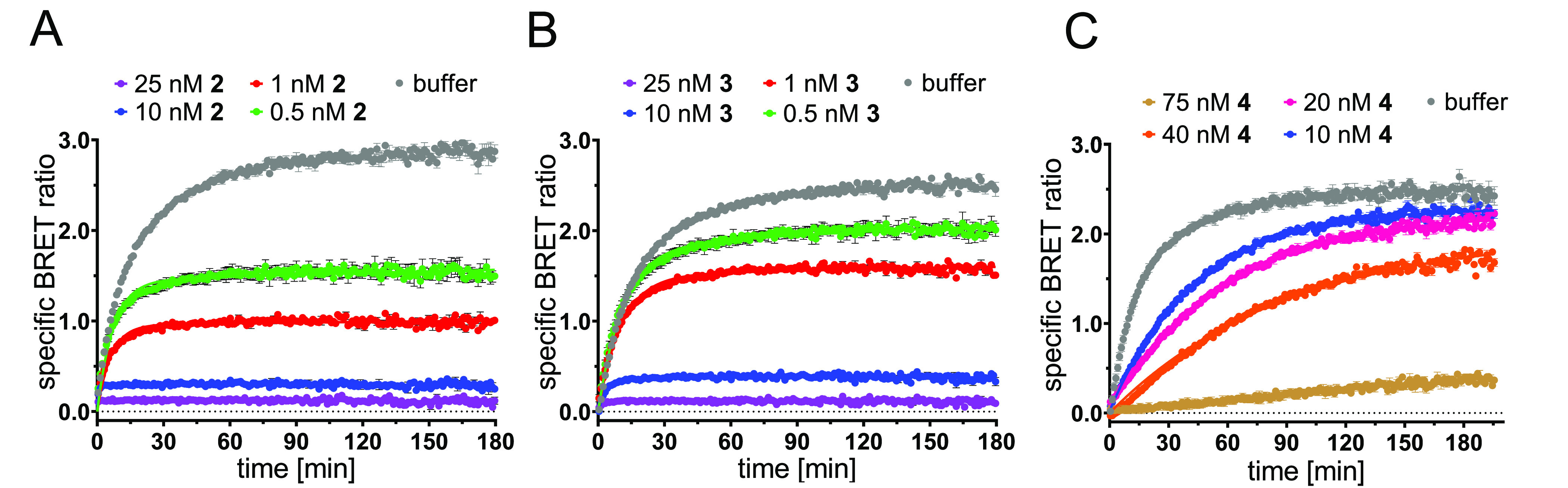
Representative kinetic competition binding curves (mean ± SEM, duplicate measurement, *n* = 3) for **2** (A), **3** (B), and **4** (C) obtained with **9a** (50 nM) and CXCR2_Nluc membranes.

In kinetic competitive binding studies with **9a** (Figure 5A–C), we detected very long residence times for the squaramide-based high-affinity ligands **2** (*t*_r_ = 462 ± 45 min) and **3** (*t*_r_ = 474 ± 59 min), which are highly consistent with the observed residence time of our fluorescent CXCR2 ligand **9a** (Figure 3D). For *N*,*N*′-diarylurea-based **4**, we found a significantly shorter residence time of 2.7 ± 0.6 min, thus rationalizing the observed affinity gap between **4** and squaramide-based ligands **2** or **3**. Association kinetics could not be deduced from kinetic competitive binding studies with **9a**. The phenomenon that fluorescent tracers with a very long residence time do not always allow a reliable determination of association kinetics, when used in a kinetic competitive binding setup, has been previously described in the literature.^39^

**Figure 6:**
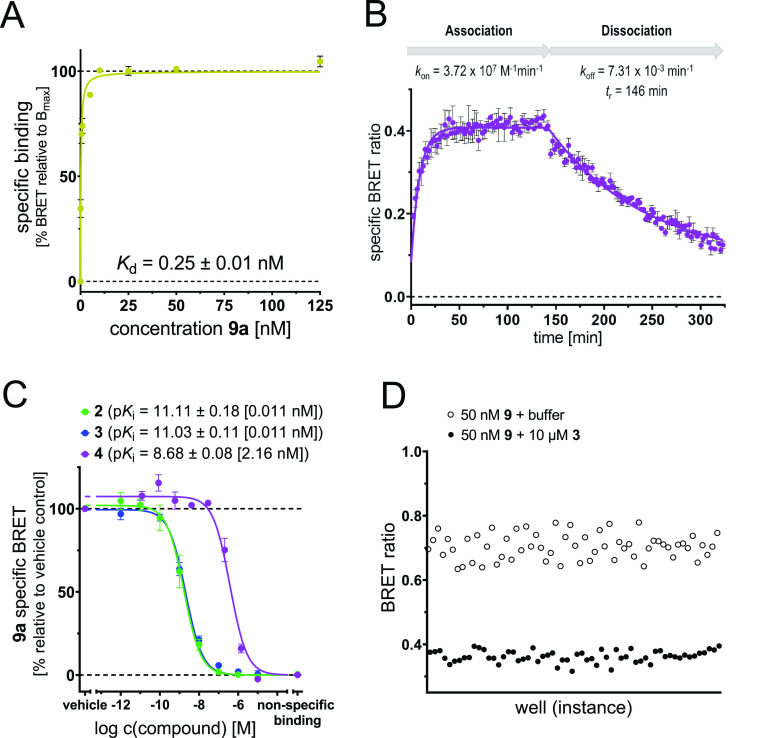
Establishment of a cellular NanoBRET-based CXCR2 binding assay, using **9a** as a fluorescent tracer and live HEK293T cells expressing CXCR2_Nluc. (A) Saturation binding curve of **9a** in a cellular NanoBRET-based experiment (mean ± SEM, quadruplicate measurement, *n* = 4). See Figure S6B for a more detailed view of the concentration range from 0 to 25 nM. (B) Representative association and dissociation curves with **9a** (5 nM, mean ± SEM, at least triplicate measurement, *n* = 4). (C) Competition binding curves and p*K*_i_ values (mean ± SEM, quadruplicate measurement) for **2** (green, *n* = 5), **3** (blue, *n* = 5), and **4** (purple, *n* = 3) obtained with **9a** (50 nM). *K*_i_ values are given in square brackets. Representative competition binding curves for the single experiments are shown in Figure S6C. (D) *Z*′-factor determination for the cell-based NanoBRET CXCR2 binding assay (60-fold determination).

Having shown that our fluorescent CXCR2 ligand **9a** is a suitable tool to study binding of high to low-affinity binders to the IABS of CXCR2 in a cell-free setup, we aimed for transferring our NanoBRET binding assay to a live cell environment. In general, the assessment of the interactions between a drug and its target protein in a cellular environment is a critical step in preclinical drug discovery.^40^ This step, also referred to as cellular target engagement, is very important for successfully delivering compounds with the desired biological and ultimately clinical effects, as the on-target activity of small molecules can be affected significantly when transitioning from a cell-free to a cellular environment. A loss of activity in a cellular environment can be attributed to various factors, for example, low cell permeability, compound efflux, or off-target protein binding. As the IABS is an intracellular binding site, and ligands targeting this binding site need to pass the cell membrane to be active in a cellular setup, cellular binding assays are of utmost importance to identify suitable candidates for further cellular and *in vivo* studies. To our best knowledge, no small-molecule tracer has been developed so far that allows cellular binding assays for the IABS of CXCR2. For our cell-based CXCR2 binding assay, we used live HEK293T cells transiently expressing CXCR2_Nluc. With this cell-based assay setup, we determined a *K*_d_ value of 0.25 ± 0.01 nM for **9a** (Figures 6A and S6A,B), thereby demonstrating that **9a** is able to pass the cell membrane and bind to CXCR2 at the intracellular side of the receptor. Cell-based kinetic binding studies with **9a** yielded a highly similar kinetic *K*_d_ value of 0.20 ± 0.02 nM (Figures 6B and S7). Compared to cell-free conditions (Figure 3D), higher rate constants for association and dissociation (*k*_on_ = 3.72 ± 0.24 × 10^7^ M^–1^ min^–1^, *k*_off_ = 7.31 ± 0.45 × 10^–3^ min^–1^) and a shorter residence time *t*_r_ = 146 ± 10 min were detected. We hypothesized that these changes in kinetic rate constants can be attributed, at least to a certain extent, to the fact that the cell-based experiments were performed at 37 °C and not at room temperature like the cell-free studies. To test this hypothesis, we also performed cell-free kinetic binding studies at 37 °C (Figure S8, Table S1C,D) and detected kinetic rate constants indicating faster association and dissociation (*k*_on_ = 1.96 ± 0.18 × 10^6^ M^–1^ min^–1^, *k*_off_ = 5.69 ± 0.23 × 10^–3^ min^–1^) compared to the measurements performed at room temperature (see Figure 3D and Table S1A,B).

However, while the detected *k*_off_ rate constant of *k*_off_ = 5.69 ± 0.23 × 10^–3^ min^–1^ is now much more similar to the cellular *k*_off_ (*k*_off_ = 7.31 ± 0.45 × 10^–3^ min^–1^), there is still a significant difference between the *k*_on_ rates obtained at 37 °C under cell-free (*k*_on_ = 1.96 ± 0.18 × 10^6^ M^–1^ min^–1^) and cellular (*k*_on_ = 3.72 ± 0.24 × 10^7^ M^–1^ min^–1^) conditions. Further, the kinetic *K*_d_ value of 2.91 ± 0.27 nM, obtained under cell-free conditions at 37 °C, is consistent with the *K*_d_ values determined with a cell-free setup at room temperature (*K*_d(kin.)_ = 1.94 ± 0.27 nM; *K*_d(eq.)_ = 4.17 ± 0.80 nM). Thus, the 9- to 19-fold differences between the *K*_d_ values detected under cell-free and cell-based (*K*_d(kin.)_ = 0.20 ± 0.02 nM; *K*_d(eq.)_ = 0.25 ± 0.01 nM) conditions, respectively, have to be explained by other reasons than the incubation temperature (see Discussion and Conclusion section). By applying **9a** in a cell-based competition binding assay, we determined picomolar to low nanomolar *K*_i_ values for literature-known intracellular CXCR2 antagonists **2** (p*K*_i_ = 11.1 ± 0.2 (0.011 nM)), **3** (p*K*_i_ = 11.0 ± 0.1 (0.011 nM)), and **4** (p*K*_i_ = 8.68 ± 0.08 (2.16 nM)); see Figure 6C. These results are in line with the affinities detected in the cell-free setup (Figure 3E). Overall, the results from live cell NanoBRET and membrane-based experiments are in good agreement with each other. Then, we also determined a *Z*′ value^38^ for our cell-based NanoBRET binding assays setup (Figure 6D). This resulted in a *Z*′ value of 0.5, which indicates that our cell-based NanoBRET assay can be considered excellent as well. Thus, our fluorescent CXCR2 ligand **9a** is a highly valuable tool to study binding to the IABS of CXCR2 in a cellular environment.

**Figure 7:**
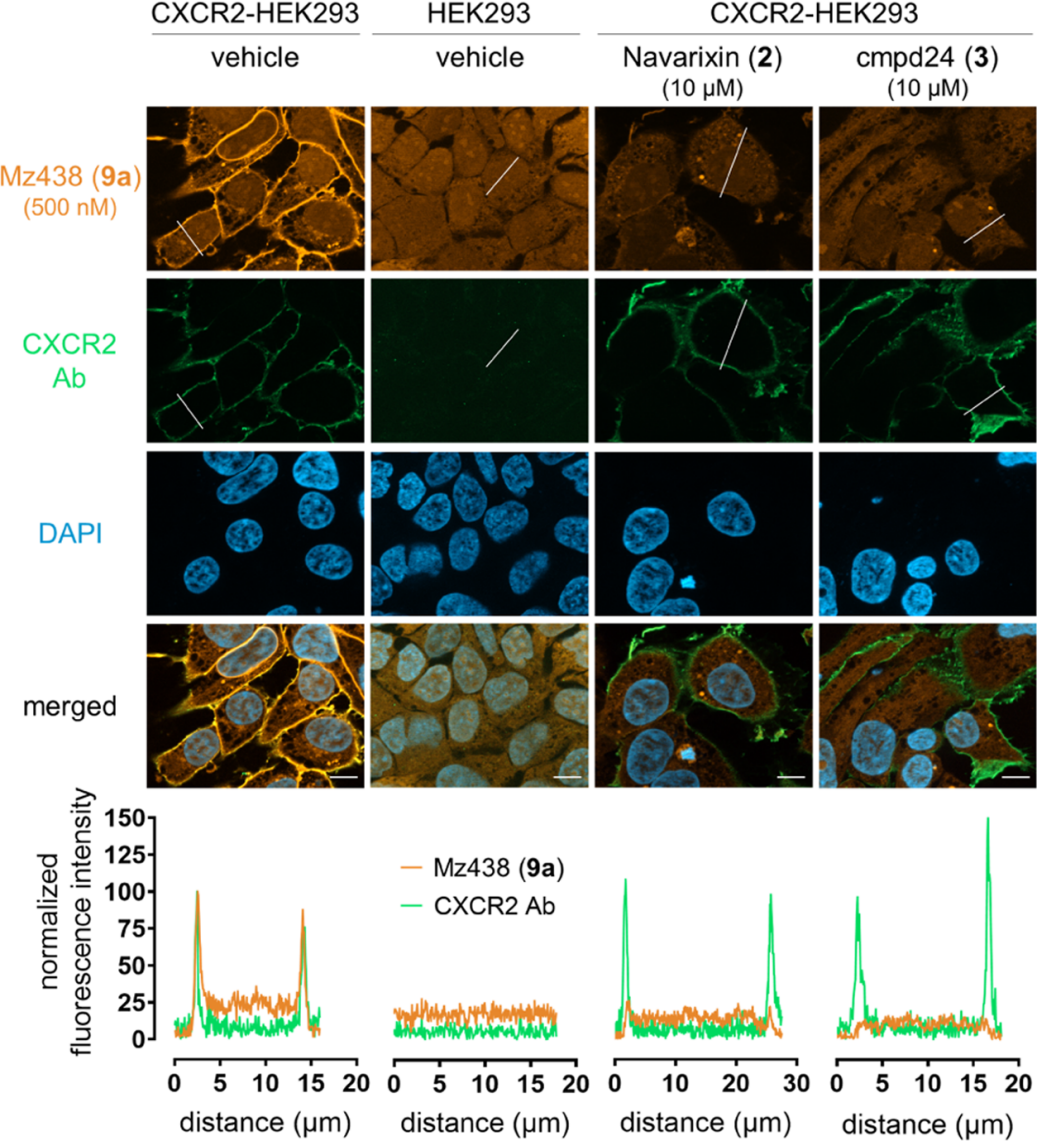
Application of **9a** (500 nM) as a tool to visualize CXCR2 and cellular target engagement of intracellular CXCR2 antagonists *via* fluorescence microscopy with fixed cells. Representative images from fixed, nonpermeabilized HEK293 cells stably expressing CXCR2 and treated according to the given conditions. Fluorescence intensity profiles along the white lines in the pictures demonstrate the colocalization of the fluorescent CXCR2 ligand and receptor immunostaining on the plasma membrane and the displacement by the competitors. Scale bar is 10 μm.

Next, we investigated the suitability of **9a** as a fluorescent tracer in order to visualize cellular target engagement for ligands targeting the IABS of CXCR2. To this end, we used **9a** in combination with fluorescence microscopy. In fixed HEK293 cells stably expressing CXCR2, the fluorescence profile of **9a** clearly reflects specific binding of the probe to plasma-membrane-resident CXCR2 (Figure 7).

**Figure 8:**
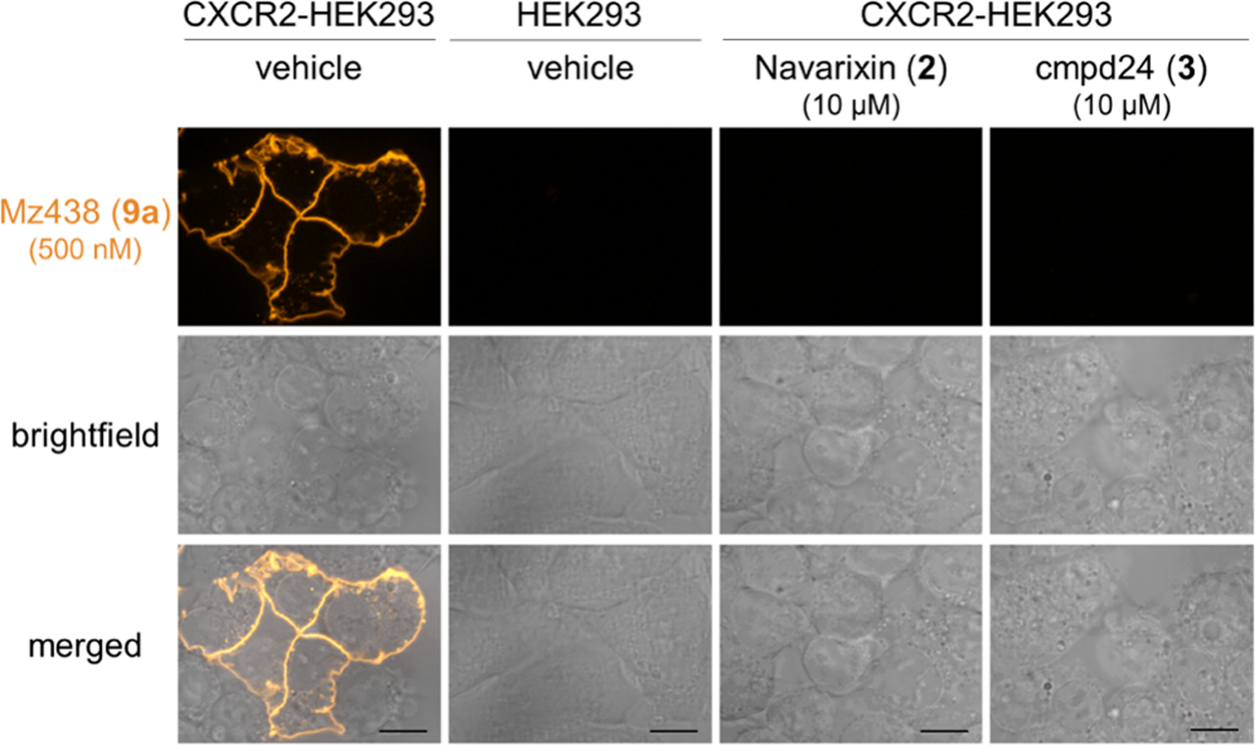
Application of **9a** (500 nM) as a tool to visualize CXCR2 and target engagement of intracellular CXCR2 antagonists via live cell imaging. Representative images from HEK293 cells stably expressing CXCR2 and untransfected cells treated according to the given conditions. Scale bar is 10 μm.

Costaining experiments with a CXCR2-directed fluorescent antibody showed a good overlap with the membrane-associated fluorescence signals for **9a** as indicated by the merged image and two overlapping sharp fluorescence peaks in line scan analysis. Entirely consistent with probe specificity, no discernable plasma membrane-associated fluorescence was observed in parental HEK293 cells, neither for **9a** nor for the CXCR2 antibody. However, parental HEK293 cells showed detectable intracellular fluorescence, indicating that **9a** accumulates in cells and does not demand CXCR2 for plasma membrane permeation. An intracellular accumulation of **9a** is further supported by the fact that no washing step was implemented between tracer incubation and imaging. Most importantly, preincubation of CXCR2-expressing cells with the nonfluorescent intracellular CXCR2 antagonists **2** or **3** virtually blunted all membrane-associated probe fluorescence but was without an effect on plasma membrane delimited CXCR2 recognition by the CXCR2-directed fluorescent antibody. These data are entirely consistent with competition between **9a** and the nonfluorescent intracellular antagonists **2** or **3** and provide strong support for the notion that **9a** is indeed a suitable tool for visualizing cellular target engagement of nonfluorescent intracellular CXCR2 antagonists. Finally, after having shown the suitability of **9a** as a tracer for fluorescence microscopy with fixed cells, we were interested whether **9a** can be applied for live cell imaging as well. Our data from fluorescence imaging with live nonfixed cells clearly indicate that **9a** can also be used to visualize CXCR2 and ligand binding to CXCR2 under these conditions (Figure 8). Consistent with the data obtained with fixed cells, an enrichment of the fluorescent tracer **9a** at the plasma membrane was observed. Almost no fluorescence signal was detected when performing the same experiment with HEK293 cells that are not expressing CXCR2. The very low background fluorescence is a consequence of both the highly specific CXCR2 binding of **9a** and a washing step that was implemented in the protocol for live cell imaging between tracer incubation and imaging. Further, preincubation with the nonfluorescent intracellular antagonist **2** or **3** led to a very strong reduction in membrane-based fluorescence. Thus, these data show the applicability of **9a** as a tracer for live cell imaging and further support highly specific binding of **9a** to CXCR2.

## Discussion and Conclusions

Starting from the cocrystal structure of CXCR2 and 00767013 (**1**), we report the development of Mz438 (**9a**) as the first small-molecule-based fluorescent probe targeting the IABS of CXCR2. In combination with the NanoBRET technique, **9a** enabled equilibrium as well as kinetic binding studies. With detected CXCR2 equilibrium *K*_d_ values of 4.17 and 0.25 nM in a cell-free and cellular setup, respectively, **9a** represents the IABS-targeted small-molecule fluorescent tracer with the highest reported affinity for its target receptor. Selectivity studies with other chemokine receptors, which are known to feature a druggable IABS (i.e., CCR2, CCR7, CCR9, CXCR1), indicated that **9a** selectively (∼50-fold) binds to CXCR2. In an equilibrium competition binding setup, **9a** was applied for the characterization of high- to low-affinity intracellular CXCR2 antagonists. Binding studies with known high-affinity IABS-targeted antagonists for other chemokine receptors revealed their strongly reduced binding to CXCR2, thus emphasizing the potential of the IABS of CXCR2 as a target site for the development of highly selective drugs. Due to its straightforward homogeneous assay protocol and a *Z*′-factor of 0.80, our membrane-based NanoBRET competition binding assay is suitable for high-throughput screening (HTS) approaches to identify new chemotypes for intracellular CXCR2 antagonists. Kinetic binding studies with our fluorescent probe (**9a**) indicated very long residence times for both the fluorescent probe as well as the unlabeled antagonists navarixin (**2**) and cmpd24 (**3**), thereby providing a rational for the very high affinities of these squaramide-based CXCR2 ligands. In addition, we show that **9a** can be used as a versatile molecular tool to study cellular target engagement for the IABS of CXCR2 *via* a cell-based NanoBRET assay and fluorescence microscopy. For fluorescence microscopy studies, **9a** was successfully applied to visualize CXCR2 and ligand binding to CXCR2, both in fixed and live cells stably expressing CXCR2, thus further highlighting the broad applicability and high specificity of **9a**. Moreover, fluorescence imaging studies with fixed HEK293 cells, where no washing step between tracer incubation and imaging was applied, suggested an accumulation of **9a** inside the cells, thereby providing a potential explanation to rationalize the difference in *K*_d_ values detected under cell-free and cell-based conditions. As the IABS of CXCR2 is a highly relevant target site for the development of anti-inflammatory and anticancer drugs, respectively, which is exemplified by the prior phase II candidate navarixin (**2**), our fluorescent tracer **9a** represents a very promising tool for future studies investigating the therapeutic potential of intracellular CXCR2 antagonists. Future studies will also be directed toward applying **9a** in a native background with cells that express CXCR2 endogenously.

## Experimental Section

### General Remarks

Starting materials (chemicals) were purchased from commercial suppliers (Abcr, Acros, Alfa Aesar, BLDpharm, Sigma-Aldrich, TCI) and used without any further purification. Solvents were used in p.a. quality and dried according to common procedures, if necessary. Literature-known reference compounds (**2**–**4, 7, 20–24**) were either purchased from commercial suppliers (Selleckchem (**2**), MedChemExpress (**4**, **7**, **22**)) or synthesized according to previously published procedures (**3**, **20**–**21**, **23**–**24**).^14,22,23^ Thin-layer chromatography (TLC) for reaction monitoring was performed with alumina plates coated with Merck silica gel 60 F254 (layer thickness: 0.2 mm) or Merck silica gel 60 RP-18 F254 (layer thickness: 0.2 mm) and analyzed under UV light (254 nm). As an alternative method for reaction monitoring, we used high-performance liquid chromatography mass spectrometry (HPLC-MS) or low-resolution electrospray ionization mass spectrometry (LRMS-ESI). HPLC-MS analyses were performed using a Thermo Scientific Dionex UltiMate 3000 HPLC system in combination with a DAD detector (220/230/254 nm) and an Agilent ZORBAX ECLIPSE, XDB-C8 column (3.0 mm × 100 mm, 3.5 μm). Elution was performed at room temperature under gradient conditions. Eluent A was water containing 0.1% (v/v) formic acid; eluent B was methanol. Linear gradient conditions were as follows: 0–0.2 min: A = 75%, B = 25%; 0.2–6.0 min: linear increase to B = 100%; 6.0–8.5 min: B = 100%; 8.5–9.0 min: linear decrease to A = 75%, B = 25%; 9.0–12.0 min: A = 75%, B = 25%. A flow rate of 0.4 mL·min^–1^ was maintained during the entire elution. Mass detection was performed with a BRUKER amaZon SL mass spectrometer using ESI as the ionization source. As an alternative method for low-resolution electrospray ionization mass spectrometry (LRMS-ESI), we used an Advion expression compact mass spectrometer (CMS) coupled with an automated TLC plate reader Plate Express (Advion). Flash column chromatography was performed with hand packed Silica Columns 60M (0.040–0.063 mm, 230–400 mesh) as a stationary phase on a Biotage SP-1 or Selekt automated flash purification system with a UV–vis detector. Yields were not optimized. NMR spectra were recorded using either a Bruker Avance 400 (^1^H: 400 MHz, ^13^C: 101 MHz), Bruker Avance DRX 500 (^1^H: 500 MHz, ^13^C: 126 MHz), or a Bruker Avance 600 (^1^H: 600 MHz, ^13^C: 151 MHz) instrument. The spectra are referenced against the NMR solvent and are reported as follows: ^1^H: chemical shift δ (ppm), multiplicity (s = singlet, d = doublet, dd = doublet of doublets, t = triplet, m = multiplet, b = broad), integration, coupling constant (*J* in Hz). ^13^C: chemical shift δ (ppm), abbreviations: carbons that could not be found in ^13^C spectra (DEPTQ) but in HMBC or HSQC are additionally marked with an asterisk (*). Signals that are partially overlaid by a solvent signal are marked with a hashtag (^#^). The assignment resulted from HMBC and HSQC experiments. High-resolution mass spectra were measured with a timsTOF Pro Mass Spectrometer from Bruker Daltonics using ESI as the ionization source. Purity was determined for all tested compounds by HPLC and UV detection and was >95%. HPLC analyses according to method 1 (M1) were performed using an Agilent 1200 series HPLC system employing a diode array detector (DAD, detection at 200, 220, 254, or 560 nm) and a ZORBAX ECLIPSE, XDB-C8 column (4.6 mm × 150 mm, 5 μm) with a flow rate of 0.5 mL·min^–1^. If not stated otherwise, the indicated purity was determined at a wavelength of 254 nm. As solvent systems, the following binary solvent systems were used. Elution was performed at room temperature under gradient conditions. Eluent A was water containing 0.1% (v/v) trifluoroacetic acid (TFA); eluent B was acetonitrile. Linear gradient conditions were as follows: 0–3.0 min: A = 90%, B = 10%; 3.0–18.0 min: linear increase to A = 5%, B = 95%; 18.0–24.0 min: A = 5%, B = 95%; 24.0–27.0 min: linear decrease to A = 90%, B = 10%; 27.0–30.0 min: A = 90%, B = 10%. HPLC analyses according to method 2 (M2) were performed using an UltiMate 3000 UHPLC system (Thermo Fisher Scientific) with a Nucleodur 100-5 C18 (250 mm × 4.6 mm, Macherey Nagel) with a flow rate of 1 mL/min and a temperature of 25 °C or a 100-5 C18 (100 mm × 3 mm, Macherey Nagel) with a flow rate of 0.5 mL/min and a temperature of 25 °C. Detection was implemented by UV absorption measurement at wavelengths of λ = 220 and 250 nm. The indicated purity was determined at a wavelength of 220 nm. Bidest H_2_O (A) and acetonitrile (B) were used as eluents with an addition of 0.1% TFA for eluent A. Elution was performed at room temperature using the following gradient conditions: column equilibration for 5 min using 95% of eluent A and 5% of eluent B, then a linear gradient from 5% B to 95% B in 7 min followed by an isocratic regime of 95% B for 10 min.

### Synthesis and Compound Characterization

Detailed information on experimental procedures for compound synthesis as well as compound characterization data are listed below. NMR spectra and HPLC chromatograms are given in the Supporting Information.

#### (*R*)-3-((2-((2,2-Dimethyl-1-(5-methylfuran-2-yl)propyl)amino)-3,4-dioxocyclobut-1-en-1-yl)amino)-2-hydroxy-*N*-((1-(2-methoxyethyl)-1*H*-1,2,3-triazol-4-yl)methyl)benzamide (**8**)

2-Hydroxy-3-((2-methoxy-3,4-dioxocyclobut-1-en-1-yl)amino)-*N*-(prop-2-yn-1-yl)benzamide (**13**, 385 mg, 1.28 mmol, 1.0 equiv) and (*R*)-2,2-dimethyl-1-(5-methylfuran-2-yl)propan-1-aminium chloride (**18**, 338 mg, 1.67 mmol, 1.3 equiv) were dissolved in methanol (8 mL). After the addition of *N,N*-diisopropyldiethylamine (456 μL, 2.56 mmol, 2.0 equiv), the reaction was stirred for 7 days. Extraction between water and ethyl acetate (3 × 50 mL), drying over sodium sulfate, filtration, and evaporation of the solvent gave the crude product that was purified by preparative HPLC (acetonitrile/water (0.1% TFA): gradient 5–95%) to obtain compound **19** as an amorphous brown solid that was used directly in the next step (212 mg, 38%). In this next step, 1-azido-2-methoxyethane (7.00 mg, 68.9 μmol, 2 equiv) and (*R*)-3-((2-((2,2-dimethyl-1-(5-methylfuran-2-yl)propyl)amino)-3,4-dioxocyclobut-1-en-1-yl)amino)-2-hydroxy-*N*-(prop-2-yn-1-yl)benzamide (**19**, 15.0 mg, 34.4 μmol, 1 equiv) were dissolved in a water/*tert*-BuOH mixture (2 mL, 1:1). Tris(benzyltriazolylmethyl)amine (TBTA, 1.83 mg, 3.44 μmol, 0.1 equiv) was dissolved in DMF (1 mL) and added to the mixture. An aqueous CuSO_4_ solution (34.4 μL, 0.1 M, 0.1 equiv) and an aqueous solution of sodium ascorbate (68.8 μL, 0.1 M, 0.2 equiv) were added in that order. The resulting reaction mixture was stirred for 2 h at room temperature. After completion of the reaction, volatiles were removed under reduced pressure. The residue was purified by preparative HPLC (acetonitrile/water (0.1% TFA): gradient 35–48%) to obtain the title compound as a colorless solid (11.9 mg, 65%). ^1^H NMR (400 MHz, DMSO-*d*_6_, δ [ppm]): 13.92 (s, 1H, −OH), 9.61 (t, ^3^*J* = 6.0 Hz, 1H, −CO–NH–CH_2_−), 9.58 (s, 1H, −NH–Ar−), 8.81 (d, ^3^*J* = 10.1 Hz, 1H, −CH(*t*-butyl)–NH−), 8.00 (s, 1H, triazole H-5), 7.97 (dd, ^3^*J* = 8.1 Hz, ^4^*J* = 1.3 Hz, 1H, 2-hydroxybenzamide H-4), 7.59 (dd, ^3^*J* = 8.1 Hz, ^4^*J* = 1.3 Hz, 1H, 2-hydroxybenzamide H-6), 6.89 (t, ^3^*J* = 8.1 Hz, 1H, 2-hydroxybenzamide H-5), 6.19 (d, ^3^*J* = 3.1 Hz, 1H, 5-methylfuran H-3), 6.05 (dd, ^3^*J* = 3.1 Hz, ^4^*J* = 1.1 Hz, 1H, 5-methylfuran H-4), 5.11 (d, ^3^*J* = 10.1 Hz, 1H, −CH(*t*-butyl)–NH−), 4.57 (d, ^3^*J* = 6.0 Hz, 2H, −CO–NH–CH_2_−), 4.50 (t, ^3^*J* = 5.6 Hz, 2H, −CH_2_–CH_2_–O–CH_3_), 3.72^#^ (t, ^3^*J* = 5.6 Hz, 2H, −CH_2_–CH_2_–O–CH_3_), 3.22 (s, 3H, −O–CH_3_), 2.28 (d, ^4^*J* = 1.1 Hz, 1H, 5-methylfuran −CH_3_), 0.97 (s, 9H, −C(CH_3_)_3_); ^13^C NMR (DEPTQ, 151 MHz, DMSO-*d*_6_, δ [ppm]): 184.12 q (3,4-dioxocyclobut-1-en C-3), 180.20 q (3,4-dioxocyclobut-1-en C-4), 169.90 q (−CO–NH–CH_2_−), 168.63 q (3,4-dioxocyclobut-1-en C-2), 163.12 q (3,4-dioxocyclobut-1-en C-1), 151.09 q (2-hydroxybenzamide C-2), 151.00 q (5-methylfuran C-5), 150.72 q (5-methylfuran C-2), 143.93 q (triazole C-4), 127.91 q (2-hydroxybenzamide C-3), 123.52 (2-hydroxybenzamide C-4), 123.43 (triazole C-5), 120.80 (2-hydroxybenzamide C-6), 118.14 (2-hydroxybenzamide C-5), 113.66 q (2-hydroxybenzamide C-1), 108.53 (5-methylfuran C-3), 106.29 (5-methylfuran C-4), 70.13 (−CH_2_–CH_2_–O–CH_3_), 60.23 (−CH(*t*-butyl)–NH−), 57.89 (−CH_2_–CH_2_–O–CH_3_), 49.14 (−CH_2_–CH_2_–O–CH_3_), 35.68 q (−C(CH_3_)_3_), 34.60 (−CO–NH–CH_2_−), 26.20 (−C(CH_3_)_3_), 13.39 (5-methylfuran −CH_3_); HRMS *m*/*z* (ESI^+^) [found: 537.2452, C_27_H_33_N_6_O_6_^+^ requires [M + H]^+^ 537.2456]; LRMS *m*/*z* (ESI^+^) 537 ([M + H]^+^, 100%), HPLC retention time 17.92 min, 98.0% (M1).

#### (*R*)-4-((2-(2-(2-(2-(4-((3-((2-((2,2-Dimethyl-1-(5-methylfuran-2-yl)propyl)amino)-3,4-dioxocyclobut-1-en-1-yl)amino)-2-hydroxybenzamido)methyl)-1*H*-1,2,3-triazol-1-yl)ethoxy)ethoxy)ethoxy)ethyl)carbamoyl)-2-(6-(dimethylamino)-3-(dimethyliminio)-3*H*-xanthen-9-yl)benzoate (**9a**)

2-Hydroxy-3-((2-methoxy-3,4-dioxocyclobut-1-en-1-yl)amino)-*N*-(prop-2-yn-1-yl)benzamide (**13**, 385 mg, 1.28 mmol, 1.0 equiv) and (*R*)-2,2-dimethyl-1-(5-methylfuran-2-yl)propan-1-aminium chloride (**18**, 338 mg, 1.67 mmol, 1.3 equiv) were dissolved in methanol (8 mL). After the addition of *N,N*-diisopropyldiethylamine (456 μL, 2.56 mmol, 2.0 equiv), the reaction was stirred for 7 days. Extraction between water and ethyl acetate (3 × 50 mL), drying over sodium sulfate, filtration, and evaporation of the solvent gave the crude product that was purified by preparative HPLC (acetonitrile/water (0.1% TFA): gradient 5–95%) to obtain compound **19** as an amorphous brown solid that was used directly in the next step (212 mg, 38%). In this next step, (*R*)-3-((2-((2,2-dimethyl-1-(5-methylfuran-2-yl)propyl)amino)-3,4-dioxocyclobut-1-en-1-yl)amino)-2-hydroxy-*N*-(prop-2-yn-1-yl)benzamide (**19**, 6.0 mg, 13.8 μmol, 1 equiv), 6-TAMRA-PEG_3_-azide (8.7 mg, 13.8 μmol, 1 equiv), and tris(benzyltriazolylmethyl)amine (TBTA, 0.73 mg, 1.38 μmol, 0.1 equiv) were dissolved in a water/*tert*-BuOH/DMF mixture (1.5 mL, 1:1:1). An aqueous CuSO_4_ solution (13.8 μL, 0.1 M, 0.1 equiv) and an aqueous solution of sodium ascorbate (27.6 μL, 0.1 M, 0.2 equiv) were added in that order. The resulting reaction mixture was stirred for 1 h at room temperature under nitrogen atmosphere. After completion, volatiles were removed under reduced pressure. The residue was purified by preparative HPLC (acetonitrile/water (0.1% TFA): gradient 30–55%) to obtain the TFA salt of the title compound as a colorless solid (8.2 mg, 50%). ^1^H NMR (600 MHz, DMSO–*d*_6_, δ [ppm]): 13.88 (s, 1H, −OH), 13.36 (bs, 1H, −COOH), 9.55 (t, ^*3*^*J* = 5.8 Hz, 1H, −CO–NH–CH_2_−), 9.53 (s, 1H, −NH–Ar−), 8.81–8.77 (m, 2H, −CH(*t*-butyl)–NH–, −O–CH_2_–CH_2_–NH–CO−), 8.29-8.26 (m, 1H, carbamoyl benzoate H-6), 8.23–8.21 (m, 1H, carbamoyl benzoate H-5), 7.98 (s, 1H, triazole H-5), 7.95 (dd, ^*3*^*J* = 8.2 Hz, ^*4*^*J* = 1.2 Hz, 1H, 2-hydroxybenzamide H-4), 7.87–7.83 (m, 1H, carbamoyl benzoate H-3), 7.55 (dd, ^*3*^*J* = 8.2 Hz, ^*4*^*J* = 1.2 Hz, 1H, 2-hydroxybenzamide H-6), 7.06–6.89 (m, 6H, 3*H*-xanthene H-1,2,4,5,7,8), 6.85 (t, ^*3*^*J* = 8.2 Hz, 1H, 2-hydroxybenzamide H-5), 6.19 (d, ^*3*^*J* = 3.1 Hz, 1H, 5-methylfuran H-3), 6.04 (dd, ^*3*^*J* = 3.1 Hz, ^*4*^*J* = 0.9 Hz, 1H, 5-methylfuran H-4), 5.11 (d, ^*3*^*J* = 10.1 Hz, 1H, −CH(*t*-butyl)–NH−), 4.54 (d, ^*3*^*J* = 5.8 Hz, 2H, −CO–NH–CH_2_−), 4.47 (t, ^*3*^*J* = 5.2 Hz, 2H, −triazole–CH_2_–CH_2_–O−), 3.76 (t, ^*3*^*J* = 5.2 Hz, 2H, −triazole–CH_2_–CH_2_–O−), 3.49^#^ (t, ^*3*^*J* = 5.9 Hz, 2H, −O–CH_2_–CH_2_–NH–CO−), 3.46–3.39^#^ (m, 10H, −O–CH_2_–CH_2_–O–CH_2_–CH_2_–O–CH_2_–CH_2_–NH–CO−), 3.24 (bs, 12H, −N–(CH_3_)_2_ & =N^+^–(CH_3_)_2_), 2.28 (d, ^*4*^*J* = 0.9 Hz, 1H, 5-methylfuran −CH_3_), 0.97 (s, 9H, −C(CH_3_)_3_); ^13^C NMRa) (DEPTQ, 151 MHz, DMSO-*d*_6_, δ [ppm]): 184.09 q (3,4-dioxocyclobut-1-en C-3), 180.19 q (3,4-dioxocyclobut-1-en C-4), 169.86 q (−CO–NH–CH_2_−), 168.63 q (3,4-dioxocyclobut-1-en C-2), 165.93* q (carbamoyl benzoate −COOH), 164.55 q (−O–CH_2_–CH_2_–NH–CO−), 163.08 q (3,4-dioxocyclobut-1-en C-1), 157.21 (q, ^*3*^*J* = 31.5 Hz, −OOC–CF_3_), 156.60* q (3*H*-xanthene C-3,6), 151.05 q (2-hydroxybenzamide C-2), 151.00 q (5-methylfuran C-5), 150.71 q (5-methylfuran C-2), 143.91 q (triazole C-4), 137.19* q (carbamoyl benzoate C-2), 133.04* q (carbamoyl benzoate C-1), 132.49* q (carbamoyl benzoate C-4), 130.93* (carbamoyl benzoate C-6), 130.48 (3*H*-xanthene C-1,8), 128.94 (carbamoyl benzoate C-5), 128.63* (carbamoyl benzoate C-3), 127.89 q (2-hydroxybenzamide C-3), 123.46, 123.46 (2-hydroxybenzamide C-4, triazole C-5), 120.73 (2-hydroxybenzamide C-6), 118.10 (2-hydroxybenzamide C-5), 114.45* (3*H*-xanthene C-2,7), 113.58 q (2-hydroxybenzamide C-1), 108.54 (5-methylfuran C-3), 106.29 (5-methylfuran C-4), 96.29 (3*H*-xanthene C-4,5), 69.62, 69.56, 69.48, 69.48 (−O–CH_2_–CH_2_–O–CH_2_–CH_2_–O−), 68.66 (−O–CH_2_–CH_2_–NHCO−), 68.65 (−triazole–CH_2_–CH_2_–O−), 60.23 (−CH(*t*-butyl)–NH−), 49.29 (−triazole–CH_2_–CH_2_–O−), 40.43 (−N–(CH_3_)_2_ & =N^+^–(CH_3_)_2_), 39.24* (−O–CH_2_–CH_2_–NHCO−), 35.68 q (−C(CH_3_)_3_), 34.57 (−CO–NH–CH_2_−), 26.19 (−C(CH_3_)_3_), 13.39 (5-methylfuran −CH_3_); ^19^F NMR (377 MHz, DMSO-*d*_6_, δ [ppm]): −73.58 (^−^OOC–CF_3_); UV–vis: λ_max_(Ex) = 561 nm, λ_max_(Em) = 612 nm, Stokes shift = 51 nm; HRMS *m*/*z* (ESI^+^) [found: 1066.4667, C_57_H_64_N_9_O_12_^+^ requires [M + H]^+^ 1066.4669]; LRMS *m*/*z* (ESI^+^) 534 ([M + 2H]^2+^), HPLC retention time 17.33 min, 98.3% (M1).

#### (*R*)-4-((2-(2-(2-(4-((3-((2-((2,2-Dimethyl-1-(5-methylfuran-2-yl)propyl)amino)-3,4-dioxocyclobut-1-en-1-yl)amino)-2-hydroxybenzamido)methyl)-1*H*-1,2,3-triazol-1-yl)ethoxy)ethoxy)ethyl)carbamoyl)-2-(6-(dimethylamino)-3-(dimethyliminio)-3*H*-xanthen-9-yl)benzoate (**9b**)

2-Hydroxy-3-((2-methoxy-3,4-dioxocyclobut-1-en-1-yl)amino)-*N*-(prop-2-yn-1-yl)benzamide (**13**, 385 mg, 1.28 mmol, 1.0 equiv) and (*R*)-2,2-dimethyl-1-(5-methylfuran-2-yl)propan-1-aminium chloride (**18**, 338 mg, 1.67 mmol, 1.3 equiv) were dissolved in methanol (8 mL). After the addition of *N,N*-diisopropyldiethylamine (456 μL, 2.56 mmol, 2.0 equiv), the reaction was stirred for 7 days. Extraction between water and ethyl acetate (3 × 50 mL), drying over sodium sulfate, filtration, and evaporation of the solvent gave the crude product that was purified by preparative HPLC (acetonitrile/water (0.1% TFA): gradient 5–95%) to obtain compound **19** as an amorphous brown solid that was used directly in the next step (212 mg, 38%). In this next step, (*R*)-3-((2-((2,2-dimethyl-1-(5-methylfuran-2-yl)propyl)amino)-3,4-dioxocyclobut-1-en-1-yl)amino)-2-hydroxy-*N*-(prop-2-yn-1-yl)benzamide (**19**, 7.0 mg, 16.1 μmol, 1 equiv), 6-TAMRA-PEG_2_-azide (10 mg, 19.3 μmol, 1.2 equiv), and tris(benzyltriazolylmethyl)amine (TBTA, 0.85 mg, 1.61 μmol, 0.1 equiv) were dissolved in a water/*tert*-BuOH/DMF mixture (1.5 mL, 1:1:1). An aqueous CuSO_4_ solution (32.2 μL, 0.1 M, 0.2 equiv) and an aqueous solution of sodium ascorbate (64.4 μL, 0.1 M, 0.4 equiv) were added in that order. The resulting reaction mixture was stirred for 1 h at room temperature under nitrogen atmosphere. After completion, volatiles were removed under reduced pressure. The residue was purified by preparative HPLC (acetonitrile/water (0.1% TFA): gradient 30–60%) to obtain the TFA salt of title compound as a colorless solid (5.4 mg, 30%). ^1^H NMR (600 MHz, DMSO-*d*_6_, δ [ppm]): 13.86 (s, 1H, OH), 13.36 (bs, 1H, −COOH), 9.61–9.40 (m, 2H, −CO–NH–CH_2_–triazole, −NH–Ar−), 8.90–8.57 (m, 2H, −CH(*t*-butyl)–NH–, −O–CH_2_–CH_2_–NH–CO−), 8.35–8.12 (m, 2H, carbamoyl benzoate H-5,6), 7.97 (s, 1H, triazole H-5), 7.95 (dd, ^*3*^*J* = 8.0, ^*4*^*J* = 1.4 Hz, 1H, 2-hydroxybenzamide H-4), 7.83 (bs, 1H, carbamoyl benzoate H-3), 7.55 (dd, ^*3*^*J* = 8.2, ^*4*^*J* = 1.4 Hz, 1H, 2-hydroxybenzamide H-6), 6.99–6.78 (m, 7H, 3*H*-xanthene H-1,2,4,5,7,8, 2-hydroxybenzamide H-5), 6.19 (d, ^*3*^*J* = 3.1 Hz, 1H, 5-methylfuran H-3), 6.10–6.02 (m, 1H, 5-methylfuran H-4), 5.11 (d, ^*3*^*J* = 10.1 Hz, 1H, −CH(*t*-butyl)–NH−), 4.52 (d, ^*3*^*J* = 5.5 Hz, 2H, −CO–NH–CH_2_–triazole), 4.43 (t, ^*3*^*J* = 5.2 Hz, 2H, −triazole–CH_2_–CH_2_–O−), 3.76 (t, ^*3*^*J* = 5.2 Hz, 2H, −triazole–CH_2_–CH_2_–O−), 3.51–3.44^#^ (m, 8H, −O–CH_2_–CH_2_–O–CH_2_–CH_2_–NH–CO−), 3.24–3.13 (m, 12H, −N–(CH_3_)_2_ & =N^+^–(CH_3_)_2_), 2.28 (s, 3H, 5-methylfuran −CH_3_), 0.97 (s, 9H, −C(CH_3_)_3_); ^13^C NMRb) (DEPTQ, 101 MHz, DMSO-*d*_6_, δ [ppm]): 184.13 q (3,4-dioxocyclobut-1-en C-3), 180.22 q (3,4-dioxocyclobut-1-en C-4), 169.90 q (−CO–NH–CH_2_–triazole−), 168.66 q (3,4-dioxocyclobut-1-en C-2), 164.61 q (−O–CH_2_–CH_2_–NH–CO−), 163.12 q (3,4-dioxocyclobut-1-en C-1), 151.11 q (2-hydroxybenzamide C-2), 151.06 q (5-methylfuran C-5), 150.73 q (5-methylfuran C-2), 143.94 q (triazole C-4), 130.34 (3*H*-xanthene C-1,8), 129.02 (carbamoyl benzoate C-5), 127.93 q (2-hydroxybenzamide C-3), 123.54 (2-hydroxybenzamide C-4, triazole C-5), 120.78 (2-hydroxybenzamide C-6), 118.17 (2-hydroxybenzamide C-5), 113.59 q (2-hydroxybenzamide C-1), 108.60 (5-methylfuran C-3), 106.36 (5-methylfuran C-4), 96.57 (3*H*-xanthene C-4,5), 69.44, 69.42, 68.68, 68.68 (−triazole–CH_2_–CH_2_–O–CH_2_–CH_2_–O–CH_2_–CH_2_–NHCO−), 60.27 (−CH(*t*-butyl)–NH−), 49.31 (−triazole–CH_2_–CH_2_–O−), 40.40 (−N–(CH_3_)_2_ & =N^+^–(CH_3_)_2_), 39.94* (−O–CH_2_–CH_2_–NHCO−), 35.74 q (−C(CH_3_)_3_), 34.59 (−CO–NH–CH_2_–triazole−), 26.24 (−C(CH_3_)_3_), 13.46 (5-methylfuran −CH_3_); ^19^F NMR (377 MHz, DMSO-*d*_6_, δ [ppm]): −73.45 (^−^OOC–CF_3_); HRMS *m*/*z* (ESI^+^): found: 1022.4403, C_55_H_60_N_9_O_11_^+^ requires [M + H]^+^ 1022.4407; LRMS *m*/*z* (ESI^+^) 512 ([M + 2H]^2+^), HPLC retention time 16.74 min, 95.8% (M1).

#### (*R*)-5-((3-(4-((3-((2-((2,2-Dimethyl-1-(5-methylfuran-2-yl)propyl)amino)-3,4-dioxocyclobut-1-en-1-yl)amino)-2-hydroxybenzamido)methyl)-1*H*-1,2,3-triazol-1-yl)propyl)carbamoyl)-2-(6-(dimethylamino)-3-(dimethyliminio)-3*H*-xanthen-9-yl)benzoate (**9c**)

2-Hydroxy-3-((2-methoxy-3,4-dioxocyclobut-1-en-1-yl)amino)-*N*-(prop-2-yn-1-yl)benzamide (**13**, 385 mg, 1.28 mmol, 1.0 equiv) and (*R*)-2,2-dimethyl-1-(5-methylfuran-2-yl)propan-1-aminium chloride (**18**, 338 mg, 1.67 mmol, 1.3 equiv) were dissolved in methanol (8 mL). After the addition of *N,N*-diisopropyldiethylamine (456 μL, 2.56 mmol, 2.0 equiv), the reaction was stirred for 7 days. Extraction between water and ethyl acetate (3 × 50 mL), drying over sodium sulfate, filtration, and evaporation of the solvent gave the crude product that was purified by preparative HPLC (acetonitrile/water (0.1% TFA): gradient 5–95%) to obtain compound **19** as an amorphous brown solid that was used directly in the next step (212 mg, 38%). In this next step, (*R*)-3-((2-((2,2-dimethyl-1-(5-methylfuran-2-yl)propyl)amino)-3,4-dioxocyclobut-1-en-1-yl)amino)-2-hydroxy-*N*-(prop-2-yn-1-yl)benzamide (**19**, 5.0 mg, 11.5 μmol, 1 equiv) and 5-TAMRA-azide (7.0 mg, 13.8 μmol, 1.2 equiv) were dissolved in a water/*tert*-BuOH/DMF mixture (1.5 mL, 1:1:1). An aqueous CuSO_4_ solution (23 μL, 0.1 M, 0.2 equiv) and an aqueous solution of sodium ascorbate (46 μL, 0.1 M, 0.4 equiv) were added in that order. The resulting reaction mixture was stirred overnight at room temperature under nitrogen atmosphere. After completion, volatiles were removed under reduced pressure. The residue was purified by preparative HPLC (acetonitrile/water (0.1% TFA): gradient 30–55%) to obtain the TFA salt of title compound as a colorless solid (5.1 mg, 41%). ^1^H NMR (600 MHz, DMSO-*d*_6_ δ [ppm]): 13.89 (s, 1H, −OH), 13.39 (bs, 1H, −COOH), 9.60 (t, ^*3*^*J* = 5.6 Hz, 1H,–CO–NH–CH_2_–triazole), 9.56 (s, 1H, −NH–Ar), 8.94 (t, ^*3*^*J* = 5.5 Hz, 1H, −CH_2_–CH_2_–CH_2_–NH–CO−), 8.79 (d, ^*3*^*J* = 10.1 Hz, 1H, −CH(*t*-butyl)–NH−), 8.65 (bs, 1H, carbamoyl benzoate H-6), 8.33–8.17 (m, 1H, carbamoyl benzoate H-4), 8.12 (s, 1H, triazole H-5), 7.96 (dd, ^*3*^*J* = 8.1 Hz, ^*4*^*J* = 1.4 Hz, 1H, 2-hydroxybenzamide H-4), 7.60 (dd, ^*3*^*J* = 8.3, ^*4*^*J* = 1.4 Hz, 1H, 2-hydroxybenzamide H-6), 7.57–7.46 (m, 1H, carbamoyl benzoate H-3), 7.17–6.67 (m, 7H, 3*H*-xanthene H-1,2,4,5,7,8 and 2-hydroxybenzamide H-5), 6.19 (d, ^*3*^*J* = 3.1 Hz, 1H, 5-methylfuran H-3), 6.04 (dd, ^*3*^*J* = 3.1, ^*4*^*J* = 1.1 Hz, 1H, 5-methylfuran H-4), 5.11 (d, ^*3*^*J* = 10.1 Hz, 1H, −CH(*t*-butyl)–NH−), 4.58 (d, ^*3*^*J* = 5.4 Hz, 2H, −CO–NH–CH_2_–triazole−), 4.46 (t, ^*3*^*J* = 7.0 Hz, 2H, −triazole–CH_2_–CH_2_–CH_2_–NHCO−), 3.40–3.35^#^ (m, 2H, −triazole–CH_2_–CH_2_–CH_2_–NHCO−), 3.28–3.08 (m, 12H, −N–(CH_3_)_2_ & =N^+^–(CH_3_)_2_), 2.28 (d, ^*3*^*J* = 1.1 Hz, 3H, 5-methylfuran −CH_3_), 2.14 (p, ^*3*^*J* = 7.0 Hz, 2H, −triazole–CH_2_–CH_2_–CH_2_–NHCO−), 0.97 (s, 9H,–C(CH_3_)_3_); ^13^C NMRc) (DEPTQ, 151 MHz, DMSO-*d*_6_ δ [ppm]): 184.11 q (3,4-dioxocyclobut-1-en C-3), 180.21 q (3,4-dioxocyclobut-1-en C-4), 169.91 q (−CO–NH–CH_2_–triazole), 168.64 q (3,4-dioxocyclobut-1-en C-2), 164.80 q (−CH_2_–CH_2_–CH_2_–NH–CO−), 163.12 q (3,4-dioxocyclobut-1-en C-1), 157.63 (q, ^*3*^*J* = 31.2 Hz, ^–^OOC–CF_3_), 151.11 q (2-hydroxybenzamide C-2), 151.00 q (5-methylfuran C-5), 150.72 q (5-methylfuran C-2), 144.00 q (triazole C-4), 135.91 q (carbamoyl benzoate C-2), 130.25 (3*H*-xanthene C-1,8), 127.90 q (2-hydroxybenzamide C-3), 123.56 (2-hydroxybenzamide C-4), 123.26 (triazole C-5), 120.84 (2-hydroxybenzamide C-6), 118.14 (2-hydroxybenzamide C-5), 113.68 q (2-hydroxybenzamide C-1), 108.53 (5-methylfuran C-3), 106.29 (5-methylfuran C-4), 96.50 (3*H*-xanthene C-4,5), 60.23 (−CH(*t*-butyl)–NH−), 47.36 (−triazole–CH_2_–CH_2_–CH_2_–NHCO−), 40.36 (−N–(CH_3_)_2_ & =N^+^–(CH_3_)_2_), 36.82 (−triazole–CH_2_–CH_2_–CH_2_–NHCO−), 35.68 q (−C(CH_3_)_3_), 34.66 (−CO–NH–CH_2_–triazole−), 29.76 (−triazole–CH_2_–CH_2_–CH_2_–NHCO−), 26.20 (−C(CH_3_)_3_), 13.39 (5-methylfuran −CH_3_); ^19^F NMR (377 MHz, DMSO-*d*_6_, δ [ppm]): −73.48 (^−^OOC–CF_3_); HRMS *m*/*z* (ESI^+^): found: 948.4044, C_52_H_54_N_9_O_9_^+^ requires [M + H]^+^ 948.4039; LRMS *m*/*z* (ESI^+^) 475 ([M + 2H]^2+^), HPLC retention time 17.03 min, 95.3% (M1).

#### 2-Hydroxy-3-nitro-*N*-(prop-2-yn-1-yl)benzamide (**11**)

2-Hydroxy-3-nitrobenzoic acid (1.50 g, 8.03 mmol, 1.0 equiv), bromo-tris-pyrrolidino-phosphonium hexafluorophosphate (4.54 g, 9.63 mmol, 1.2 equiv) and *N,N*-diisopropyldiethylamine (5.59 mL, 32.1 mmol, 4.0 equiv) were dissolved in dichloromethane (30 mL) and stirred at room temperature for 30 min. Propargylamine (734 μL, 11.2 mmol, 1.4 equiv) was added and the resulting mixture was stirred overnight. The reaction mixture was extracted with 1 M sodium hydroxide solution (3 × 100 mL) and the organic layer was discarded. The aqueous layer was then acidified using 1 M hydrochloric acid (300 mL) and extracted with ethyl acetate (3 ×150 mL). The combined organic layer was then dried over sodium sulfate, filtered, and concentrated *in vacuo* to afford the crude product, which was then purified by column chromatography using a mixture of dichloromethane, methanol, and trifluoroacetic acid (99:0.9:0.1 (v/v)). The title compound was obtained as a pale-yellow solid (1.39 g, 79%). ^1^H NMR (600 MHz, DMSO-*d*_6_ δ [ppm]): 13.96 (s, 1H, −OH), 9.65 (t, ^3^*J* = 5.5 Hz, 1H, −CO–NH–CH_2_−), 8.17 (dd, ^3^*J* = 8.0, ^*4*^*J* = 1.6 Hz, 1H, 2-hydroxybenzamide H-4), 8.10 (dd, ^3^*J* = 8.0, ^*4*^*J* = 1.6 Hz, 1H, 2-hydroxybenzamide H-6), 7.09 (t, ^3^*J* = 8.0 Hz, 1H, 2-hydroxybenzamide H-5). 4.17–4.10 (m, 2H, −CO–NH–CH_2_−), 3.22 (t, ^*3*^*J* = 2.5 Hz, −C≡C–H); ^13^C NMR (151 MHz, DMSO-*d*_6_ δ [ppm]): 168.35 q (−CO–NH–CH_2_−), 154.48 q (2-hydroxybenzamide C-2), 138.44 q (2-hydroxybenzamide C-3), 132.60 (2-hydroxybenzamide C-4), 129.40 (2-hydroxybenzamide C-6), 118.06 (2-hydroxybenzamide C-5), 117.20 q (2-hydroxybenzamide C-1), 79.92 q (−C≡C–H), 73.72 (−C≡C–H), 28.60 (−CO–NH–CH_2_−); LRMS *m*/*z* (ESI^+^) [found: 221.1, C_10_H_9_N_2_O_4_^+^ requires [M + H]^+^ 221.1]; *R_f_*: 0.65 (dichloromethane/methanol/trifluoroacetic acid) (99:0.9:0.1 (v/v)); mp: 125–128 °C.

#### 3-Amino-2-hydroxy-*N*-(prop-2-yn-1-yl)benzamide (**12**)

2-Hydroxy-3-nitro-*N*-(prop-2-yn-1-yl)benzamide (**11**, 450 mg, 1.94 mmol, 1.0 equiv) was dissolved in methanol (10 mL). After the addition of SnCl_2_·2 H_2_O (2.24 g, 9.71 mmol, 5.0 equiv), the reaction mixture was heated under reflux for 3 h. The reaction was then allowed to cool to room temperature, diluted with a half-saturated sodium bicarbonate solution (50 mL) and extracted with ethyl acetate (3 × 50 mL). The combined organic layer was dried over sodium sulfate, filtered, and dried *in vacuo* to afford the title compound as a green gum (327 mg, 89%), which was used without further purification. ^1^H NMR (600 MHz, DMSO-*d*_6_ δ [ppm]): 9.13 (t, ^3^*J* = 5.6 Hz 1H, −CO–NH–CH_2_−), 7.03 (dd, ^3^*J* = 7.9, ^4^*J* = 1.5 Hz, 1H, 2-hydroxybenzamide H-4), 6.77 (dd, ^3^*J* = 7.9, ^4^*J* = 1.5 Hz, 1H, 2-hydroxybenzamide H-6), 6.62 (t, ^3^*J* = 7.9 Hz, 1H, 2-hydroxybenzamide H-5), 4.16–3.97 (m, 2H, −CO–NH–CH_2_−), 3.14 (t, ^*3*^*J* = 2.5 Hz, −C≡C–H), the −NH_2_ and −OH protons could not be detected due to H/D exchange; ^13^C NMR (151 MHz, DMSO-*d*_6_ δ [ppm]): 170.32 q (−CO–NH–CH_2_−), 148.54 q (2-hydroxybenzamide C-2), 137.68 q (2-hydroxybenzamide C-3), 118.33 (2-hydroxybenzamide C-4), 116.84 (2-hydroxybenzamide C-6), 113.60 (2-hydroxybenzamide C-5), 112.75 q (2-hydroxybenzamide C-1), 80.68 q (−C≡C–H), 73.01 (−C≡C–H), 28.19 (−CO–NH–CH_2_−); LRMS *m*/*z* (ESI^+^) [found: 191.2, C_10_H_11_N_2_O_2_^+^ requires [M + H]^+^ 191.2]; *R_f_*: 0.82 (ethyl acetate).

#### 2-Hydroxy-3-((2-methoxy-3,4-dioxocyclobut-1-en-1-yl)amino)-*N*-(prop-2-yn-1-yl)benzamide (**13**)

3-Amino-2-hydroxy-*N*-(prop-2-yn-1-yl)benzamide (**12**, 310 mg, 1.63 mmol, 1.0 equiv) and dimethyl squarate were dissolved in methanol (10 mL) and stirred for 3 days. The mixture was filtered under vacuum and the solid was washed with methanol (3 × 10 mL). Drying of the solid product *in vacuo* afforded the title compound as a green solid (445 mg, 91% yield). ^1^H NMR (600 MHz, DMSO-*d*_6_ δ [ppm]): 13.21 (s, 1H, −OH), 10.22 (s, 1H, aryl–NH−), 9.46 (t, ^3^*J* = 5.6 Hz, 1H, −CO–NH–CH_2_−), 7.757.70 (m, 1H, 2-hydroxybenzamide H-4), 7.43–7.38 (m, 1H, 2-hydroxybenzamide H-6), 6.90 (t, ^3^*J* = 7.9 Hz, 1H, 2-hydroxybenzamide H-5), 4.29 (s, 3H, −O–CH_3_), 4.13–4.08 (m, 2H, −CO–NH–CH_2_−), 3.18 (t, ^3^*J* = 2.5 Hz, 1H, −C≡C–H); ^13^C NMR (151 MHz, DMSO-*d*_6_ δ [ppm]): 188.1 q (3,4-dioxocyclobut-1-en C-3), 184.3 q (3,4-dioxocyclobut-1-en C-4), 178.6 q (3,4-dioxocyclobut-1-ene C-1), 170.6 q (−CO–NH–CH_2_−), 169.4 q (3,4-dioxocyclobut-1-en C-2), 154.4 q (2-hydroxybenzamide C-2), 128.4 q (2-hydroxybenzamide C-3), 126.1 (2-hydroxybenzamide C-4), 124.3 (2-hydroxybenzamide C-6), 117.3 (2-hydroxybenzamide C-5), 114.4 q (2-hydroxybenzamide C-1), 80.3 q (−C≡C–H), 73.4 (−C≡C–H), 60.3 (−O–CH_3_), 28.4 (−CO–NH–CH_2_−); LRMS *m*/*z* (ESI^+^) [found: 301.0, C_15_H_13_N_2_O_5_^+^ requires [M + H]^+^ 301.1]; *R_f_*: 0.38 (cyclohexane/ethyl acetate (1:1) (v/v)); mp: 200–214 °C.

#### (*S*)-*N*-((*R*)-2,2-Dimethyl-1-(5-methylfuran-2-yl)propyl)-2-methylpropane-2-sulfinamide (**17**)

(*S*)-2-Methylpropane-2-sulfinamide (**15**) was purchased from BLDpharm and the determined specific optical rotation [α]_D_^20^ = −4.5 (*c* 1.0, CHCl_3_) was in agreement with the literature,^41^ thereby confirming the enantiopurity of **15**. (*S*)-2-Methylpropane-2-sulfinamide (**15**, 2.00 g, 16.2 mmol, 1.0 equiv) was dissolved in dichloromethane (10 mL). 5-Methylfuran-2-carboxaldehyde (**14**, 1693 μL, 16.17 mmol, 1.0 equiv), titanium ethoxide (7459 μL, 35.58 mmol, 2.2 equiv), and sodium sulfate (2 g) were added under stirring. The reaction mixture was stirred at room temperature overnight, filtered through celite, and rinsed with dichloromethane. Evaporation of the solvent gave the crude (*S*,*E*)-2-methyl-*N*-((5-methylfuran-2-yl)methylene)propane-2-sulfinamide (**16**) in quantitative yield, which was used for the next step without further purification. In this next step, *tert*-butyl magnesium chloride (1 M solution, 35.6 mL, 35.60 mmol, 2.2 equiv) was diluted with tetrahydrofuran (35.6 mL) to obtain a concentration of 0.5 M and cooled to 0 °C. (*S*,*E*)-2-Methyl-*N*-((5-methylfuran-2-yl)methylene)propane-2-sulfinamide (**16**, 3.50 g, 16.2 mmol, 1.0 equiv) was dissolved in tetrahydrofuran (40 mL) to obtain a concentration of 0.4 M. The solution of **16** was then added dropwise to the vigorously stirred solution of the Grignard agent and the resulting mixture was stirred for 3 days. The reaction was quenched by the addition of saturated ammonium chloride solution (20 mL) and extracted between water and ethyl acetate (3 × 75 mL). Drying over sodium sulfate, filtration, and evaporation of the solvent resulted in the crude product, which was purified by column chromatography using a gradient of cyclohexane, dichloromethane, and ethyl acetate (cyclohexane/dichloromethane (7:3) to cyclohexane/dichloromethane (5:5) to cyclohexane/dichloromethane/ethyl acetate (4.5:4.5:1) (v/v)). The title compound was obtained as a dark orange to red oil with over 95% purity of the depicted diastereomeric configuration (2.39 g, 54%). [α]_D_^25^ = +170° (*c* 0.05, methanol). ^1^H NMR (600 MHz, DMSO-*d*_6_ δ [ppm]): 6.11 (d, ^3^*J* = 3.1 Hz, 1H, 5-methylfuran H3), 5.96 (dd, ^*3*^*J* = 3.1, ^4^*J* = 1.1 Hz, 1H, 5-methylfuran H-4), 4.64 (d, ^3^*J* = 6.3 Hz, 1H, −CH(*t*-butyl)–NH−), 3.94 (d, ^3^*J* = 6.3 Hz, 1H, −CH(*t*-butyl)–NH−), 2.21 (d, ^4^*J* = 1.1 Hz, 3H, 5-methylfuran −CH_3_), 1.05 (s, 9H, −NH–SO–C(CH_3_)_3_), 0.92 (s, 9H, −CH(C(CH_3_)_3_)–NH−); ^13^C NMR (151 MHz, DMSO-*d*_6_ δ [ppm]): 152.46 q (5-methylfuran C-5), 149.93 q (5-methylfuran C-2), 108.46 (5-methylfuran C-3), 106.02 (5-methylfuran C-4), 62.86 (−CH(C(CH_3_)_3_)–NH−), 55.29 q (−NH–SO–C(CH_3_)_3_), 35.31 (−CH(C(CH_3_)_3_)–NH−), 26.52 (−NH–SO–C(CH_3_)_3_), 22.20 (−CH(C(CH_3_)_3_)–NH−), 13.24 (5-methylfuran −CH_3_); LRMS *m*/*z* (ESI^+^) [found: 272.3, C_14_H_26_NO_2_S^+^ requires [M + H]^+^ 272.2]; *R_f_*: 0.14 (cyclohexane/dichloromethane (7:3) (v/v)); HPLC retention time 14.31 min, 95.0% (M2).

#### (*R*)-2,2-Dimethyl-1-(5-methylfuran-2-yl)propan-1-aminium Chloride (**18**)

(*S*)-*N*-((*R*)-2,2-Dimethyl-1-(5-methylfuran-2-yl)propyl)-2-methylpropane-2-sulfinamide (**17**, 845 mg, 3.1 mmol, 1.0 equiv) was dissolved in diethylether (10 mL). After the addition of 1 M hydrogen chloride in diethylether (7.2 mL, 7.2 mmol, 2.3 equiv) at 0 °C, the mixture was stirred for 2 h. The solids were then filtered under vacuum to obtain the title compound as a beige-brown solid (293 mg, 46%). ^1^H NMR (600 MHz, DMSO-*d*_6_ δ [ppm]): 6.36 (d, ^3^*J* = 3.2 Hz, 1H, 5-methylfuran H-3), 6.07 (dd, ^3^*J* = 3.2 Hz, ^4^*J* = 1.1 Hz, 1H, 5-methylfuran H-4), 4.16 (s, 1H, −CH(C(CH_3_)_3_)–NH_3_^+^), 2.31 (d, ^4^*J* = 1.1 Hz, 3H, 5-methylfuran −CH_3_), 1.06 (s, 9H, −C(CH_3_)_3_); ^13^C NMR (151 MHz, DMSO-*d*_6_ δ [ppm]): 154.30 q (5-methylfuran C-5), 148.11 q (5-methylfuran C-2), 112.29 (5-methylfuran C-3), 107.55 (5-methylfuran C-4), 59.45 (−CH(C(CH_3_)_3_)–NH_3_^+^), 35.25 q (−C(CH_3_)_3_), 26.65 (−C(CH_3_)_3_), 13.40 (5-methylfuran −CH_3_); LRMS *m*/*z* (ESI^+^) [found: 168.3, C_10_H_18_NO^+^ requires [M + H]^+^ 168.2]; *R_f_*: 0.17 (cyclohexane/ethyl acetate (1:1) (v/v))*;* mp: 218–223 °C. This compound has been described in a patent by Chen et al. as a starting material;^42^ however, no compound characterization data has been reported thus far. The free base of this compound, including ^1^H NMR characterization data, was already reported by Dwyer et al.^14^

### Cell Culture

HEK293T cells (gift from Chair of Physiology, Prof. Dr. Alzheimer, FAU Erlangen-Nürnberg) were grown on 10 cm culture dishes at 37 °C and 5% CO_2_. As growth medium, Dulbecco’s Modified Eagle Medium (DMEM)/F12 (Invitrogen) supplemented with 10% fetal bovine serum (FBS, Gibco fetal bovine serum, qualified, Brazil), l-glutamine (final concentration: 2 mM; from Gibco l-glutamine 200 mM, 100×), penicillin (final concentration: 100 units/mL), and streptomycin (final concentration: 100 μg/mL; from Gibco penicillin–streptomycin 10,000 U/mL) were used. Cells were split every 3–4 days and regularly confirmed to be free of mycoplasma contamination using the luminescence-based MycoAlert Plus Kit (Lonza).

### Transient Transfection Using Polyethylenimine (PEI)

HEK293T cells were plated onto culture dishes (Ø 10 cm or Ø 15 cm) and grown to a confluence of ∼50% at 37 °C and 5% CO_2_. The growth medium was renewed 1 h before transfection. The transfection mix was prepared as described in the following. Solution A (0.4–2.2% total DNA (1–5.5 μg) in Gibco phosphate-buffered saline, pH 7.4) and solution B (3% PEI (linear, 25 kDa, from Polysciences) solution prepared from a PEI stock solution (0.4 μg/μL) in phosphate-buffered saline (PBS) without MgCl_2_ and CaCl_2_) were mixed one to one, the resulting mixture was vortexed for 5 s, and incubated for 30 min at room temperature. The preincubated transfection mix was added dropwise to the cells and cell cultivation was continued at 37 °C and 5% CO_2_.

### Membrane Preparation

Membranes from HEK293T cells transiently expressing the respective GPCR were prepared as follows. The medium of the transfected cells was refreshed after 24 h before cells were harvested 48 h post-transfection. The growth medium was removed, and the cells were carefully washed with cold phosphate-buffered saline (10 mL per Ø 15 cm dish). The cells were detached with 15 mL of ice-cold Tris-EDTA buffer (10 mM Tris, 0.5 mM EDTA, 5.4 mM KCl, 140 mM NaCl, pH 7.4) and subsequently centrifuged with 218*g* for 8 min. The supernatant was removed and the cells were resuspended in 10 mL of Tris-EDTA buffer. The cells were lysed with an Ultraturrax (20,000 rpm) used five times for 5 s with a 25 s break on ice in between. The lysate was centrifuged at 50,830*g* for 18 min at 4 °C. The supernatant was discarded, and the pellet was homogenized in a membrane buffer (50 mM Tris, 1 mM EDTA, 5 mM MgCl_2_, 100 μg/mL bacitracin, 5 μg/mL soybean trypsin inhibitor, pH 7.4) with a glass-teflon homogenizer. Aliquots of 250 μL were shock-frozen in liquid nitrogen and directly stored at −80 °C. Finally, the protein concentration was determined using the Lowry method.^43^

### cDNA Constructs

The CXCR2-Nluc fusion constructs in pcDNA3.1 were generated using the Gibson Assembly^44^ (New England Biolabs) method. Therefore, the sequences of the Nluc enzyme^33^ (pNLF1-C, Promega) and (3xHA)-tagged CXCR2 (CXCR2, cdna.org, #CXCR20TN00) were amplified by polymerase chain reaction and were directly fused in frame (no linker). DNA sequencing was performed to verify sequence integrity (Eurofins Genomics). Plasmids were cloned into *E. coli* DH5-α (New England Biolabs) and purified using a Maxiprep DNA purification kit (Invitrogen). For selectivity studies with CCR2, CCR7, CCR9, and CXCR1, we modified the procedure described above. Instead of (3xHA)-tagged CXCR2 (cdna.org, #CXCR20TN00), we used (3xHA)-tagged CCR2 (cdna.org, #CCR020TN00), (3xHA)-tagged CCR7 (cdna.org, #CCR070TN00), (3xHA)-tagged CCR9 (cdna.org, #CCR090TN00), and (3xHA)-tagged CXCR1 (cdna.org, #CXCR10TN00), respectively. For CCR2 and CCR7, we inserted a GSSG linker between the C-terminus of the respective receptor and the Nluc-tag, whereas for CXCR1, CXCR2, and CCR9 no linker was used. The constructs for the Nluc-labeled CCR2 and CCR9 were already published.^22,23^

### ELISA

HEK293T cells were transfected with the plasmid encoding 3xHA-CXCR2 or with the 3xHA-tagged CXCR2_Nluc construct using polyethylenimine in suspension. Therefore, HEK293T cells were detached from their culture plates and diluted to a density of 3 × 10^5^ cells/mL in the growth medium. This cell suspension was mixed with the preformed transfection mix (PEI/DNA ratio 3:1) consisting of 1.0 μg of receptor cDNA plasmid and 1.0 μg of single-stranded salmon sperm DNA (ssDNA, Sigma-Aldrich) in phosphate-buffered saline (PBS) per 2 mL of cell suspension. Subsequently, cells were transferred to a 48-well plate (7.5 × 10^4^ cells/well), which was pretreated with poly-d-lysine (0.1 mg/mL, dissolved in water). The cells were incubated for 48 h at 37 °C and 5% CO_2_. On the day of the assay, the medium was removed, and the cells were incubated with 200 μL/well of ROTIHistofix 4% fixation solution (Carl Roth) for 10 min at room temperature. The cells were washed once with 300 μL of washing buffer for 2 min (150 mM NaCl, 25 mM Tris, pH 7.5) and blocked for 1 h using 800 μL of blocking buffer (30 g/L skim milk powder in washing buffer). After removal of the blocking solution, 200 μL/well of anti-HA rabbit IgG antibody (Sigma-Aldrich, catalog # H6908, 1:4000 in blocking solution) was added. After 60 min of incubation, wells were washed twice for 2 min (300 μL/well) and blocked again for 1 h at room temperature, before 200 μL/well anti-rabbit IgG-HRP antibody (Invitrogen by Thermo Fisher Scientific, catalog # G-21234, 1:1000 in blocking solution) was added. After incubation for 1 h, the cells were washed three times for 2 min (300 μL/well) before the substrate reaction was initiated by the addition of the substrate buffer (6 mM *o*-phenylenediamine in 35 mM citric acid, 66 mM Na_2_HPO_4_, pH 5.0). After 15 min incubation in the dark, the reactions were terminated by the addition of 1 M H_2_SO_4_ (200 μL/well). For each well, 2 × 150 μL of the resulting mixture was transferred to a clear, flat-bottom 96-well plate and absorption was measured at 492 nm in a microplate reader. The measured absorbance values were baseline-corrected using cells transfected with a nontagged muscarinic receptor (M3R, cdna.org) as negative control. These baseline-corrected values were normalized to 3xHA-CXCR2 expression. For selectivity studies with CCR2, CCR7, CCR9, and CXCR1, we modified the procedure described above. Instead of the plasmids encoding CXCR2_Nluc, we used plasmids encoding the corresponding Nluc-labeled constructs for CCR2,^22^ CCR7, CCR9,^23^ and CXCR1.

### Emission and Excitation Spectra of Fluorescent Ligands

For the excitation spectra, the fluorescent ligands (**9a**–**c**) were diluted to 500 μM in aqueous solution and 25 μL of these solutions were pipetted into a 384-well plate. Then, excitation spectra were measured with a CLARIOstar microplate reader. The emission spectra of the fluorescent ligands (1 mM in DMSO) were recorded using a CLARIOstar (BMG Labtech, Ortenberg, Germany) microplate reader and 480 nm as excitation wavelength.

### Emission Spectra of Nluc-Labeled Chemokine Receptors (CXCR2, CCR7, CXCR1)

Furimazine (Promega, Mannheim, Germany 1:2000) was added to the membrane preparations (2–6 μg protein/well). After 5 min incubation in the dark, the emission spectra were measured ranging from 350 to 700 nm using a CLARIOstar (BMG Labtech, Ortenberg, Germany) microplate reader.

### NanoBRET Binding Assays

#### Membrane-Based NanoBRET Saturation Assay

For the establishment of our NanoBRET binding assay, we referred to a recently published protocol.^45^ The fluorescent ligands (**9a**–**c**) were dissolved in DMSO (1 mM) and further diluted to varying concentrations in an assay buffer (50 mM Na_2_HPO_4_, 50 mM KH_2_PO_4_, pH 7.4, 1 mg/mL saponin, 5% FBS) and 5 μL of these dilutions were pipetted to a 384-well plate. To determine the total binding, 5 μL of the assay buffer was added to the corresponding wells, while 5 μL of a solution of **3** in the assay buffer (final assay concentration: 1 μM) was used to determine nonspecific binding. Then, 20 μL of the membrane preparations diluted in the assay buffer (2 μg total protein/well) were added and the plates were incubated for 90 min at 37 °C. Subsequently, 5 μL of a furimazine solution (Promega, Mannheim, Germany, final assay dilution: 1:5000) was added to each well (final assay volume: 35 μL) before measuring luminescence with a CLARIOstar microplate reader using 620/10 and 475/30 nm emission filters after 5 min of incubation in the dark. Bioluminescence resonance energy transfer (BRET) was determined as the ratio of acceptor fluorescence and donor luminescence. The algorithms for one-site saturation binding from PRISM9.5 (GraphPad) were utilized to analyze total, nonspecific, and specific binding. Specific binding signals were calculated as a difference of total and nonspecific binding. If required, netBRET values were calculated as the difference between total BRET values and the values obtained in the absence of a fluorescent ligand. For selectivity studies with CCR2, CCR7, CCR9, and CXCR1, we modified the procedure described above.

For CCR2, we used membrane preparations from HEK293T cells expressing the published CCR2_GSSG_Nluc construct^22^ (4 μg total protein/well) and CCR2-RA (final assay concentration: 10 μM) as a known intracellular CCR2 antagonist^35^ to determine nonspecific binding.

For CCR7, we used membrane preparations from HEK293T cells expressing the aforementioned CCR7_GSSG_Nluc construct (3 μg total protein/well) and cmpd2105 (**22**, final assay concentration: 25 μM) as a known intracellular CCR7 antagonist to determine nonspecific binding.

For CCR9, we used membrane preparations from HEK293T cells expressing the published CCR9_Nluc construct^23^ (2 μg total protein/well) and vercirnon (**23**, final assay concentration: 10 μM) as a known intracellular CCR9 antagonist to determine nonspecific binding.

For CXCR1, we used membrane preparations from HEK293T cells expressing the aforementioned CXCR1_Nluc construct (4 μg total protein/well) and navarixin (**2**, final assay concentration: 10 μM) as a known intracellular CXCR1 antagonist^14^ to determine nonspecific binding.

#### Membrane-Based NanoBRET Competition Assay

The fluorescent ligand **9a** was dissolved in the assay buffer and 5 μL of this solution (final assay concentration: 50 nM) was pipetted to a 384-well plate, followed by the addition of 5 μL of varying dilutions of the competing ligand dissolved in the assay buffer. Then, 20 μL of the membrane preparation (diluted in assay buffer, 2 μg total protein/well) was added and the plates were incubated for 90 min at 37 °C. Subsequently, 5 μL of a furimazine solution (final assay dilution: 1:5000 in the assay buffer) was added to each well (final assay volume: 35 μL). Plates were read on a CLARIOstar microplate reader using 620/10 and 475/30 nm emission filters after 5 min of incubation in the dark. To determine the inhibition constants (*K*_i_) of the nonlabeled ligands, data were analyzed using the one-site-fit *K*_i_ equation in PRISM9.5 (GraphPad). For compounds that showed more than 50% competition at the highest concentration tested, we manually set a constraint for the curve fitting to approach the value detected for nonspecific binding (0% specific BRET). For compounds that showed less than 50% competition at the highest competitor concentration tested, only the values for percentual inhibition of tracer binding at a given concentration are provided.

#### Membrane-Based NanoBRET Association Kinetic Assay

5 μL of a solution of the fluorescent ligand (**9a**) diluted to varying concentrations (final assay concentrations: 10–100 nM) in the assay buffer and 5 μL of the assay buffer were transferred to a 384-well plate. For the determination of nonspecific binding, we added a solution of a nonlabeled competitor dissolved in the assay buffer (**3**, final assay concentration: 10 μM) instead. After the addition of 5 μL of a furimazine solution (final assay dilution: 1:350 in assay buffer), plates were incubated for 3 min in the dark at ambient temperature. Subsequently, 20 μL of the membrane preparation (2 μg total protein/well) was added (final assay volume: 35 μL). BRET ratios were measured with a CLARIOstar microplate reader using 620/10 and 475/30 nm emission filters over time at ambient temperature or 37 °C. The obtained data were analyzed using the association kinetics (one ligand concentration) algorithm in PRISM9.5 (GraphPad) to determine association kinetics using a predetermined *k*_off_ as a constraint.

#### Membrane-Based NanoBRET Dissociation Kinetic Assay

5 μL of a solution of the fluorescent ligand (**9a**) diluted to varying concentrations (final assay concentrations: 10–100 nM) in the assay buffer and 5 μL of the assay buffer were transferred to a 384-well plate. In order to determine nonspecific binding, we added a solution of a nonlabeled competitor dissolved in the assay buffer (**3**, final assay concentration: 10 μM) instead of the 5 μL of the assay buffer. Then, 20 μL of the membrane preparation (2 μg total protein/well) was added, and plates were incubated for 4 h at ambient temperature in the dark. Subsequently, 5 μL of a vivazine solution (final assay dilution: 1:350 in assay buffer) was added. Plates were incubated for further 60 min in the dark at ambient temperature. Thereafter, 1 μL of a solution of a non-unlabeled competitor dissolved in the assay buffer (**3**, final assay concentration: 10 μM) was added. For control experiments, we added 1 μL of the assay buffer instead of the competitor solution. BRET ratios were measured with a CLARIOstar microplate reader using 620/10 and 475/30 nm emission filters over time at ambient temperature or 37 °C. Specific BRET ratios were calculated as a difference of total and nonspecific binding. The obtained data were analyzed using the dissociation—one phase exponential decay algorithm in PRISM9.5 (GraphPad) to determine dissociation kinetics.

#### Membrane-Based NanoBRET Competition Kinetic Assay

2.5 μL of a solution of the fluorescent ligand (**9a**) diluted in the assay buffer (final assay concentration: 50 nM) and 2.5 μL of a solution of the competing nonlabeled ligands (final assay concentrations: 075 nM) or **3** for determining nonspecific binding (final assay concentration: 25 μM) in the assay buffer were transferred to a 384-well plate. Next, 5 μL of furimazine (final assay dilution: 1:480 in assay buffer) was added, the plate was centrifuged, and incubated for 5 min in the dark at ambient temperature. Thereafter, 20 μL of the membrane preparation (2 μg total protein/well) was added and BRET ratios were measured with a CLARIOstar microplate reader using 620/10 and 475/30 nm emission filters over time. Specific BRET ratios were calculated as a difference of total and nonspecific binding. The obtained data were analyzed using the kinetics of the competitive binding algorithm in PRISM9.5 (GraphPad) to determine competition binding kinetics.

#### Live Cell NanoBRET

HEK293T cells were transfected with the plasmid (5.5 μg) for CXCR2_Nluc using polyethylenimine (PEI) as transfection reagent (PEI/DNA ratio 3:1). After 24 h at 37 °C and 5% CO_2_, the cells were detached with DMEM and transferred to a white F-bottom 384-well plate (10,000 cells/well), which was coated with 5 μL/well poly-d-lysine (0.1 mg/mL, dissolved in water), and incubated for further 24 h at 37 °C and 5% CO_2_. Subsequently, the cells were washed with phosphate-buffered saline (Gibco DPBS, with CaCl_2_ and MgCl_2_). The assay medium (Gibco DMEM/F12, 15 mM HEPES, no phenol red supplemented with 5% FBS) was added and the cells were incubated at 37 °C for 30 min. Then, 5 μL of a solution containing the fluorescent ligand (**9a**), diluted in the assay medium at varying concentrations, was added in the case of saturation binding experiments. To determine nonspecific binding, 5 μL of a solution of **3** dissolved in the assay medium (final assay concentration: 10 μM) were added. For competition binding experiments, 5 μL of a solution of the fluorescent ligand (**9a**) diluted in the assay medium (final assay concentration: 10 μM) and 5 μL of a solution of the potential competitor (diluted from 10 mM DMSO-stock solutions with assay medium) at varying concentrations were added to the corresponding wells.

After 120 min of incubation at 37 °C, 5 μL of a furimazine solution (final assay dilution: 1:2500, diluted with assay medium) was added. Subsequent to a further incubation of 5 min in the dark at 37 °C, BRET ratios were measured with a CLARIOstar microplate reader using 620/10 and 475/30 nm emission filters. Total, nonspecific and specific binding, which was calculated as a difference of total and nonspecific binding, were analyzed using the algorithms for one-site saturation binding in PRISM9.5 (GraphPad). To determine the inhibition constants (*K*_i_) of the potential competitors, data were normalized to total and nonspecific binding and analyzed using the one-site-fit *K*_i_ equation in PRISM9.5 (GraphPad).

#### Live Cell NanoBRET Association Kinetic Assay

HEK293T cells were transfected with the plasmid (5.5 μg) for CXCR2_Nluc using polyethylenimine (PEI) as the transfection reagent (PEI/DNA ratio 3:1). After 24 h at 37 °C and 5% CO_2_, the cells were detached with DMEM and transferred to a white F-bottom 384-well plate (10,000 cells/well), which was coated with 5 μL/well poly-d-lysine (0.1 mg/mL, dissolved in water), and incubated for further 24 h at 37 °C and 5% CO_2_. Subsequently, the cells were washed with phosphate-buffered saline (Gibco DPBS, with CaCl_2_ and MgCl_2_). 15 μL of the assay medium (Gibco DMEM/F12, 15 mM HEPES, no phenol red supplemented with 5% FBS) and 5 μL of vivazine (final assay dilution: 1:300 in the assay buffer) were added and the cells were incubated at 37 °C for 90 min. Then, 5 μL of the fluorescent ligand (**9a**), diluted to varying concentrations (final assay concentrations: 10–0.5 nM in the assay medium) was transferred to a 384-well plate. For the determination of nonspecific binding, we added a solution of the fluorescent ligand (**9a**) and a nonlabeled competitor dissolved in the assay medium (**3**, final assay concentration: 10 μM) instead. BRET ratios were measured with a CLARIOstar microplate reader using 620/10 and 475/30 nm emission filters over time. The obtained data were analyzed using the association kinetics (two or more conc. of hot ligand) algorithm in PRISM9.5 (GraphPad) to determine the association kinetics.

#### Live Cell NanoBRET Dissociation Kinetic Assay

After running the association assay in cells for at least 133 min, 1 μL of an unlabeled competitor dissolved in the assay buffer (**3**, final assay concentration: 10 μM) was added. For control experiments, we added 1 μL of the assay medium instead of the competitor solution. BRET ratios were measured with a CLARIOstar microplate reader using 620/10 and 475/30 nm emission filters over time. Specific BRET ratios were calculated as a difference of total and nonspecific binding. The obtained data were analyzed using the dissociation one phase exponential decay algorithm in PRISM9.5 (GraphPad) to determine the dissociation kinetics.

### Fluorescence Microscopy

For live cell microscopy, HEK293 cells stably expressing CXCR2 were seeded onto poly-d-lysine (PDL)-coated 8-well μ-slides (Ibidi) at a density of 1.5 × 10^5^ cells per well and cultured overnight at 37 °C and 5% CO_2_. On the next day, cells were incubated with navarixin (**2**), cmpd24 (**3**) (final concentration 10 μM), or vehicle solution (OptiMEM reduced serum medium (Gibco) with 0.1% DMSO) for 60 min at 37 °C. Afterward, the fluorescent CXCR2 ligand ((**9a**) final concentration 500 nM) was added for 60 min at 37 °C, and the cells were washed once with PBS before imaging. For immunostaining, the cells were first fixed with 4% paraformaldehyde solution, washed with PBS, and then blocked for 1 h with 10% goat serum and 1% fatty acid-free bovine serum albumin (BSA) in PBS at 37 °C. As primary antibody, anti-CXCR2 mouse IgG (R&D Systems, catalog # 48311, 1:100 in blocking solution) was added, and the cells were incubated for 1 h at 37 °C. After three washes with PBS, goat anti-mouse IgG (H+L) conjugated to FITC (Sigma-Aldrich, catalog # AP124F, 1:500 in blocking solution) was added for 1 h at 37 °C. The cells were again washed three times with PBS and counterstained with 4′,6-diamidin-2-phenylindole (DAPI) solution (0.1 μg/mL) for 15 min in the dark at room temperature, followed by three washes with PBS. On the next day, the cells were incubated with navarixin (**2**), cmpd24 (**3**) (final concentration 10 μM), or vehicle solution (OptiMEM reduced serum medium (Gibco) with 0.1% DMSO) for 60 min at 37 °C. Afterward, the fluorescent CXCR2 ligand ((**9a**) final concentration 500 nM) was added for 30 min at 37 °C and the cells were imaged. Microscopy was carried out on an AxioObserver.Z1 microscope (Carl Zeiss, Jena, Germany) equipped with an ApoTome imaging system and a Heating unit XL S using a Plan-Apochromat ×63/1.40 Oil objective (filter set 43 (red), 38 (green), 49 (blue)). Image processing and line scan analysis were performed with Zen blue Imaging software. The fluorescence intensity values were normalized to vehicle control.

### Molecular Docking

Molecular ligand structures were geometry-optimized as neutral molecules by Avogadro (Version 1.2.0)^46^ using the universal force field (UFF) and the Steepest Decent Algorithm until convergence (Δ*E* = 0). The inactive X-ray crystal structure of CXCR2 cocrystallized with 00767013 (**1**, PDB ID: 6LFL)^4^ was used as the receptor. The compounds were docked by AutoDock Vina 1.1.2.^47,48^ An exhaustiveness value of 8 and a search space of 22 × 22 × 22 Å^3^ were applied around the unliganded intracellular allosteric binding site of CXCR2. Twenty docking poses were generated for each ligand and inspected manually and according to the docking score.

### PAINS Analysis

For the identification of potential pan-assay interference compounds (PAINS), all novel compounds presented in this study were reviewed using http://zinc15.docking.org/patterns/home/. None of the novel final compounds (**8**, **9a**–**c**) and intermediates (**11**–**13**, **17**–**18**) were flagged as PAINS.

## Supporting Information Available

Figures S1–S8, Scheme S1, Tables S1 and S2, NMR spectra, and HPLC chromatograms (PDF)
Docking poses of CXCR2 in complex with **1**, **3**, or **8** (PDB)
Molecular formula strings (CSV)

## Supplementary Material

jm3c00769_si_001.pdf

jm3c00769_si_002.pdb

## Abbreviations Used

BRET: bioluminescence resonance energy transfer
CCR2: CC chemokine receptor 2
CCR7: CC chemokine receptor 7
CCR9: CC chemokine receptor 9
COPD: chronic obstructive pulmonary disease
CuAAC: Cu(I)-catalyzed azide–alkyne cycloaddition
CXCR1: CXC chemokine receptor 1
CXCR2: CXC chemokine receptor 2
DIPEA: *N*,*N*-diisopropylethylamine
DMEM: Dulbecco’s Modified Eagle Medium
GPCRs: G protein-coupled receptors
HA: hemagglutinin
HEK: human embryonic kidney
HRP: horseradish peroxidase
HTS: high-throughput screening
IABS: intracellular allosteric binding site
IgG: immunoglobulin G
Nluc: nanoluciferase
PAINS: pan-assay interference compounds
PDB: protein data bank
PEG: poly(ethylene glycol)
PMN: polymorphonuclear
PyBroP: bromo-tris-pyrrolidino-phosphonium hexafluorophosphate
TAMRA: tetramethylrhodamine
TBTA: tris(benzyltriazolylmethyl)-amine
*t*_r_: residence time
β_2_AR: β-2 adrenergic receptor

## Footnotes

a) The quaternary ^13^C NMR (DEPTQ) signals for 3*H*-xanthene C-4a,8a,9,9a,10a and F_3_C–COO^–^ could not be detected.

b) ^13^C NMR (DEPTQ) signals for 3*H*-xanthene C-2,3,4a,6,7,8a,9,9a,10a, carbamoyl benzoate C-1,2,3,4,6, −COOH, and F_3_C–COO^–^ could not be detected.

c) ^13^C NMR (DEPTQ) signals for 3*H*-xanthene C-2,3,4a,6,7,8a,9,9a,10a, carbamoyl benzoate C-1,3,4,5,6, −COOH, and F_3_C–COO^–^ could not be detected.

## References

[ref1] Santos R.; Ursu O.; Gaulton A.; Bento A. P.; Donadi R. S.; Bologa C. G.; Karlsson A.; Al-Lazikani B.; Hersey A.; Oprea T. I.; Overington J. P. A comprehensive map of molecular drug targets. Nat. Rev. Drug Discovery 2017, 16, 19–34. 10.1038/nrd.2016.230. 27910877 PMC6314433

[ref2] Felder C. C. GPCR drug discovery-moving beyond the orthosteric to the allosteric domain. In Advances in Pharmacology; Elsevier, 2019; Vol. 86, pp 1–20. 31378249 10.1016/bs.apha.2019.04.002

[ref3] Zheng Y.; Qin L.; Zacarias N. V.; de Vries H.; Han G. W.; Gustavsson M.; Dabros M.; Zhao C.; Cherney R. J.; Carter P.; Stamos D.; Abagyan R.; Cherezov V.; Stevens R. C.; AP I. J.; Heitman L. H.; Tebben A.; Kufareva I.; Handel T. M. Structure of CC chemokine receptor 2 with orthosteric and allosteric antagonists. Nature 2016, 540, 458–461. 10.1038/nature20605. 27926736 PMC5159191

[ref4] Liu K.; Wu L.; Yuan S.; Wu M.; Xu Y.; Sun Q.; Li S.; Zhao S.; Hua T.; Liu Z. J. Structural basis of CXC chemokine receptor 2 activation and signalling. Nature 2020, 585, 135–140. 10.1038/s41586-020-2492-5. 32610344

[ref5] Jaeger K.; Bruenle S.; Weinert T.; Guba W.; Muehle J.; Miyazaki T.; Weber M.; Furrer A.; Haenggi N.; Tetaz T.; Huang C. Y.; Mattle D.; Vonach J. M.; Gast A.; Kuglstatter A.; Rudolph M. G.; Nogly P.; Benz J.; Dawson R. J. P.; Standfuss J. Structural basis for allosteric ligand recognition in the human CC chemokine receptor 7. Cell 2019, 178, 1222–1230. 10.1016/j.cell.2019.07.028. 31442409 PMC6709783

[ref6] Oswald C.; Rappas M.; Kean J.; Dore A. S.; Errey J. C.; Bennett K.; Deflorian F.; Christopher J. A.; Jazayeri A.; Mason J. S.; Congreve M.; Cooke R. M.; Marshall F. H. Intracellular allosteric antagonism of the CCR9 receptor. Nature 2016, 540, 462–465. 10.1038/nature20606. 27926729

[ref7] Liu X.; Ahn S.; Kahsai A. W.; Meng K. C.; Latorraca N. R.; Pani B.; Venkatakrishnan A. J.; Masoudi A.; Weis W. I.; Dror R. O.; Chen X.; Lefkowitz R. J.; Kobilka B. K. Mechanism of intracellular allosteric beta2AR antagonist revealed by X-ray crystal structure. Nature 2017, 548, 480–484. 10.1038/nature23652. 28813418 PMC5818265

[ref8] Ortiz Zacarías N. V.; Lenselink E. B.; IJzerman A. P.; Handel T. M.; Heitman L. H. Intracellular receptor modulation: novel approach to target GPCRs. Trends Pharmacol. Sci. 2018, 39, 547–559. 10.1016/j.tips.2018.03.002. 29653834 PMC7048003

[ref9] Zweemer A. J. M.; Nederpelt I.; Vrieling H.; Hafith S.; Doornbos M. L.; de Vries H.; Abt J.; Gross R.; Stamos D.; Saunders J.; Smit M. J.; Ijzerman A. P.; Heitman L. H. Multiple binding sites for small-molecule antagonists at the CC chemokine receptor 2. Mol. Pharmacol. 2013, 84, 551–561. 10.1124/mol.113.086850. 23877010

[ref10] Zweemer A. J. M.; Bunnik J.; Veenhuizen M.; Miraglia F.; Lenselink E. B.; Vilums M.; de Vries H.; Gibert A.; Thiele S.; Rosenkilde M. M.; IJzerman A. P.; Heitman L. H. Discovery and mapping of an intracellular antagonist binding site at the chemokine receptor CCR2. Mol. Pharmacol. 2014, 86, 358–368. 10.1124/mol.114.093328. 25024169

[ref11] Solari R.; Pease J. E.; Begg M. “Chemokine receptors as therapeutic targets: why aren’t there more drugs?”. Eur. J. Pharmacol. 2015, 746, 363–367. 10.1016/j.ejphar.2014.06.060. 25016087

[ref12] Kraus S.; Kolman T.; Yeung A.; Deming D. Chemokine receptor antagonists: role in oncology. Curr. Oncol. Rep. 2021, 23, 131 10.1007/s11912-021-01117-8. 34480662

[ref13] Billen M.; Schols D.; Verwilst P. Targeting chemokine receptors from the inside-out: discovery and development of small-molecule intracellular antagonists. Chem. Commun. 2022, 58, 4132–4148. 10.1039/D1CC07080K. 35274633

[ref14] Dwyer M. P.; Yu Y.; Chao J.; Aki C.; Chao J.; Biju P.; Girijavallabhan V.; Rindgen D.; Bond R.; Mayer-Ezel R.; Jakway J.; Hipkin R. W.; Fossetta J.; Gonsiorek W.; Bian H.; Fan X.; Terminelli C.; Fine J.; Lundell D.; Merritt J. R.; Rokosz L. L.; Kaiser B.; Li G.; Wang W.; Stauffer T.; Ozgur L.; Baldwin J.; Taveras A. G. Discovery of 2-hydroxy-N,N-dimethyl-3-{2-[[(R)-1-(5-methylfuran-2-yl)propyl]amino]-3,4-dioxocyclobut-1-enylamino}benzamide (SCH 527123): a potent, orally bioavailable CXCR2/CXCR1 receptor antagonist. J. Med. Chem. 2006, 49, 7603–7606. 10.1021/jm0609622. 17181143

[ref15] White J. R.; Lee J. M.; Young P. R.; Hertzberg R. P.; Jurewicz A. J.; Chaikin M. A.; Widdowson K.; Foley J. J.; Martin L. D.; Griswold D. E.; Sarau H. M. Identification of a potent, selective non-peptide CXCR2 antagonist that inhibits interleukin-8-induced neutrophil migration. J. Biol. Chem. 1998, 273, 10095–10098. 10.1074/jbc.273.17.10095. 9553055

[ref16] Sarau H. M.; Widdowson K. L.; Palovich M. R.; White J. R.; Underwood D. C.; Griswold D. E. Interleukin-8 Receptor (CXCR2) Antagonists. In New Drugs for Asthma, Allergy and COPD; KARGER, 2001; Vol. 31, pp 293–296.

[ref17] Bachelerie F.; Ben-Baruch A.; Burkhardt A. M.; Combadiere C.; Farber J. M.; Graham G. J.; Horuk R.; Sparre-Ulrich A. H.; Locati M.; Luster A. D.; Mantovani A.; Matsushima K.; Murphy P. M.; Nibbs R.; Nomiyama H.; Power C. A.; Proudfoot A. E. I.; Rosenkilde M. M.; Rot A.; Sozzani S.; Thelen M.; Yoshie O.; Zlotnik A. International Union of Pharmacology. LXXXIX. Update on the extended family of chemokine receptors and introducing a new nomenclature for atypical chemokine receptors. Pharmacol. Rev. 2014, 66, 1–79. 10.1124/pr.113.007724. 24218476 PMC3880466

[ref18] Miller B. E.; Mistry S.; Smart K.; Connolly P.; Carpenter D. C.; Cooray H.; Bloomer J. C.; Tal-Singer R.; Lazaar A. L. The pharmacokinetics and pharmacodynamics of danirixin (GSK1325756) - a selective CXCR2 antagonist - in healthy adult subjects. BMC Pharmacol. Toxicol. 2015, 16, 18 10.1186/s40360-015-0017-x. 26092545 PMC4475328

[ref19] Salchow K.; Bond M. E.; Evans S. C.; Press N. J.; Charlton S. J.; Hunt P. A.; Bradley M. E. A common intracellular allosteric binding site for antagonists of the CXCR2 receptor. Br. J. Pharmacol. 2010, 159, 1429–1439. 10.1111/j.1476-5381.2009.00623.x. 20233217 PMC2850400

[ref20] Nicholls D. J.; Tomkinson N. P.; Wiley K. E.; Brammall A.; Bowers L.; Grahames C.; Gaw A.; Meghani P.; Shelton P.; Wright T. J.; Mallinder P. R. Identification of a putative intracellular allosteric antagonist binding-site in the CXC chemokine receptors 1 and 2. Mol. Pharmacol. 2008, 74, 1193–1202. 10.1124/mol.107.044610. 18676678

[ref21] www.clinicaltrials.gov.

[ref22] Toy L.; Huber M. E.; Schmidt M. F.; Weikert D.; Schiedel M. Fluorescent ligands targeting the intracellular allosteric binding site of the chemokine receptor CCR2. ACS Chem. Biol. 2022, 17, 2142–2152. 10.1021/acschembio.2c00263. 35838163

[ref23] Huber M. E.; Toy L.; Schmidt M. F.; Vogt H.; Budzinski J.; Wiefhoff M. F. J.; Merten N.; Kostenis E.; Weikert D.; Schiedel M. A chemical biology toolbox targeting the intracellular binding site of CCR9: fluorescent ligands, new drug leads and PROTACs. Angew. Chem. Int. Ed. 2022, 61, e202116782 10.1002/anie.202116782. PMC9306553 34936714

[ref24] Huisgen R. 1,3-Dipolar cycloadditions. Proc. Chem. Soc. 1961, 357–396. 10.1039/PS9610000357.

[ref25] Rostovtsev V. V.; Green L. G.; Fokin V. V.; Sharpless K. B. A stepwise huisgen cycloaddition process: copper(I)-catalyzed regioselective ″ligation″ of azides and terminal alkynes. Angew. Chem. Int. Ed. 2002, 41, 2596–2599. 10.1002/1521-3773(20020715)41:14<2596::AID-ANIE2596>3.0.CO;2-4. 12203546

[ref26] Tornøe C. W.; Christensen C.; Meldal M. Peptidotriazoles on solid phase: [1,2,3]-triazoles by regiospecific copper(i)-catalyzed 1,3-dipolar cycloadditions of terminal alkynes to azides. J. Org. Chem. 2002, 67, 3057–3064. 10.1021/jo011148j. 11975567

[ref27] Aki C. J.; Baldwin J. J.; Bond R. W.; Chao J.; Chao J.; Dwyer M.; Ferreira J. A.; Kaiser B.; Li G.; Merritt J. R.; Nelson K. H.; Rokosz L. L.; Taveras A. G.; Yu Y. 3,4-Di-substituted cyclobutene-1,2-diones as cxc-chemokine receptor ligands. AU2010212484A1, 2010.

[ref28] Taveras A. G.; Chao J.; Biju P. J.; Yu Y.; Aki C. J.; Merritt R. J.; Li G.; Baldwin J. J.; Lai G.; Wu M.; Hecker E. A. Thiadiazoledioxides and thiadiazoleoxides as CXC- and CC-chemokine receptor ligands. US7691856B2, 2010.

[ref29] Rosier N.; Gratz L.; Schihada H.; Moller J.; Isbilir A.; Humphrys L. J.; Nagl M.; Seibel U.; Lohse M. J.; Pockes S. A versatile sub-nanomolar fluorescent ligand enables NanoBRET binding studies and single-molecule microscopy at the histamine H(3) receptor. J. Med. Chem. 2021, 64, 11695–11708. 10.1021/acs.jmedchem.1c01089. 34309390

[ref30] Sakyiamah M. M.; Nomura W.; Kobayakawa T.; Tamamura H. Development of a NanoBRET-based sensitive screening method for CXCR4 ligands. Bioconjugate Chem. 2019, 30, 1442–1450. 10.1021/acs.bioconjchem.9b00182. 30973227

[ref31] Ellman J. A. Applications of tert-butanesulfinamide in the asymmetric synthesis of amines. Pure Appl. Chem. 2003, 75, 39–46. 10.1351/pac200375010039.

[ref32] Robak M. T.; Herbage M. A.; Ellman J. A. Synthesis and applications of tert-butanesulfinamide. Chem. Rev. 2010, 110, 3600–3740. 10.1021/cr900382t. 20420386

[ref33] Hall M. P.; Unch J.; Binkowski B. F.; Valley M. P.; Butler B. L.; Wood M. G.; Otto P.; Zimmerman K.; Vidugiris G.; Machleidt T.; Robers M. B.; Benink H. A.; Eggers C. T.; Slater M. R.; Meisenheimer P. L.; Klaubert D. H.; Fan F.; Encell L. P.; Wood K. V. Engineered luciferase reporter from a deep sea shrimp utilizing a novel imidazopyrazinone substrate. ACS Chem. Biol. 2012, 7, 1848–1857. 10.1021/cb3002478. 22894855 PMC3501149

[ref34] Gonsiorek W.; Fan X. D.; Hesk D.; Fossetta J.; Qiu H. C.; Jakway J.; Billah M.; Dwyer M.; Chao J. H.; Deno G.; Taveras A.; Lundell D. J.; Hipkin R. W. Pharmacological characterization of sch527123, a potent allosteric CXCR1/CXCR2 antagonist. J. Pharmacol. Exp. Ther. 2007, 322, 477–485. 10.1124/jpet.106.118927. 17496166

[ref35] Ortiz Zacarías N. V.; van Veldhoven J. P. D.; Portner L.; van Spronsen E.; Ullo S.; Veenhuizen M.; van der Velden W. J. C.; Zweemer A. J. M.; Kreekel R. M.; Oenema K.; Lenselink E. B.; Heitman L. H.; AP I. J. Pyrrolone derivatives as intracellular allosteric modulators for chemokine receptors: selective and dual-targeting inhibitors of CC chemokine receptors 1 and 2. J. Med. Chem. 2018, 61, 9146–9161. 10.1021/acs.jmedchem.8b00605. 30256641 PMC6328288

[ref36] Peace S.; Philp J.; Brooks C.; Piercy V.; Moores K.; Smethurst C.; Watson S.; Gaines S.; Zippoli M.; Mookherjee C.; Ife R. Identification of a sulfonamide series of CCR2 antagonists. Bioorg. Med. Chem. Lett. 2010, 20, 3961–3964. 10.1016/j.bmcl.2010.04.142. 20627722

[ref37] Walters M. J.; Wang Y.; Lai N.; Baumgart T.; Zhao B. N.; Dairaghi D. J.; Bekker P.; Ertl L. S.; Penfold M. E.; Jaen J. C.; Keshav S.; Wendt E.; Pennell A.; Ungashe S.; Wei Z.; Wright J. J.; Schall T. J. Characterization of CCX282-B, an orally bioavailable antagonist of the CCR9 chemokine receptor, for treatment of inflammatory bowel disease. J. Pharmacol. Exp. Ther. 2010, 335, 61–69. 10.1124/jpet.110.169714. 20660125

[ref38] Zhang J. H.; Chung T. D.; Oldenburg K. R. A simple statistical parameter for use in evaluation and validation of high throughput screening assays. SLAS Discovery 1999, 4, 67–73. 10.1177/108705719900400206. 10838414

[ref39] Bouzo-Lorenzo M.; Stoddart L. A.; Xia L. Z.; IJzerman A. P.; Heitman L. H.; Briddon S. J.; Hill S. J. A live cell NanoBRET binding assay allows the study of ligand-binding kinetics to the adenosine A(3) receptor. Purinergic Signalling 2019, 15, 139–153. 10.1007/s11302-019-09650-9. 30919204 PMC6635573

[ref40] Stefaniak J.; Huber K. V. M. Importance of quantifying drug-target engagement in cells. ACS Med. Chem. Lett. 2020, 11, 403–406. 10.1021/acsmedchemlett.9b00570. 32292539 PMC7153009

[ref41] Ilardi E. A.; Zakarian A. Efficient total synthesis of dysidenin, dysidin, and barbamide. Chem. Asian J. 2011, 6, 2260–2263. 10.1002/asia.201100338. 21698777

[ref42] Chen X.; Dragoli D. R.; Fan J.; Kalisiak J.; Krasinski A.; Leleti M. R.; Mali V.; McMahon J.; Singh R.; Tanaka H.; Yang J.; Yu C.; Zhang P. Preparation of isoindoline derivatives as chemokine receptor modulators useful in treatment of CXCR2- and CCR6-mediated diseases. US20170144997, 2017.

[ref43] Lowry O. H.; Rosebrough N. J.; Farr A. L.; Randall R. J. Protein measurement with the Folin phenol reagent. J. Biol. Chem. 1951, 193, 265–275. 10.1016/S0021-9258(19)52451-6. 14907713

[ref44] Gibson D. G.; Young L.; Chuang R. Y.; Venter J. C.; Hutchison C. A. 3rd; Smith H. O. Enzymatic assembly of DNA molecules up to several hundred kilobases. Nat. Methods 2009, 6, 343–345. 10.1038/nmeth.1318. 19363495

[ref45] Allikalt A.; Purkayastha N.; Flad K.; Schmidt M. F.; Tabor A.; Gmeiner P.; Hübner H.; Weikert D. Fluorescent ligands for dopamine D2/D3 receptors. Sci. Rep. 2020, 10, 21842 10.1038/s41598-020-78827-9. 33318558 PMC7736868

[ref46] Hanwell M. D.; Curtis D. E.; Lonie D. C.; Vandermeersch T.; Zurek E.; Hutchison G. R. Avogadro: an advanced semantic chemical editor, visualization, and analysis platform. J. Cheminform. 2012, 4, 17 10.1186/1758-2946-4-17. 22889332 PMC3542060

[ref47] Eberhardt J.; Santos-Martins D.; Tillack A. F.; Forli S. AutoDock Vina 1.2.0: new docking methods, expanded force field, and python bindings. J. Chem. Inf. Model. 2021, 61, 3891–3898. 10.1021/acs.jcim.1c00203. 34278794 PMC10683950

[ref48] Trott O.; Olson A. J. AutoDock Vina: improving the speed and accuracy of docking with a new scoring function, efficient optimization, and multithreading. J. Comput. Chem. 2010, 31, 455–461. 10.1002/jcc.21334. 19499576 PMC3041641

