# Supplementary material for: Fluorescent Ligands Enable Target Engagement Studies for the Intracellular Allosteric Binding Site of the Chemokine Receptor CXCR2
Source: J Med Chem. 2023 Jul 18;66(14):9916–33. doi: 10.1021/acs.jmedchem.3c00769 (PMC10388362; doi:10.1021/acs.jmedchem.3c00769)
Supplement: Supplementary file 1 — jm3c00769_si_001.pdf [file jm3c00769_si_001.pdf]

Supporting Information

Fluorescent Ligands Enable Target Engagement Studies for the Intracellular Allosteric Binding Site of the Chemokine Receptor CXCR2

Max E. Huber,[§,#] Silas Wurnig,[Δ,#] Lara Toy,[§] Corinna Weiler,[□] Nicole Merten,[□] Evi Kostenis,[□,*] Finn K. Hansen,[Δ,*] Matthias Schiedel,[§,◊,*]

[§] Department of Chemistry and Pharmacy, Medicinal Chemistry, Friedrich-Alexander-University Erlangen-Nürnberg, Nikolaus-Fiebiger-Straße 10, 91058 Erlangen, Germany

[Δ] Department of Pharmaceutical & Cell Biological Chemistry, Pharmaceutical Institute, University of Bonn, An der Immenburg 4, 53121 Bonn, Germany

[□] Molecular, Cellular and Pharmacobiology Section, Institute for Pharmaceutical Biology, University of Bonn, Nussallee 6, 53115, Bonn, Germany

[◊] Institute of Medicinal and Pharmaceutical Chemistry, Technische Universität Braunschweig, Beethovenstraße 55, 38106 Braunschweig, Germany

*E-mail: kostenis@uni-bonn.de, finn.hansen@uni-bonn.de, matthias.schiedel@tu-braunschweig.de

Table of Contents

Page Contents

Page	Contents
S3	Supplementary Figures
S11	Supplementary Schemes
S12	Supplementary Tables
S14	Supplementary NMR Spectra
S34	Supplementary HPLC Chromatograms
S39	Supplementary References

Supplementary Figures

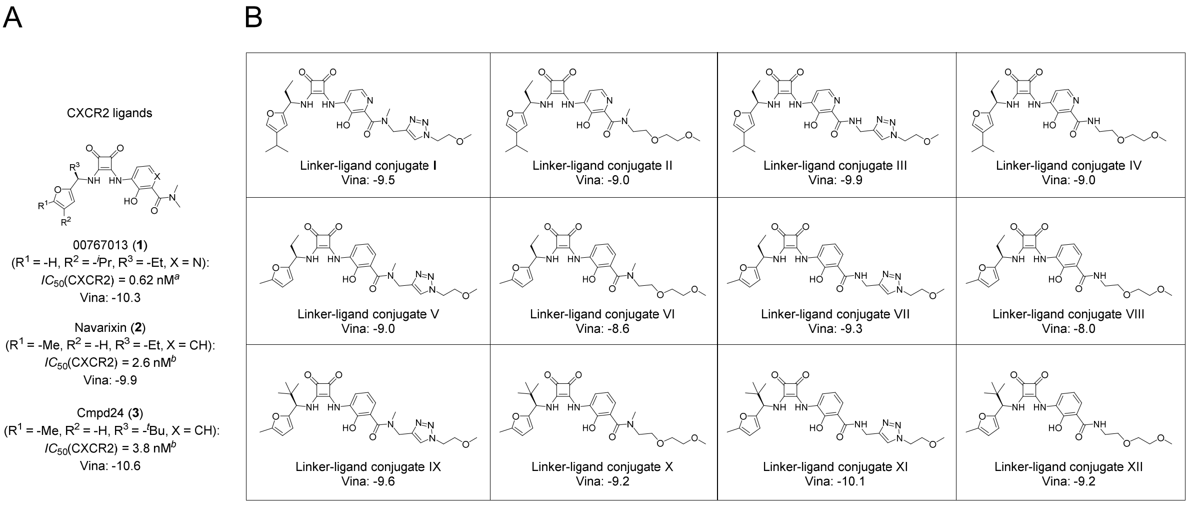

Figure S1. Identification of suitable linker fragments and linker positions for the design of fluorescently labeled CXCR2 ligands based on the scaffolds of 00767013 (**1**), navarixin (**2**) or cmpd24 (**3**). A) Chemical structures and docking scores for known intracellular allosteric CXCR2 antagonists **1**-**3**. IC_{50} values as reported by (a) Liu *et al.*[1] and (b) Dwyer *et al.*[2] B) Chemical structures and docking scores of designed CXCR2 ligand-linker conjugates docked into the crystal structure of CXCR2 with the co-crystallized allosteric inhibitor 00767013 (**1**) (PDB ID: 6LFL).[1] The design of ligand-linker conjugates was based on the reported (for **1**)[1] or predicted (for **2**, **3**) binding modes of the intracellular CXCR2 antagonists, available SAR data,[2] and the synthetic accessibility of the final fluorescent ligands. For comparability of the docking studies, the overall lengths of the linkers were restricted to 6-7 atoms.

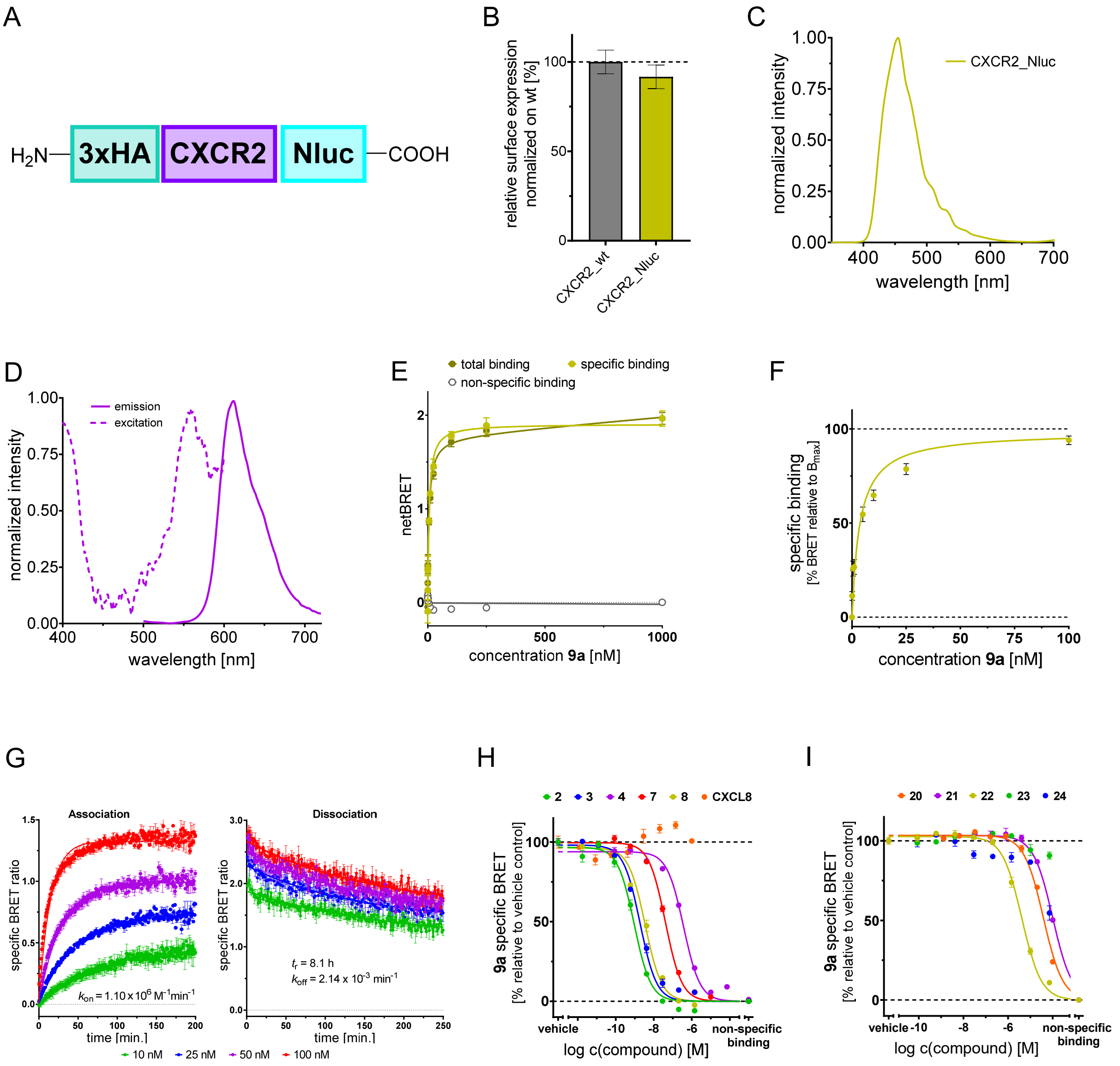

Figure S2. Development of a cell-free NanoBRET-based binding assay for CXCR2 based on the fluorescent CXCR2 ligand **9a**. A) Schematic representation of the genetic construct used in the course of assay development. B) Expression level of CXCR2_Nluc constructs detected via ELISA normalized to the expression of wild-type 3xHA-CXCR2 (CXCR2_wt). Bar diagram representing the mean values ± SEM (n = 4) with each test performed in quadruplicate. The experiment indicates that the CXCR2_Nluc construct is well-expressed in HEK293T cells. C) Emission spectra of the CXCR2_Nluc construct using membrane preparations from HEK293T cells expressing the respective Nluc-labeled 3xHA-CXCR2 fusion protein. D) Spectral properties of the fluorescent ligand **9a**. Fluorescence excitation and emission spectra of **9a** were normalized to the respective maximum signal of each sample. Excitation spectrum of the fluorescent ligand (500 µM) is shown as dashed line, emission spectrum of the fluorescent ligand (1000 µM) is represented as solid line. E) Representative binding curves (total, specific, and non-specific binding) from a single experiment, performed with **9a** and a membrane preparation from HEK293T cells expressing the CXCR2_Nluc construct. The experiment was performed in triplicate. The netBRET signal was calculated as the difference between total BRET and the signal obtained in the absence of a fluorescent ligand. F) Zoomed-in view (concentration range: 0 – 100 nM) of the specific saturation binding curve of **9a** in a cell-free NanoBRET-based experiment using CXCR2_Nluc membranes (mean ± SEM, triplicate measurement, n = 9). G) Kinetic binding studies at room temperature. Further representative association and dissociation curves with **9a** at different concentrations using CXCR2_Nluc membranes. The determined kinetic parameters from all kinetic measurements performed at room temperature are given in Table S1A-B. H) Representative competition binding curves from single experiments for known intracellular CXCR2 antagonists **2**-**4** and **7**, the ligand-linker conjugate XI (**8**), and the extracellular orthosteric agonist CXCL8, obtained with **9a** (50 nM) and CXCR2_Nluc membranes (mean ± SEM, triplicate measurement). I) Representative competition binding curves from single experiments for known intracellular chemokine receptor antagonists **20**-**24** obtained with **9a** (50 nM) and CXCR2_Nluc membranes (mean ± SEM, triplicate measurement).

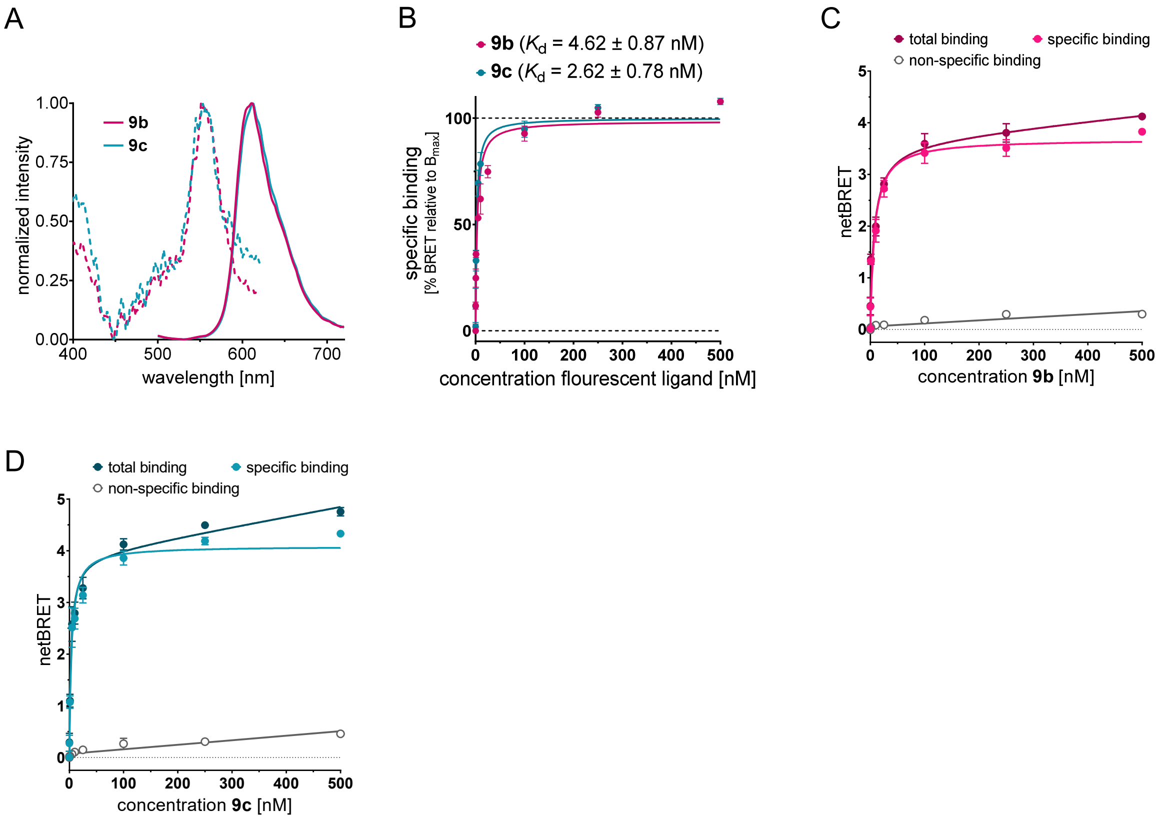

Figure S3. Characterization of fluorescent ligands **9b**-**c** as tracer molecules for cell-free CXCR2 NanoBRET-based binding assays. A) Spectral properties of the fluorescent ligands **9b**-**c**. Fluorescence excitation and emission spectra of **9b**-**c** were normalized to the respective maximum signal of each sample. Excitation spectra of the fluorescent ligands (500 µM) are shown as dashed lines, emission spectra of the fluorescent ligands (1000 µM) are represented as solid lines. B) Specific saturation binding curves of **9b**-**c** in a NanoBRET-based assay using CXCR2_Nluc membranes (mean ± SEM, triplicate measurement, n ≥ 3). C) Representative binding curves (total, specific, and non-specific binding) from a single experiment, performed with **9b** and a membrane preparation from HEK293T cells expressing the CXCR2_Nluc construct. The experiment was performed in triplicate. The netBRET signal was calculated as the difference between total BRET and the signal obtained in the absence of a fluorescent ligand. D) Representative binding curves (total, specific, and non-specific binding) from a single experiment, performed with **9c** and a membrane preparation from HEK293T cells expressing the CXCR2_Nluc construct. The experiment was performed in triplicate. The netBRET signal was calculated as the difference between total BRET and the signal obtained in the absence of a fluorescent ligand.

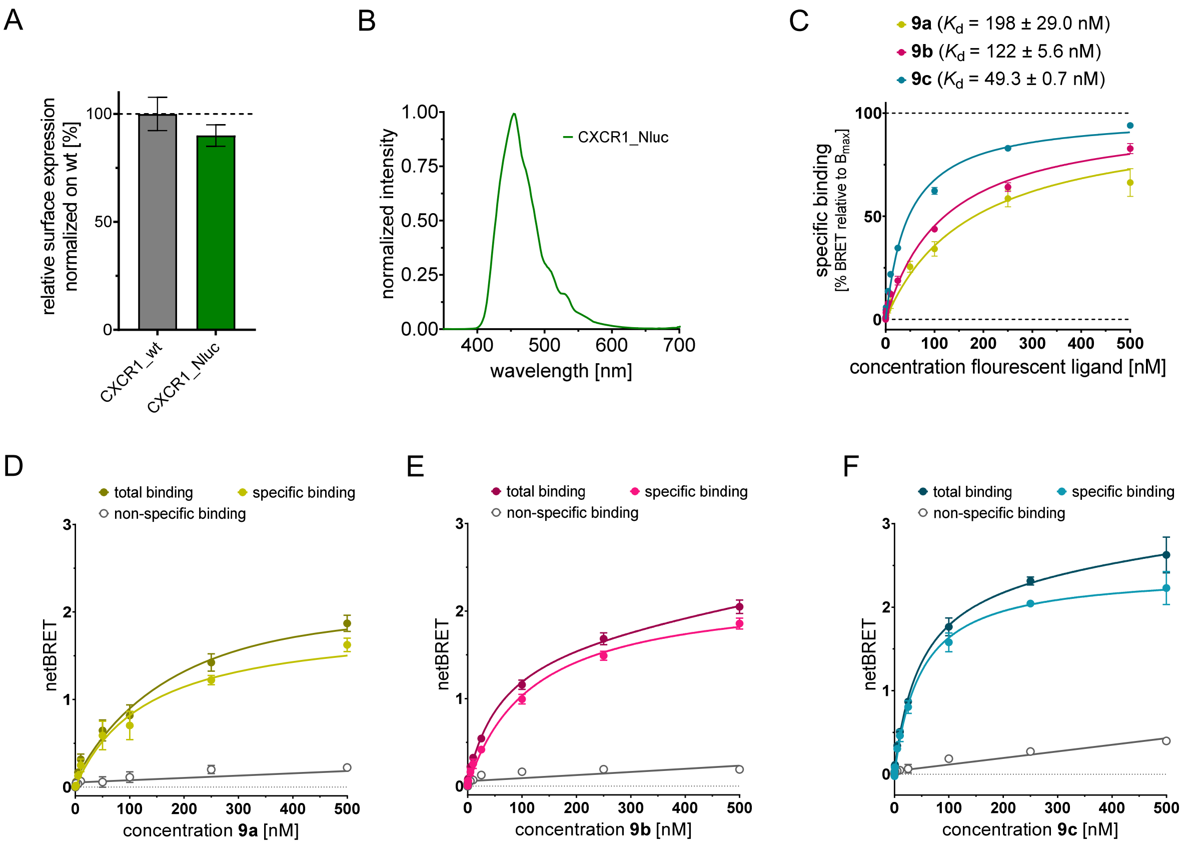

Figure S4. Selectivity studies with fluorescent CXCR2 tracers **9a-c** regarding their CXCR1 affinity. A) Expression level of the 3xHA-CXCR1-Nluc (referred to as CXCR1_Nluc) construct in HEK293T cells detected via ELISA and normalized to the expression of wild-type 3xHA CXCR1 (referred to as CXCR1_wt). Bar diagram representing the mean values ± SEM (n = 4) with each test performed in triplicate. The experiment indicates that the CXCR1_Nluc construct is well-expressed in HEK293T cells. B) Emission spectra of the CXCR1_Nluc construct using membrane preparations from HEK293T cells expressing the respective fusion protein. C) Specific saturation binding curves of **9a-c** in a NanoBRET-based assay using CXCR1_Nluc membranes (mean ± SEM, triplicate measurement, n = 3). D) Representative saturation binding curves (total, specific, and non-specific binding) for **9a** using membrane preparations from HEK293T cells expressing the CXCR1_Nluc construct. The experiment was performed in triplicate. The netBRET signal was calculated as the difference between total BRET and the signal obtained in the absence of a fluorescent ligand. E) Representative binding curves (total, specific, and non-specific binding) from a single experiment, performed with **9b** and a membrane preparation from HEK293T cells expressing the CXCR1_Nluc construct. The experiment was performed in triplicate. The netBRET signal was calculated as the difference between total BRET and the signal obtained in the absence of a fluorescent ligand. F) Representative binding curves (total, specific, and non-specific binding) from a single experiment, performed with **9c** and a membrane preparation from HEK293T cells expressing the CXCR1_Nluc construct. The experiment was performed in triplicate. The netBRET signal was calculated as the difference between total BRET and the signal obtained in the absence of a fluorescent ligand.

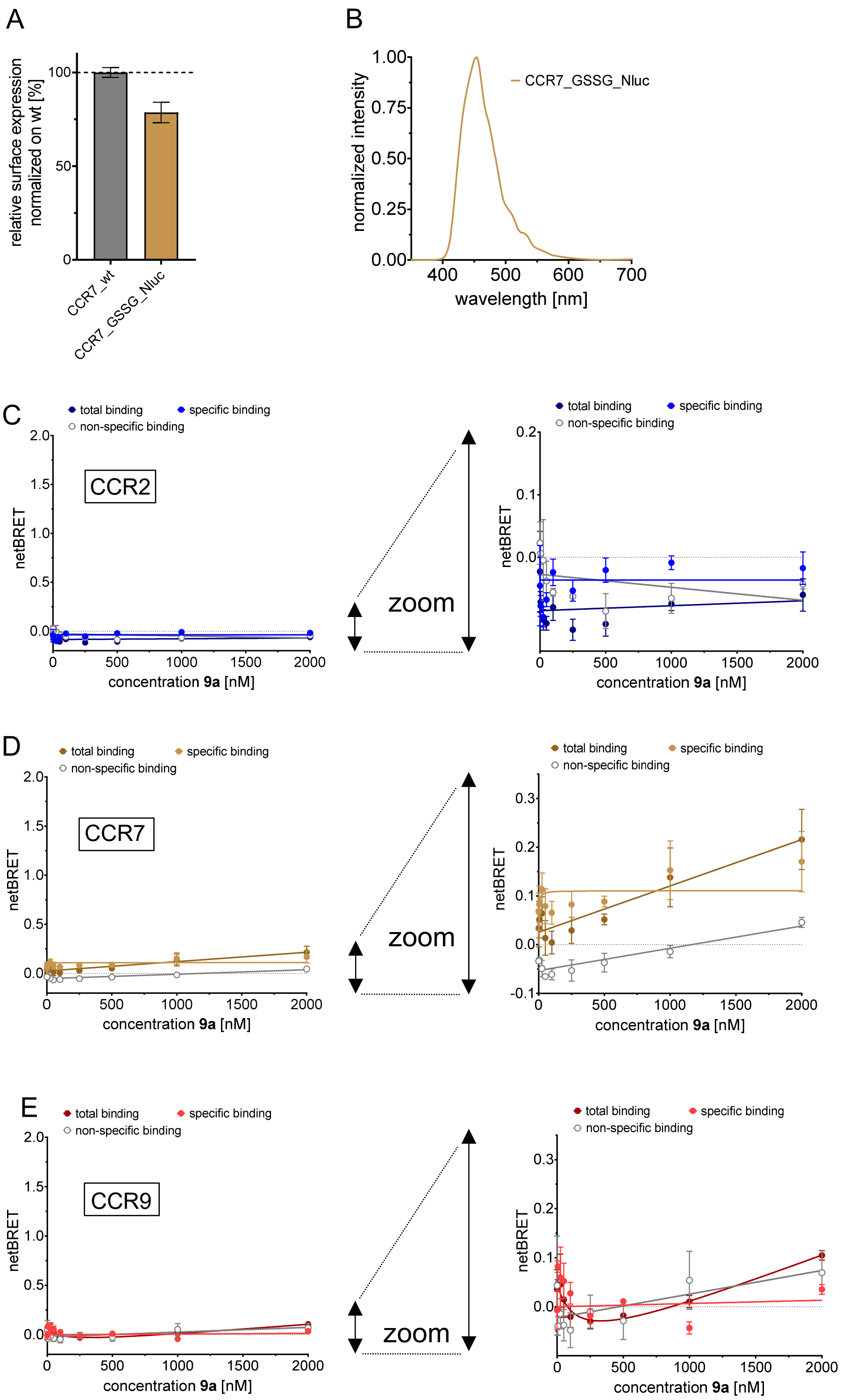

Figure S5. The fluorescent ligand **9a** selectively binds to CXCR2. A) Expression level of the 3xHA-CCR7-GSSG-Nluc (referred to as CCR7_GSSG_Nluc) construct in HEK293T cells detected via ELISA and normalized to the expression of wild-type 3xHA-CCR7 (referred to as CCR7_wt). Bar diagram representing the mean values ± SEM (n = 3) with each test performed in triplicate. The experiment indicates that the CCR7_GSSG_Nluc construct is well-expressed in HEK293T cells. B) Emission spectra of the CCR7_GSSG_Nluc using membrane preparations from HEK293T cells expressing the respective fusion protein. C) Representative saturation binding curves (total, specific, and non-specific binding) for **9a** using membrane preparations from HEK293T cells expressing the CCR2_GSSG_Nluc construct, as published by Toy *et al.*[3] The experiments were performed in triplicate (n=3). D) Representative saturation binding curves (total, specific, and non-specific binding) for **9a** using membrane preparations from HEK293T cells expressing the CCR7_GSSG_Nluc construct. The experiments were performed in triplicate (n=3). E) Representative saturation binding curves (total, specific, and non-specific binding) for **9a** using membrane preparations from HEK293T cells expressing the CCR9_Nluc construct, as published by Huber *et al.*[4] The experiments were performed in triplicate (n=3).

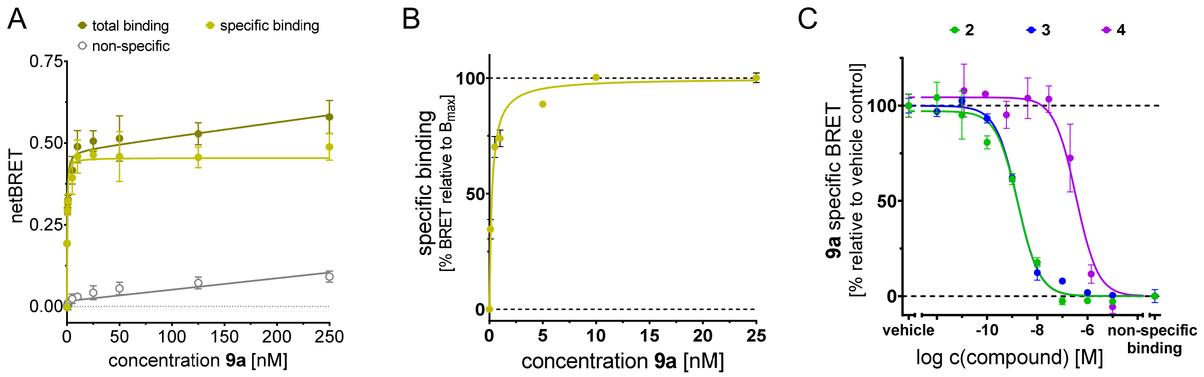

Figure S6. Establishment of a cellular NanoBRET-based CXCR2 binding assay. A) Representative saturation binding curves (total, specific, and non-specific binding) for **9a** using live HEK293T cells transiently expressing the CXCR2_Nluc construct. The experiments were performed with each condition in quadruplicate. The netBRET signal was calculated as the difference between total BRET and the signal obtained in the absence of a fluorescent ligand. B) Zoomed-in view (concentration range: 0 – 25 nM) of the specific saturation binding curve of **9a** in a cellular NanoBRET-based experiment using CXCR2_Nluc membranes (mean ± SEM, quadruplicate measurement, n = 4). C) Representative competition binding curves (mean ± SEM, quadruplicate measurement) from single experiments with known intracellular CXCR2 antagonists **2**-**4**, obtained with **9a** (50 nM) and live HEK293T cells expressing CXCR2_Nluc.

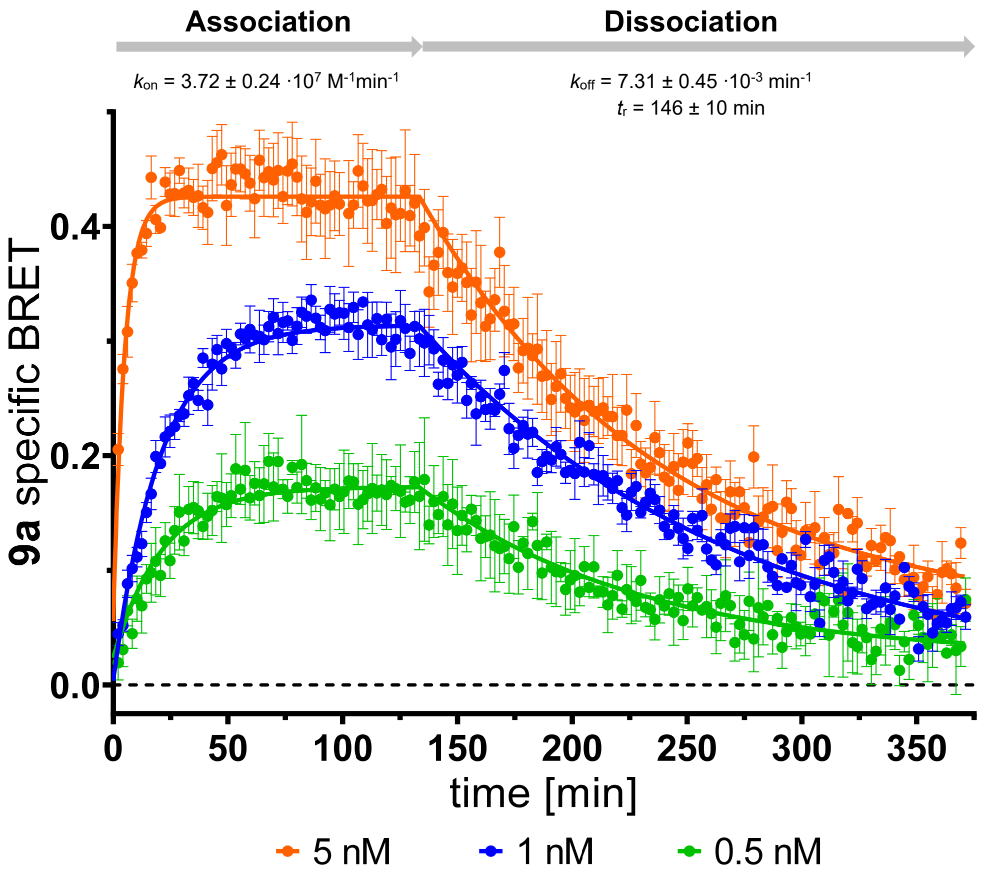

Figure S7. Further representative plots from kinetic binding studies with **9a** at different concentrations and cells expressing CXCR2_Nluc. Tests were performed at an incubation temperature of 37 °C. The determined kinetic parameters of all kinetic binding studies with live cells are listed in Table S2.

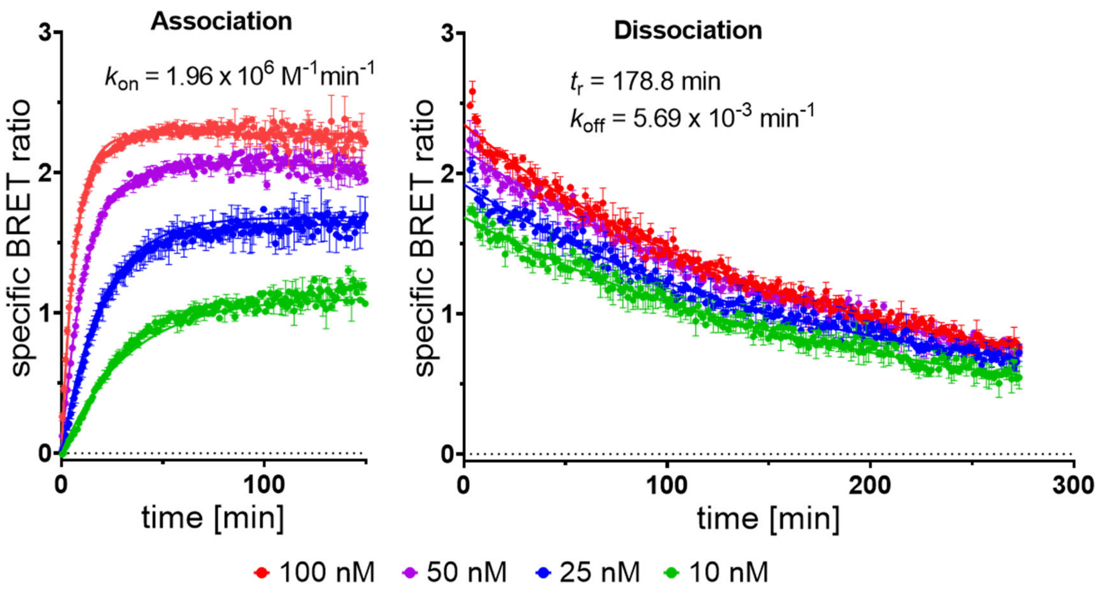

Figure S8. Kinetic binding studies with **9a** and CXCR2_Nluc membranes performed at 37 °C. Representative association and dissociation curves with **9a** at different concentrations and CXCR2_Nluc membranes. The determined kinetic parameters of all kinetic binding studies performed with CXCR2_Nluc membranes at 37 °C are listed in Table S1C-D.

Supplementary Schemes

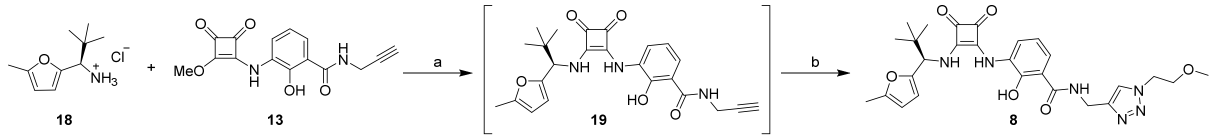

Scheme S1. Synthesis of ligand-linker conjugate XI (**8**). Reagents and conditions: a) DIPEA, MeOH, rt, 7 d, 38% yield. b) 1-Azido-2-methoxyethane, 0.1 M $CuSO_4$ solution, 0.1 M sodium ascorbate solution, TBTA, water/*tert*-BuOH/DMF mixture (1:1:1 (v/v)), rt, 2 h, 65% yield.

Supplementary Tables

Table S1. Kinetic parameters detected for the interaction between **9a** and CXCR2 by using our membrane-based NanoBRET assay and by applying the indicated conditions. A) Dissociation at room temperature. B) Association at room temperature. For the calculation of k_{on}, a pre-determined k_{off} of 0.00214 min^{-1} was used. C) Dissociation at 37 °C. D) Association at 37 °C. For the calculation of k_{on}, a pre-determined k_{off} of 0.00569 min^{-1} was used.

A

Dissociation at room temperature (membranes)

	k_{off} [min^{-1}]	t_R [min]
100 nM	0.00155	643.1
	0.00219	457.0
	0.00238	420.0
50 nM	0.00161	619.6
	0.00305	328.4
	0.00242	413.1
25 nM	0.00174	575.7
	0.00253	394.8
	0.00232	431.4
10 nM	0.00187	534.2
	0.00183	545.6
	0.00215	464.7
mean	**0.00214**	**485.6**
SEM	0.00013	28.0

B

Association at room temperature (membranes)

	k_{on} [M^{-1} min^{-1}]
100 nM	946172
	985123
	671407
50 nM	1076336
	672497
25 nM	1397434
	1235090
	806867
10 nM	2166462
	1060222
mean	**1101761**
SEM	138861

C

Dissociation at 37 °C (membranes)

	k_{off} [min^{-1}]	t_R [min]
100 nM	0.00627	159.6
	0.00726	137.7
	0.00487	205.4
50 nM	0.00560	178.6
	0.00590	169.4
	0.00531	188.3
25 nM	0.00615	162.5
	0.00658	152.0
	0.00473	211.3
10 nM	0.00530	188.6
	0.00567	176.4
	0.00464	215.5
mean	**0.00569**	**178.8**
SEM	0.00023	6.98

D

Association at 37 °C (membranes)

	k_{on} [M^{-1} min^{-1}]
100 nM	1395566
	1530507
	1187983
50 nM	1601164
	1767988
	1442177
25 nM	1774396
	2430944
	1863920
10 nM	2548680
	3161149
	2794207
mean	**1958223**
SEM	180140

Table S2. Kinetic parameters detected for the interaction between **9a** and CXCR2 by using our cell-based NanoBRET assay and by applying the indicated conditions. A) Dissociation at 37 °C. B) Association at 37 °C. For the calculation of k_{on} as well as the kinetic K_d value, a pre-determined k_{off} of 0.00731 min^{-1} was used.

A

Dissociation at 37 °C (cells)

	k_{off} [min^{-1}]	t_r [min]
10 nM	0.00452	221.0
	0.00791	126.4
	0.00898	111.4
	0.00859	116.4
5 nM	0.00476	210.2
	0.00585	170.9
	0.00720	138.9
	0.00855	117.0
1 nM	0.00533	187.6
	0.00837	119.5
	0.00621	161.1
	0.00861	116.1
0.5 nM	0.00515	194.3
	0.00892	112.1
	0.0107	93.2
	0.00727	137.6
mean	**0.00731**	**145.9**
SEM	0.00045	9.9

B

Association at 37 °C (cells)

	k_{on} [M^{-1} min^{-1}]
10 nM	38585819
	16745318
	32414479
	29506353
	21997389
	22310070
	23486653
5 nM	21115252
	28424137
	55945332
	39338866
	40592056
	57749761
	36845341
1 nM	53363551
	26448980
	63170507
	44428335
	38524942
	41590918
	30706490
0.5 nM	37734901
	37368698
	45754207
	46524384
	36205609
mean	**37187629**
SEM	2352444

Supplementary NMR spectra

NMR spectra for compounds **8**, **9a-c**, **11-13**, and **17-18** can be found on the following pages.

^{1}H NMR (400 MHz, DMSO-d_6) for compound **8**

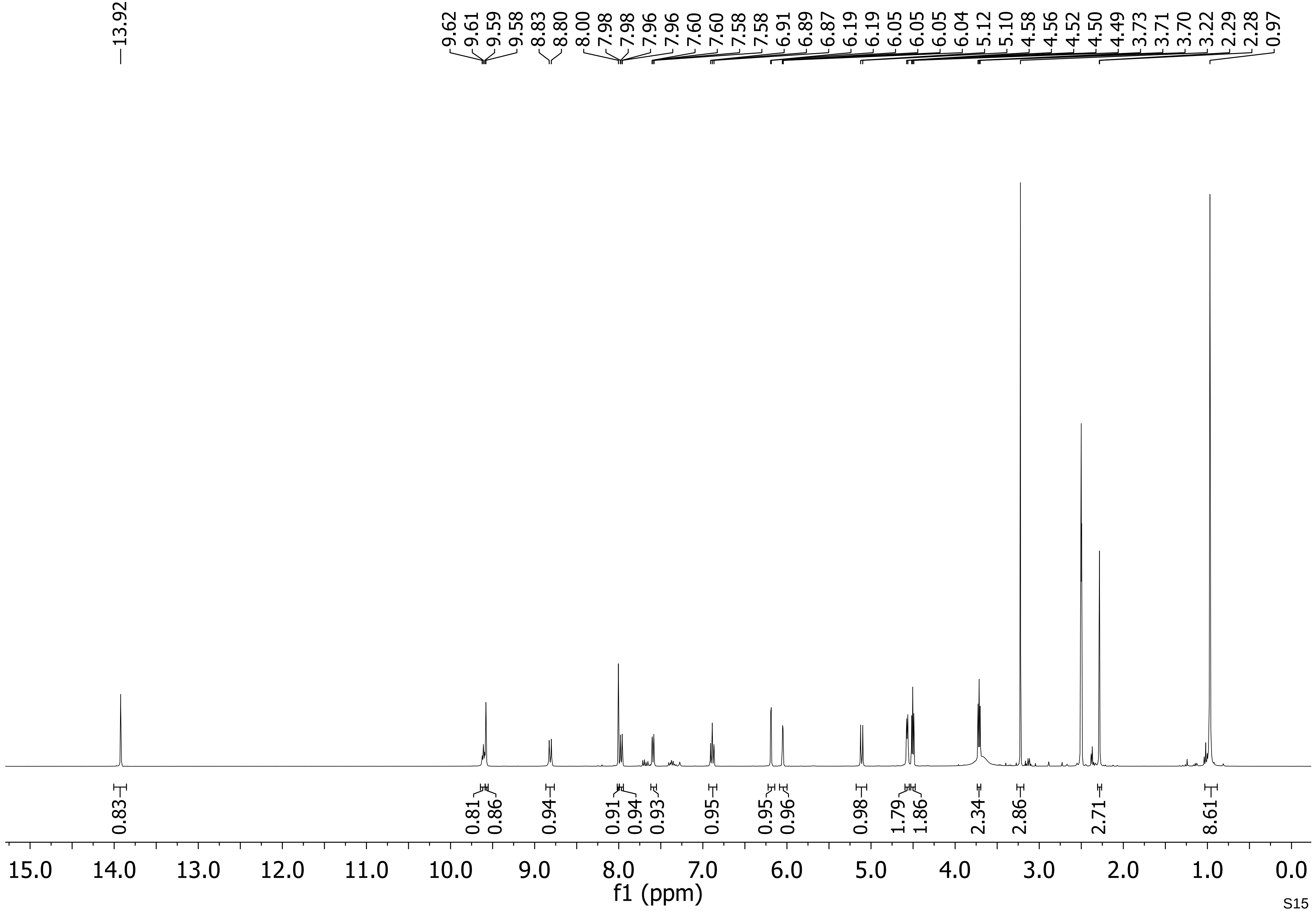

DEPTQ ^{13}C NMR (151 MHz, DMSO-d_6) for compound **8**

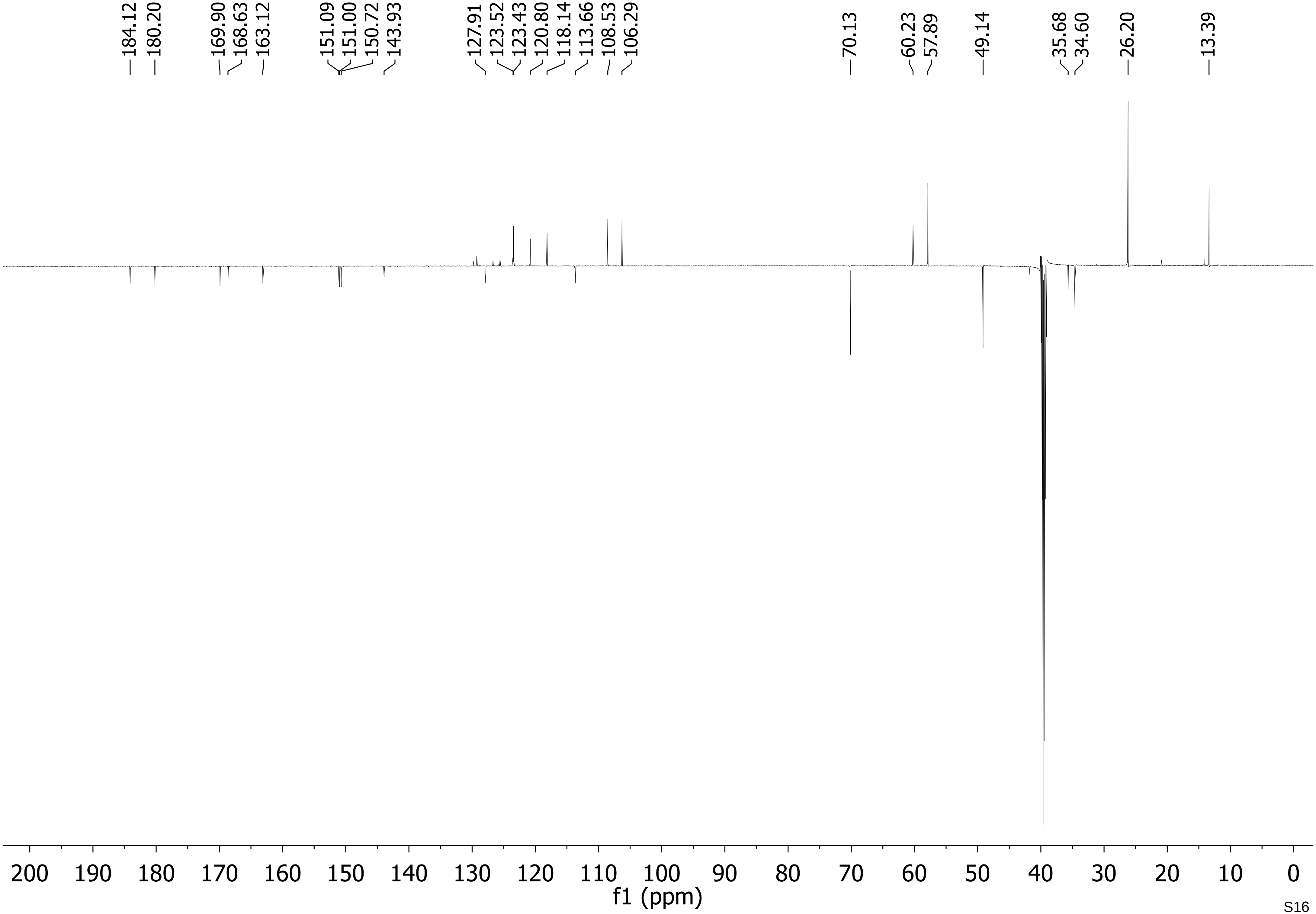

^{1}H NMR (600 MHz, DMSO-d_6) for compound **9a**

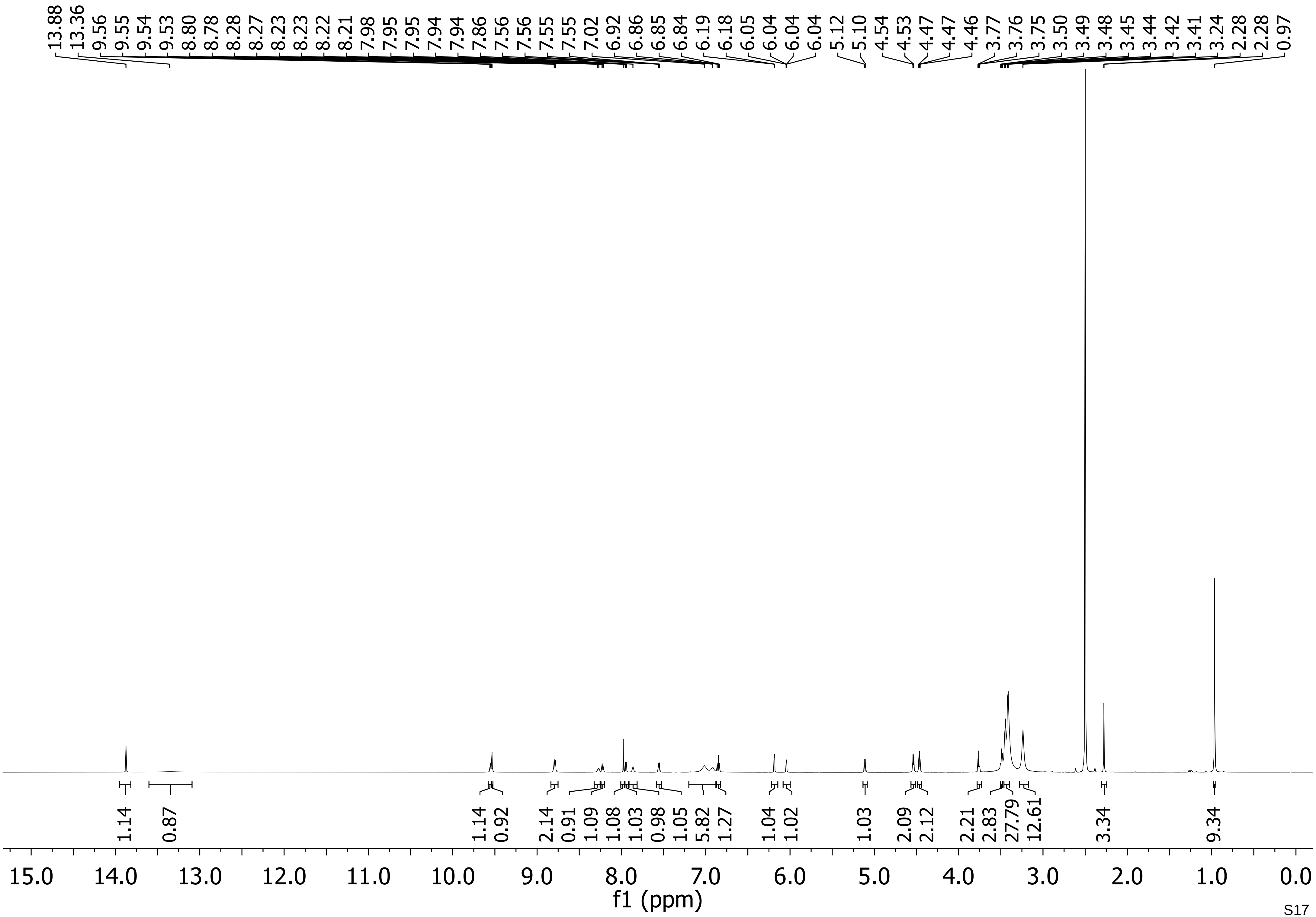

^{1}H NMR (600 MHz, DMSO-d_6) for compound **9a** (zoomed-in view)

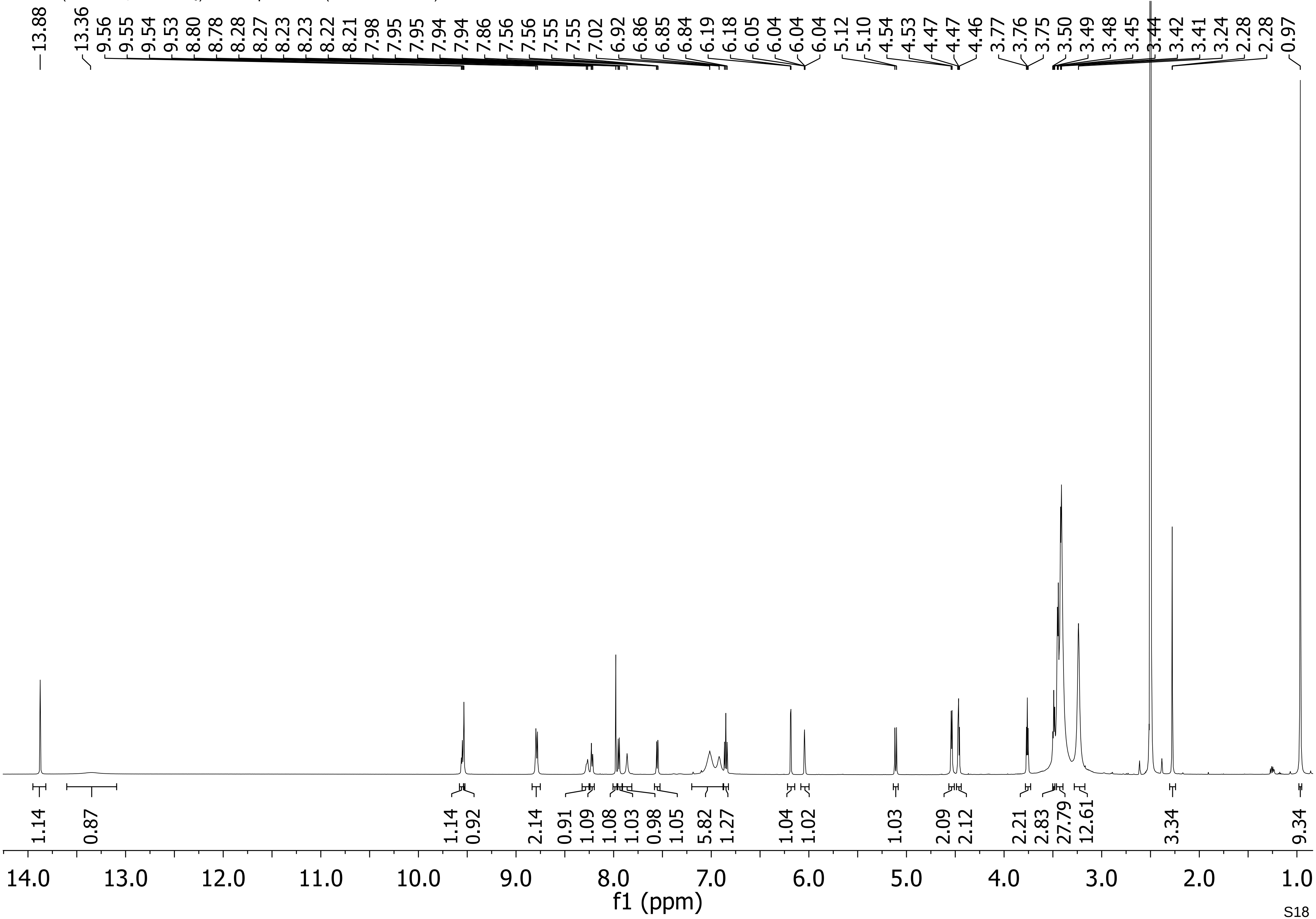

DEPTQ ^{13}C NMR (151 MHz, DMSO-d_6) for compound **9a**

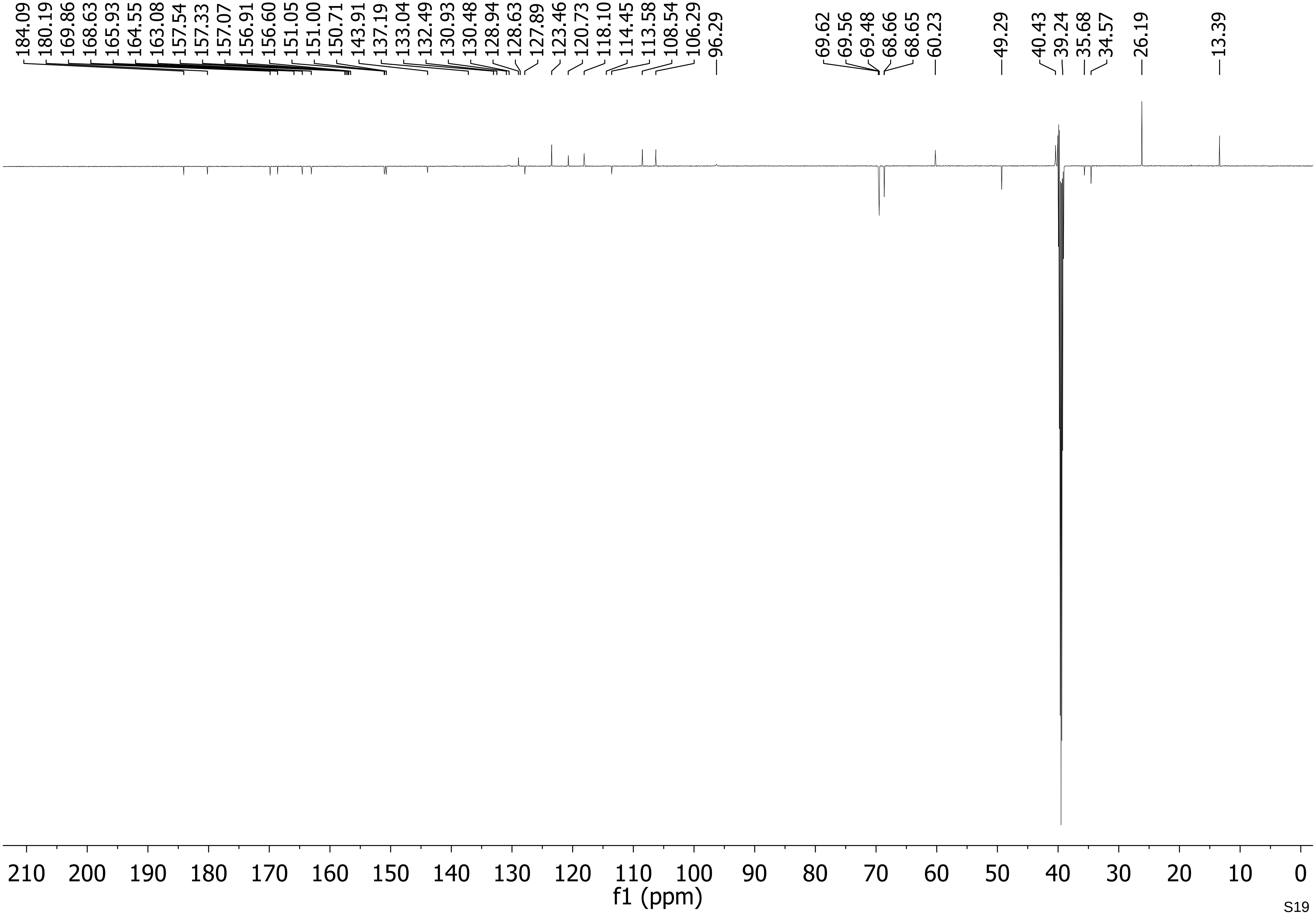

DEPTQ ^{13}C NMR (151 MHz, DMSO-d_6) for compound **9a** (zoomed-in view)

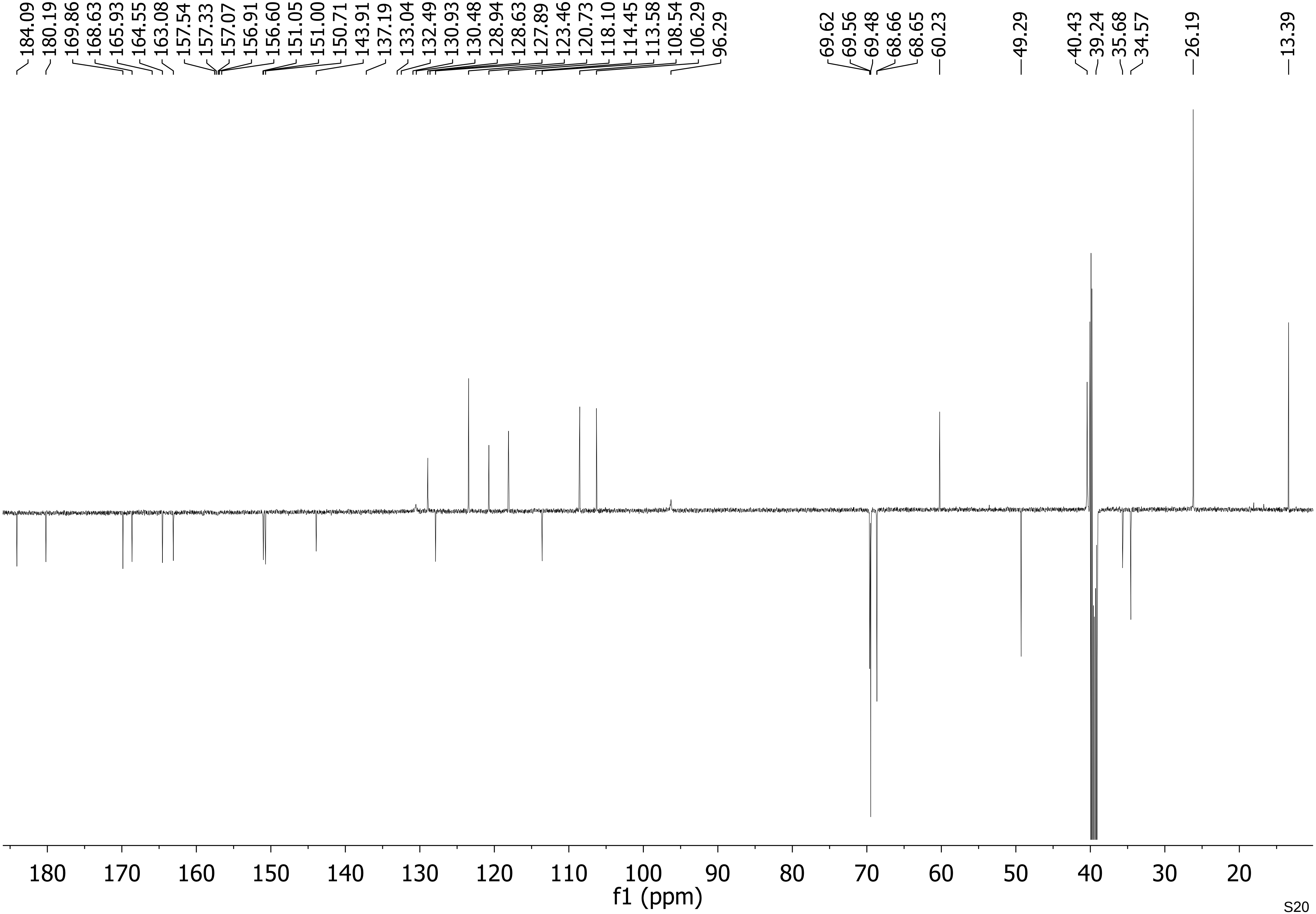

^{1}H NMR (600 MHz, DMSO-d_6) for compound **9b**

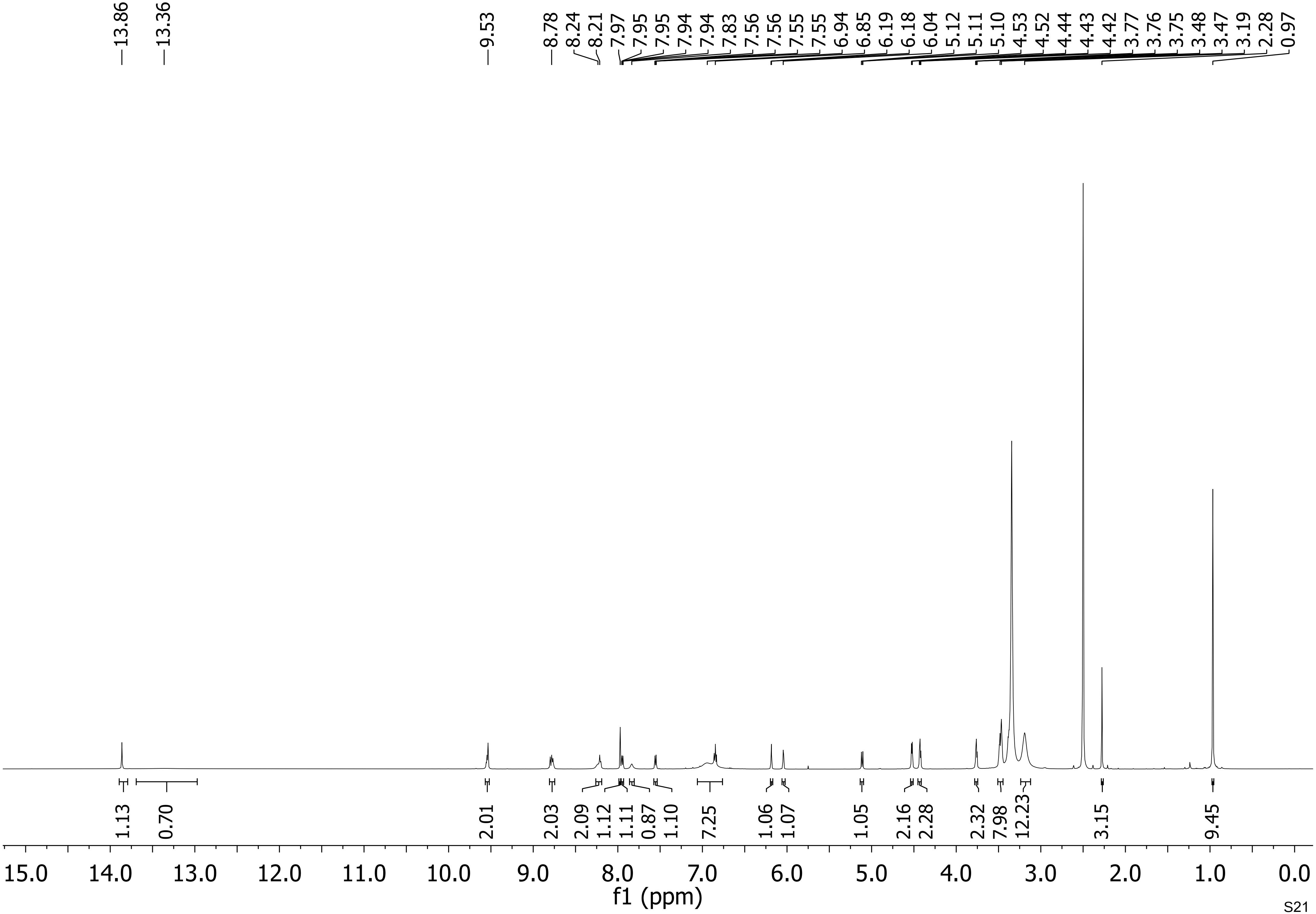

^{1}H NMR (600 MHz, DMSO-d_6) for compound **9b** (zoomed-in view)

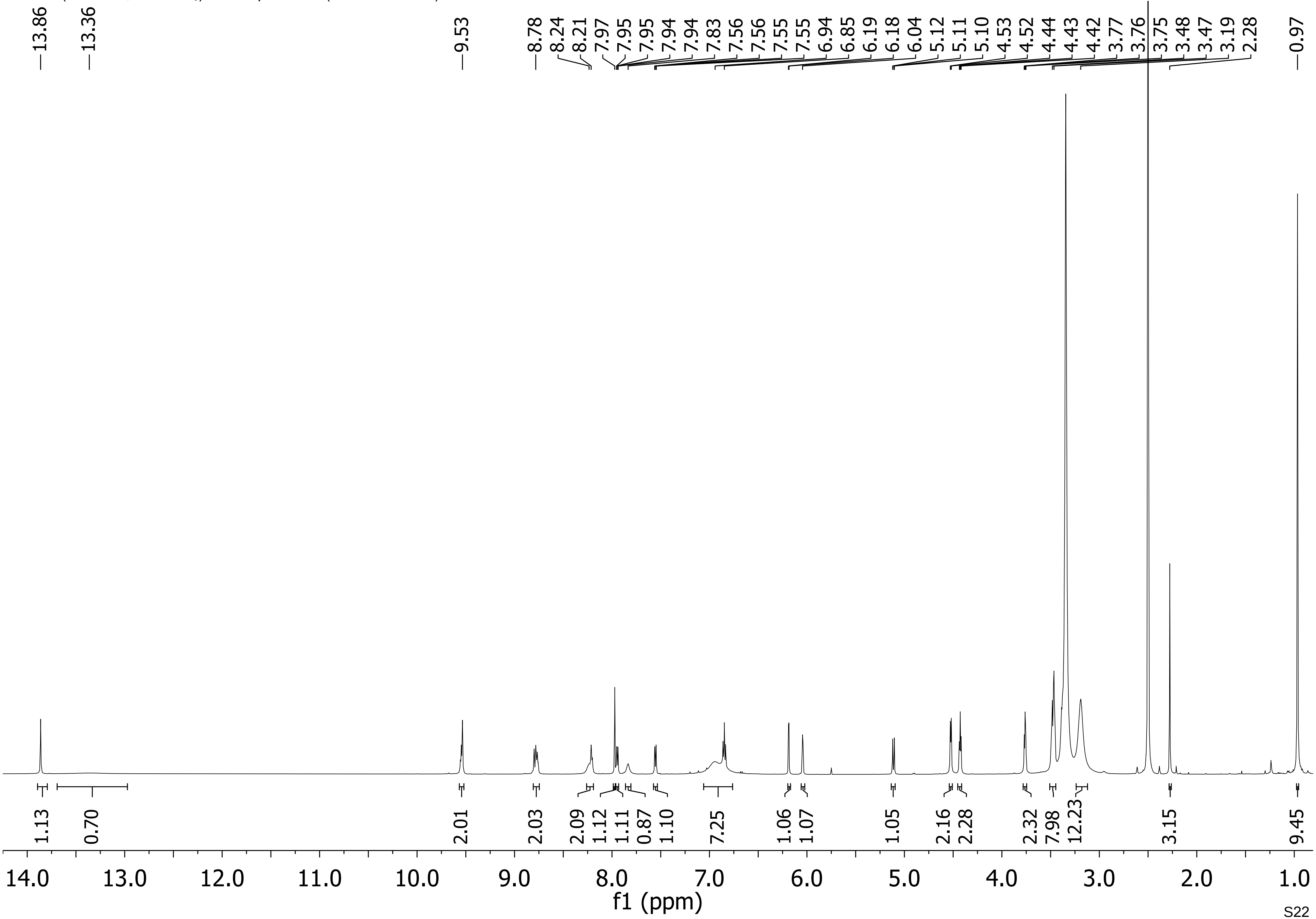

DEPTQ ^{13}C NMR (101 MHz, DMSO-d_6) for compound **9b**

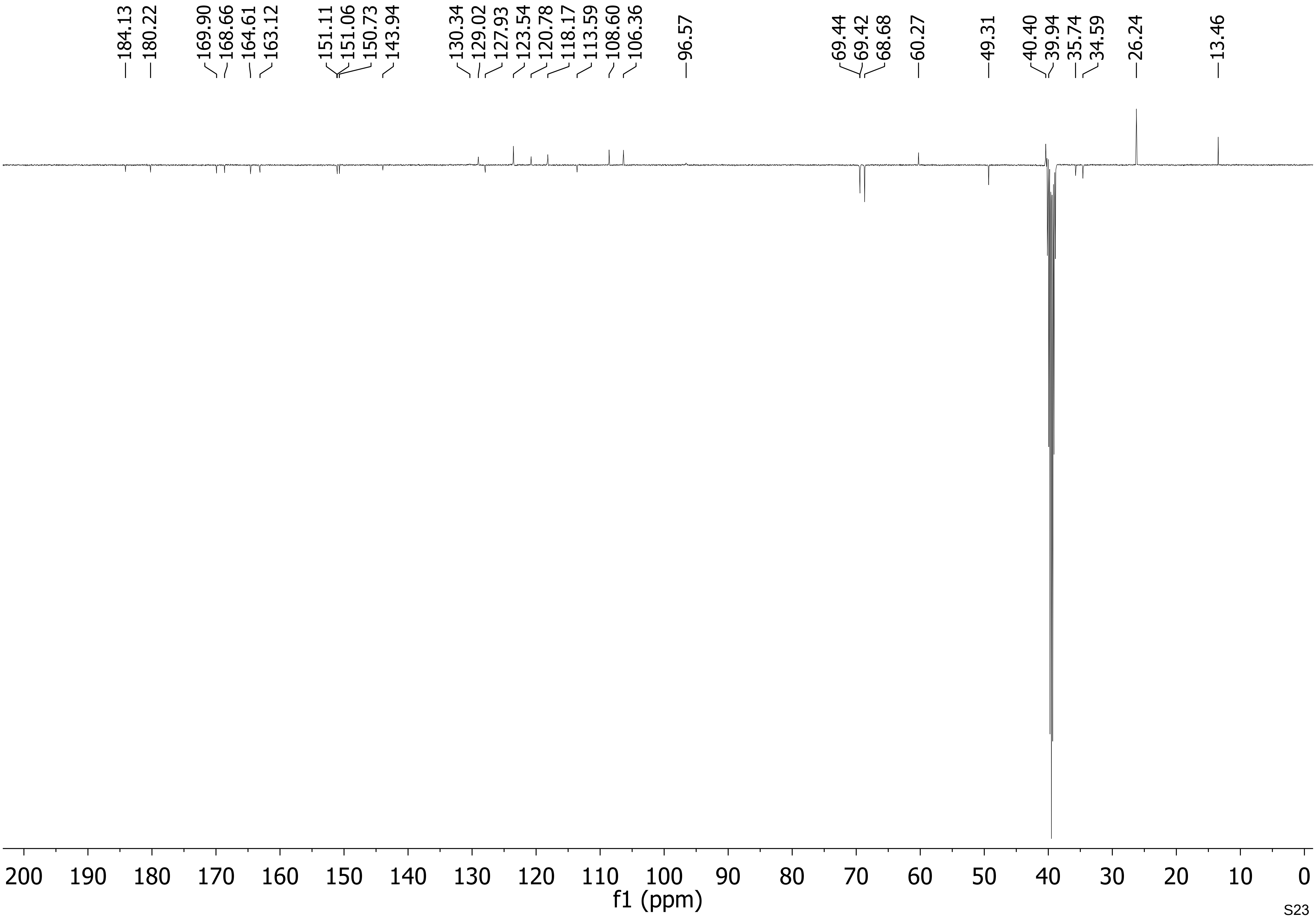

DEPTQ ^{13}C NMR (101 MHz, DMSO-d_6) for compound **9b** (zoomed-in view)

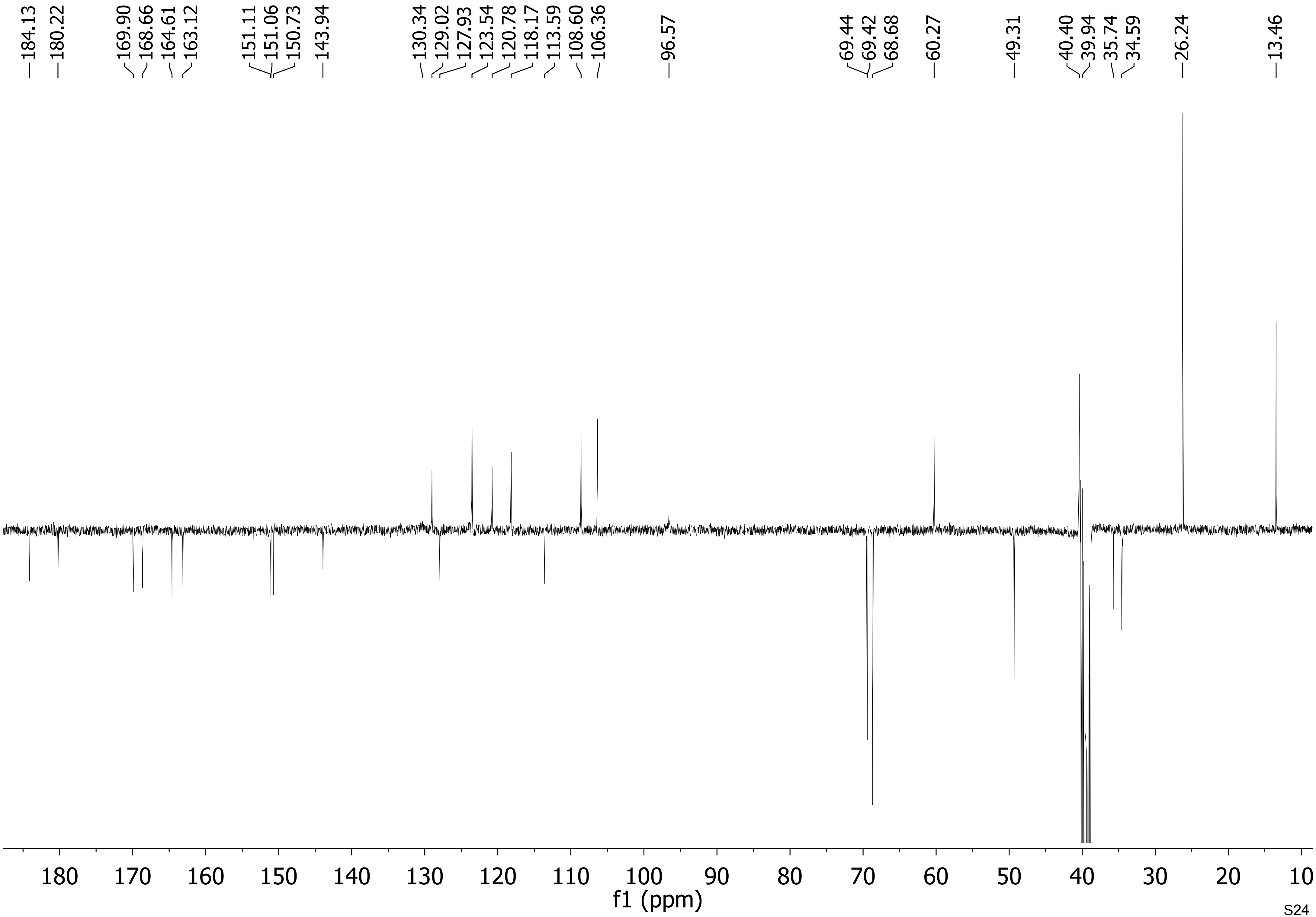

^{1}H NMR (600 MHz, DMSO-d_6) for compound **9c**

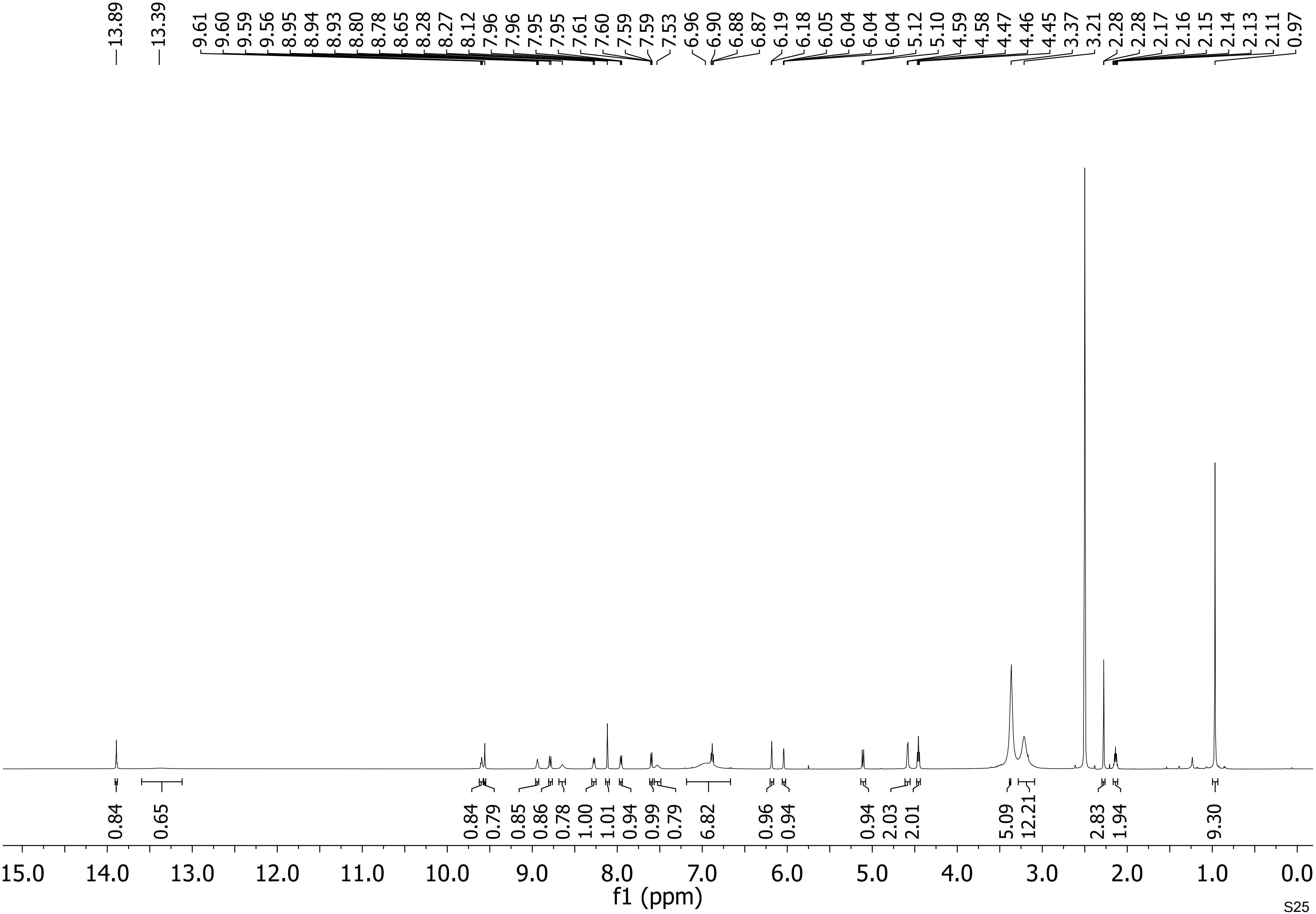

^{1}H NMR (600 MHz, DMSO-d_6) for compound **9c** (zoomed-in view)

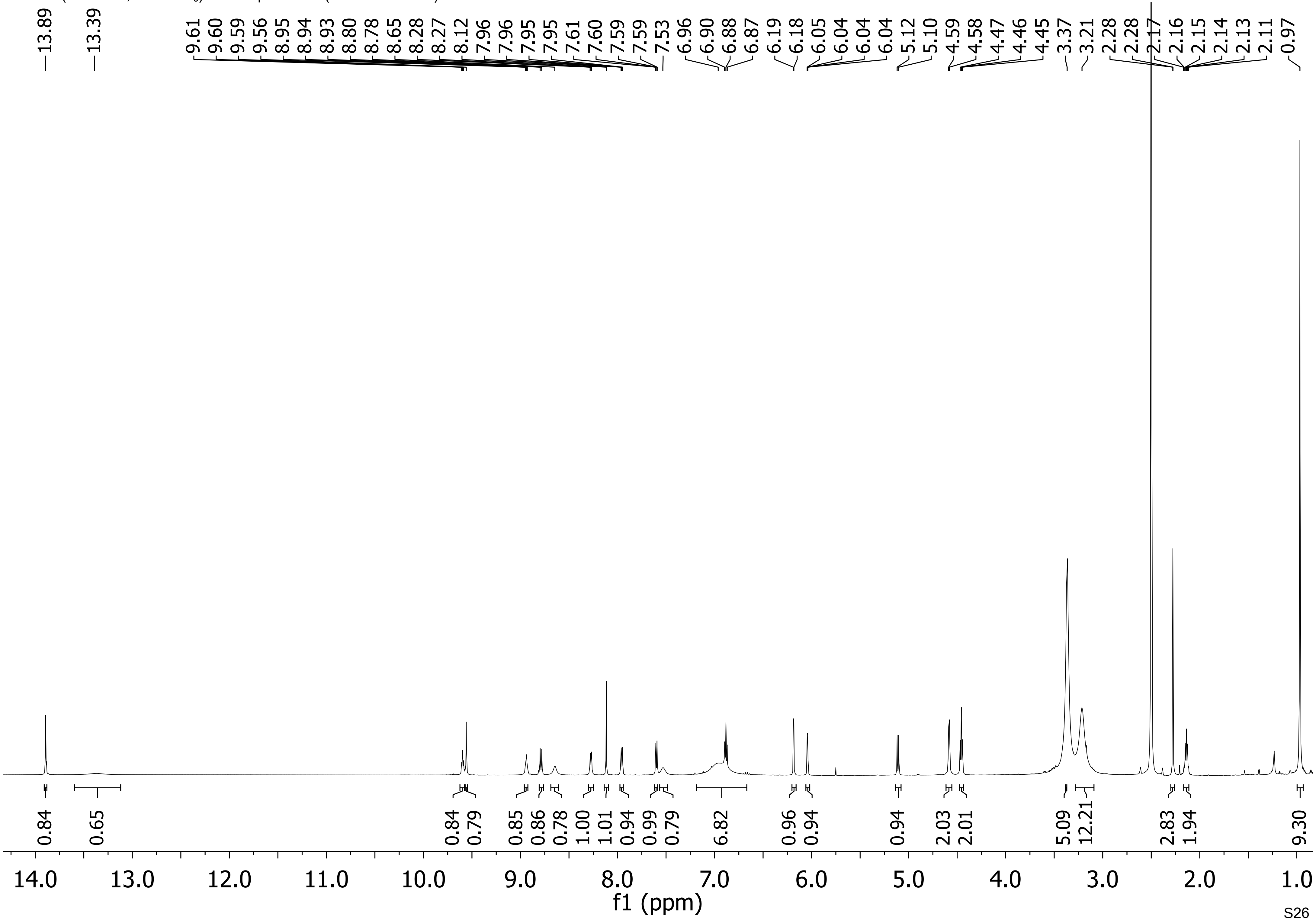

DEPTQ ^{13}C NMR (151 MHz, DMSO-d_6) for compound **9c**

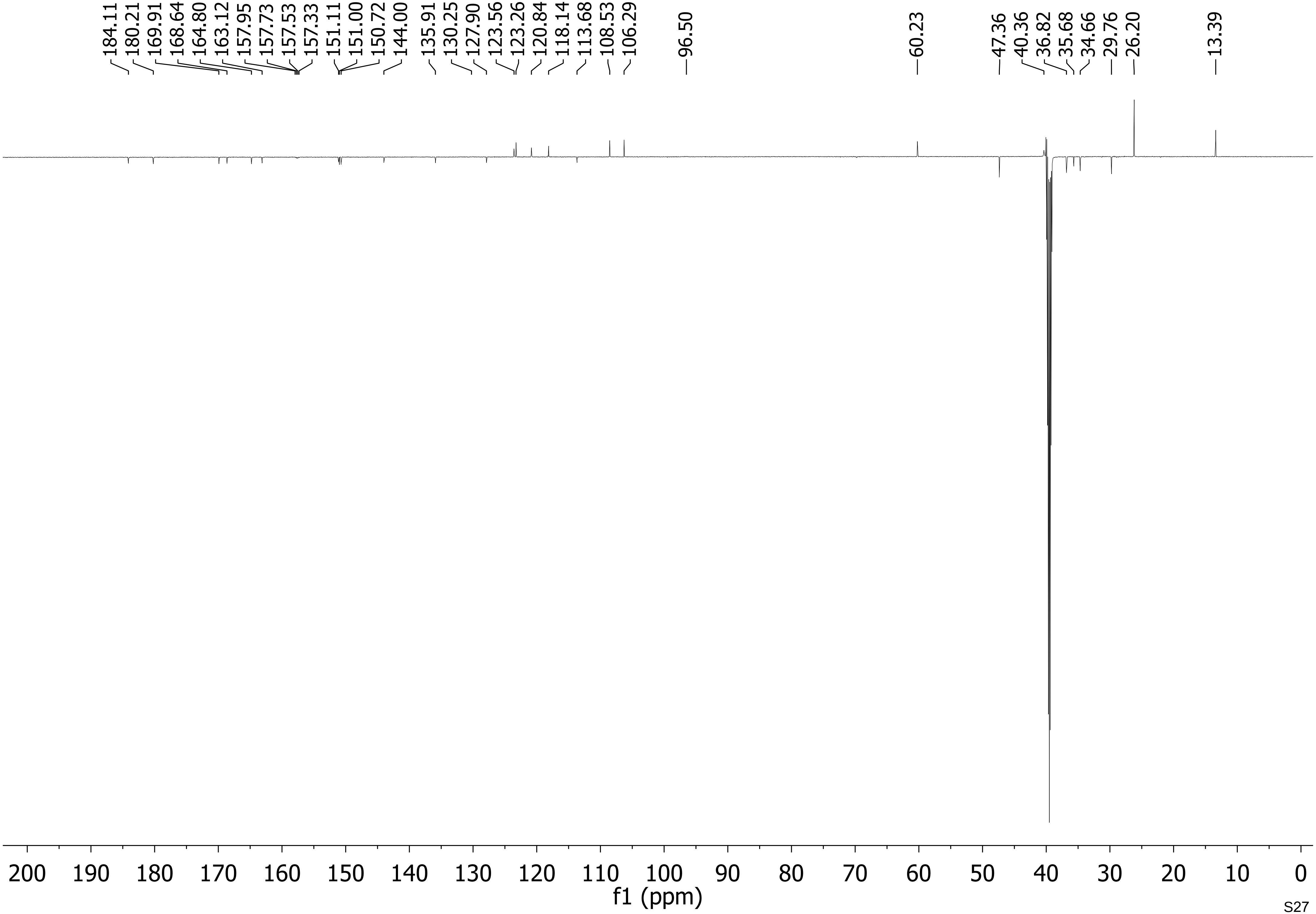

DEPTQ ^{13}C NMR (151 MHz, DMSO-d_6) for compound **9c** (zoomed-in view)

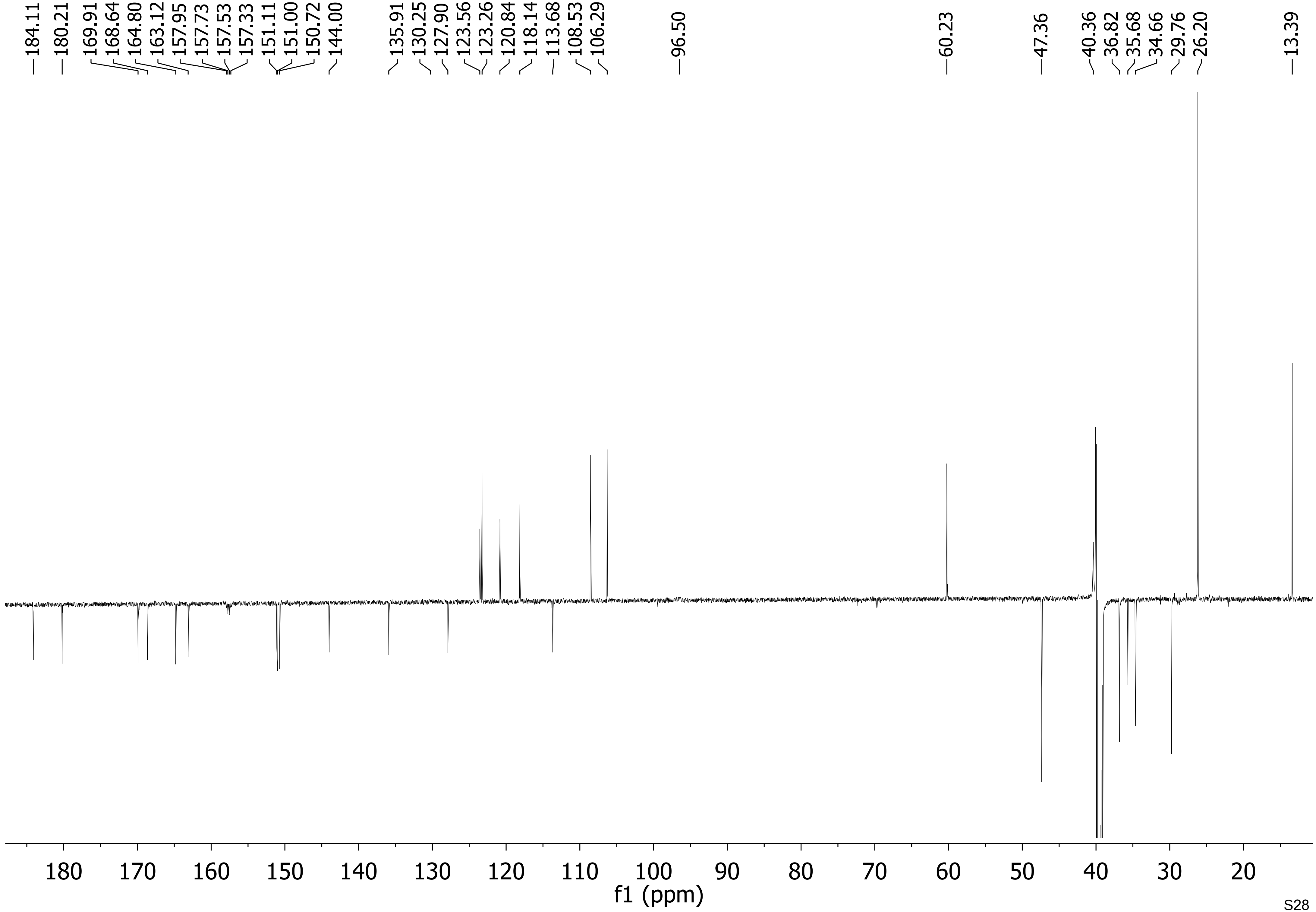

^{1}H NMR (600 MHz, DMSO-d_6) for compound **11**

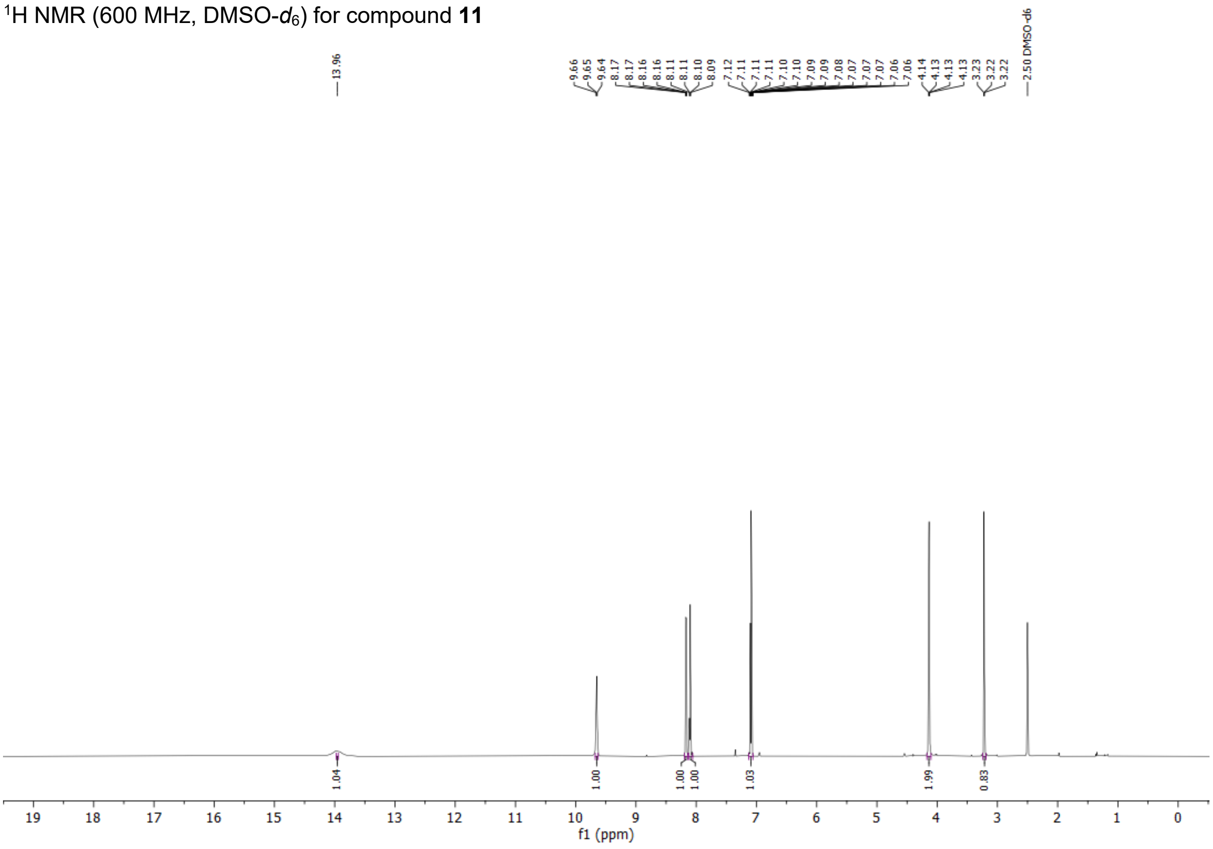

^{13}C NMR (151 MHz, DMSO-d_6) for compound **11**

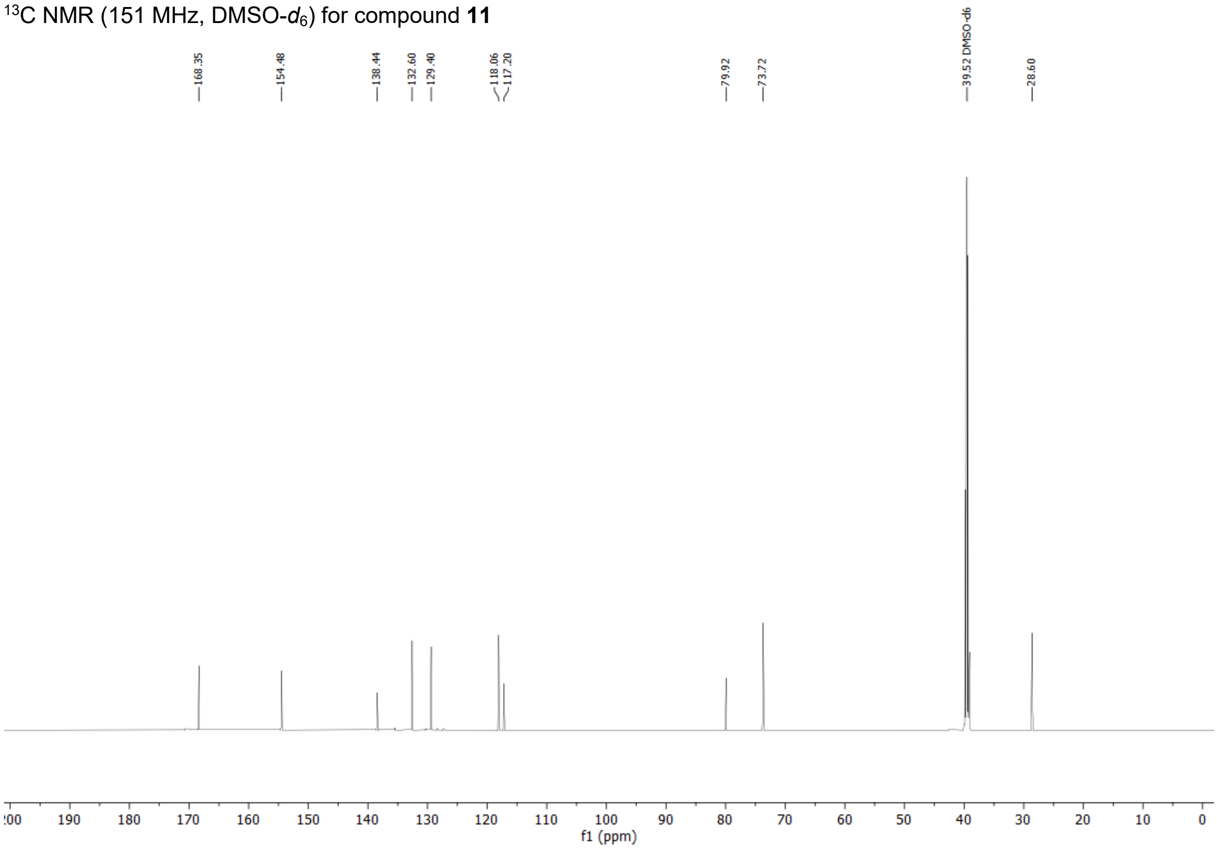

^{1}H NMR (600 MHz, DMSO-d_6) for compound **12**

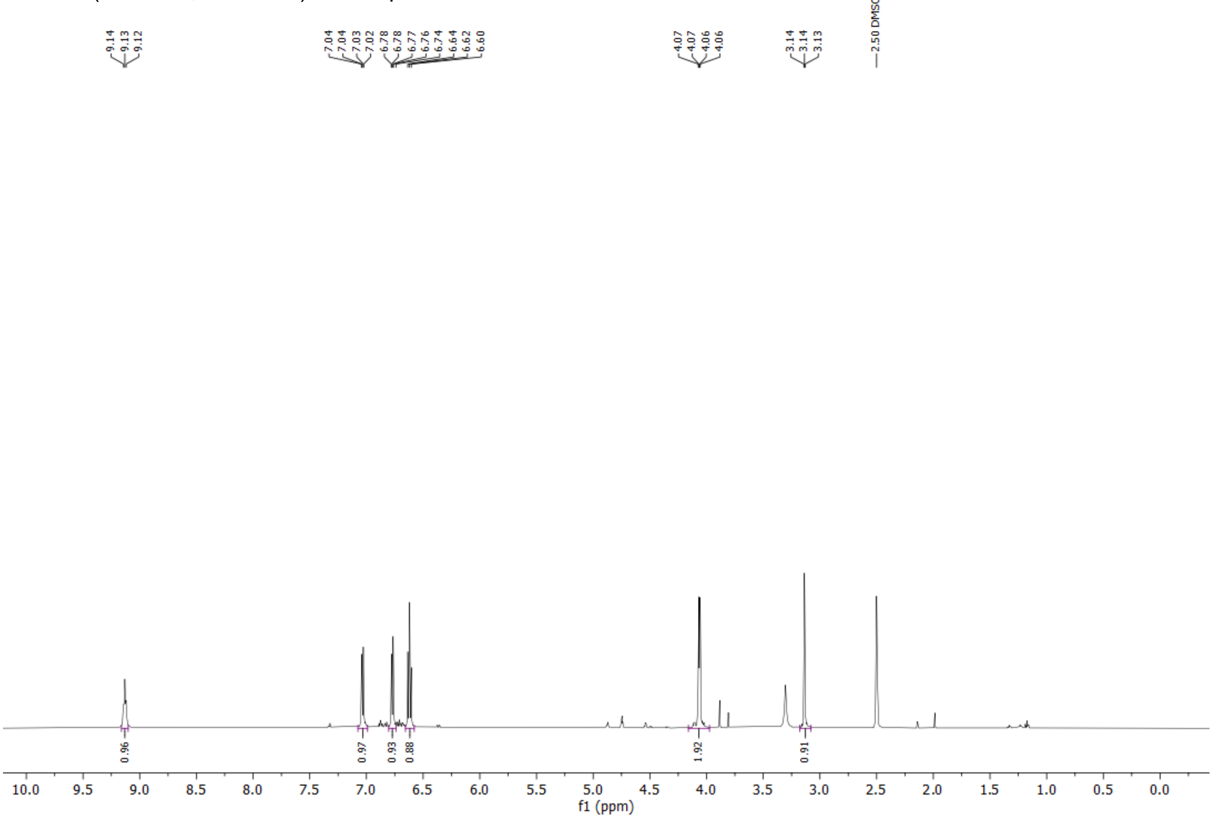

^{13}C NMR (151 MHz, DMSO-d_6) for compound **12**

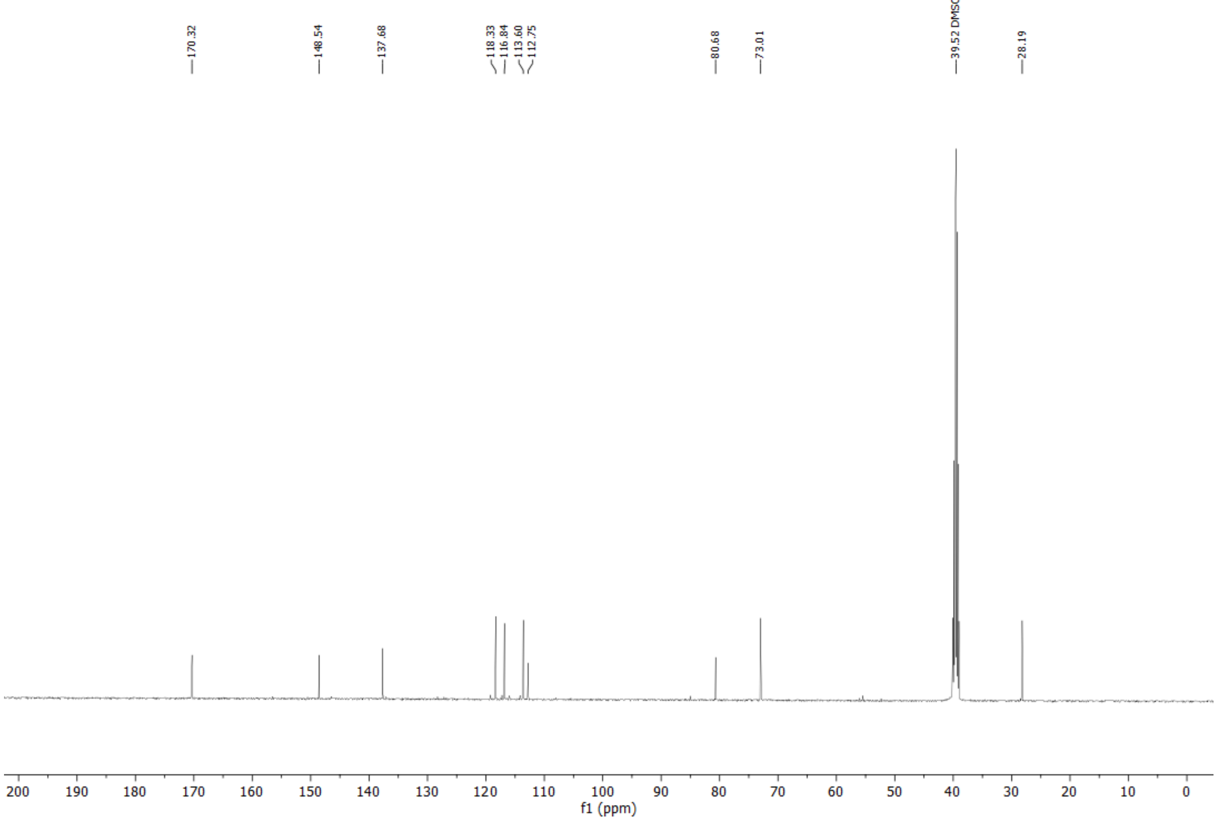

^{1}H NMR (600 MHz, DMSO-d_6) for compound **13**

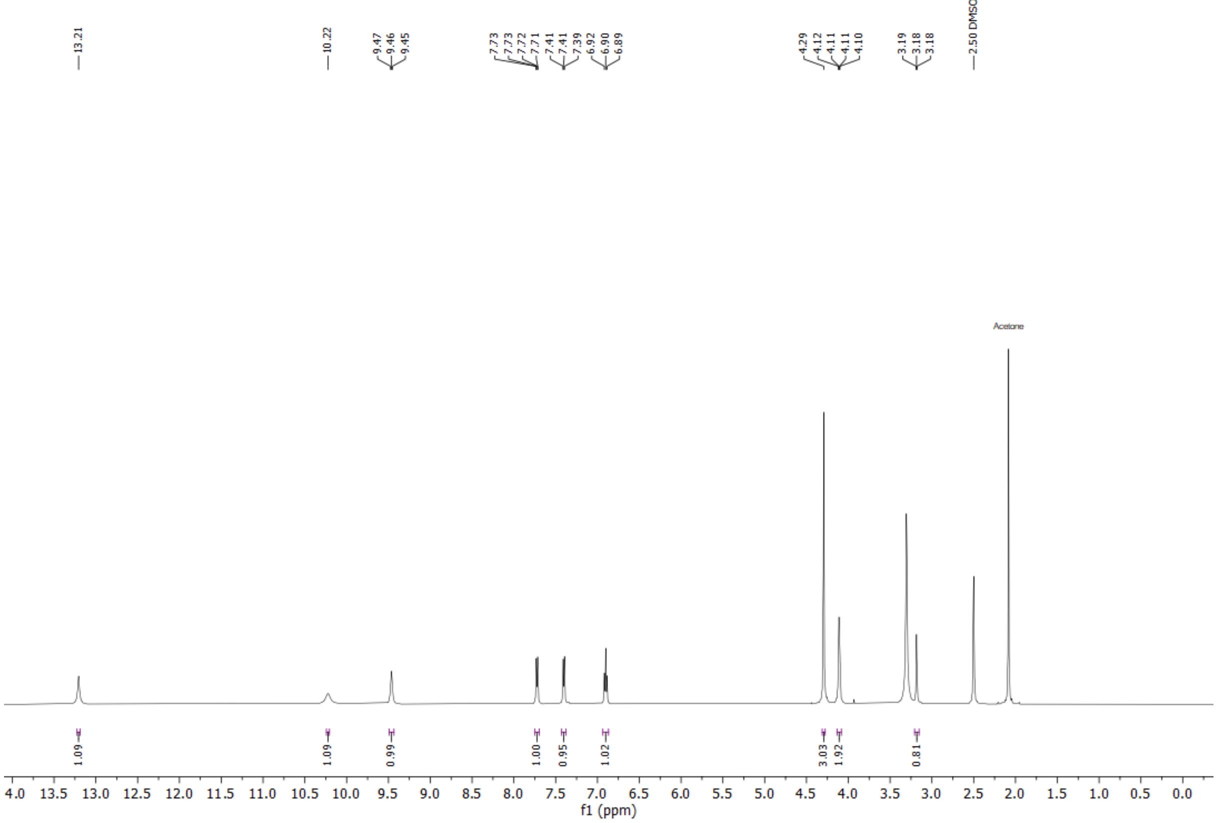

^{13}C NMR (151 MHz, DMSO-d_6) for compound **13**

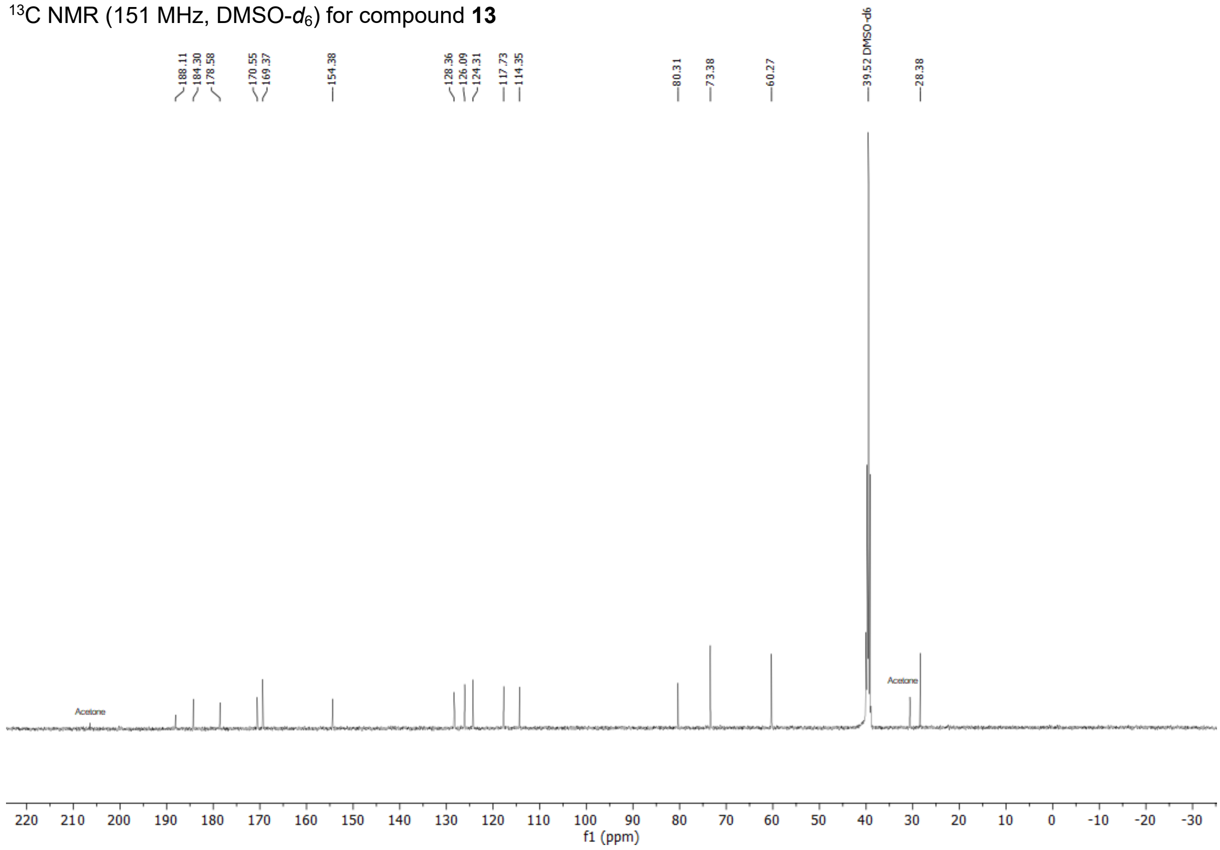

^{1}H NMR (600 MHz, DMSO-d_6) for compound **17**

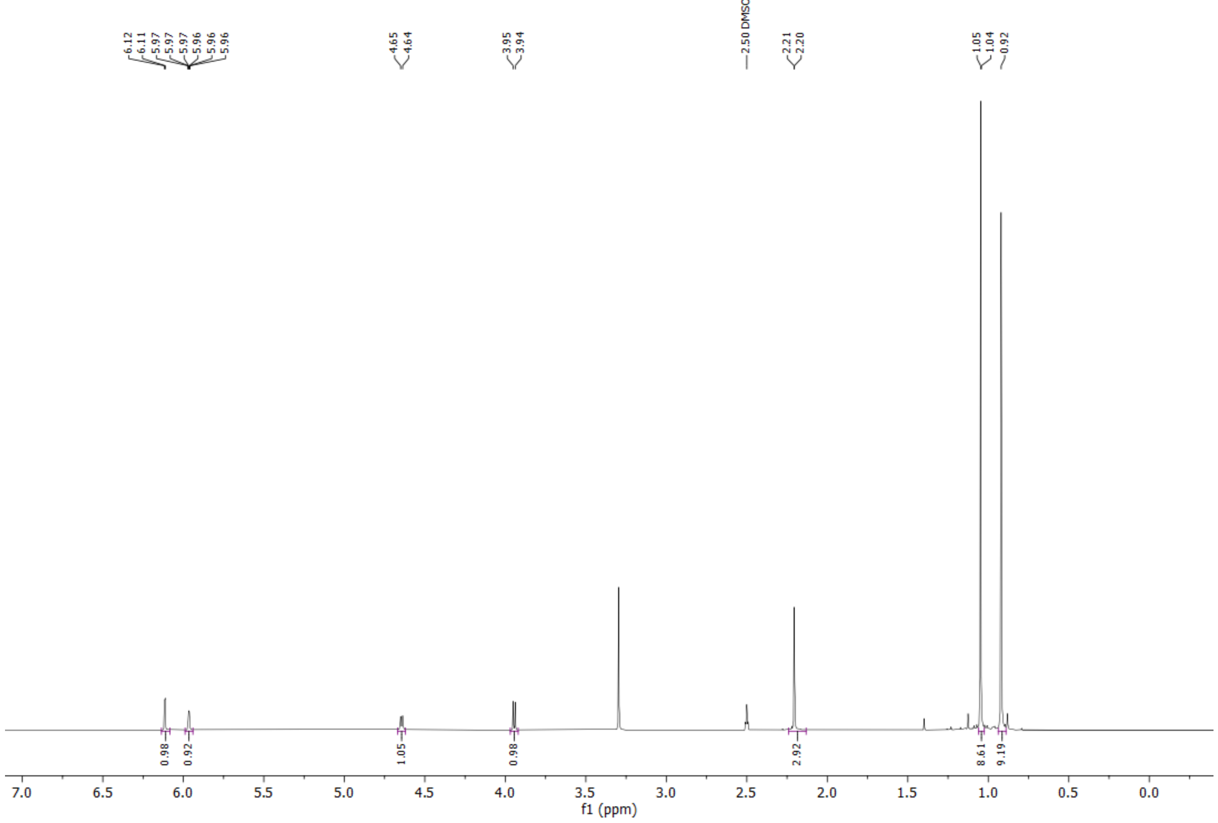

^{13}C NMR (151 MHz, DMSO-d_6) for compound **17**

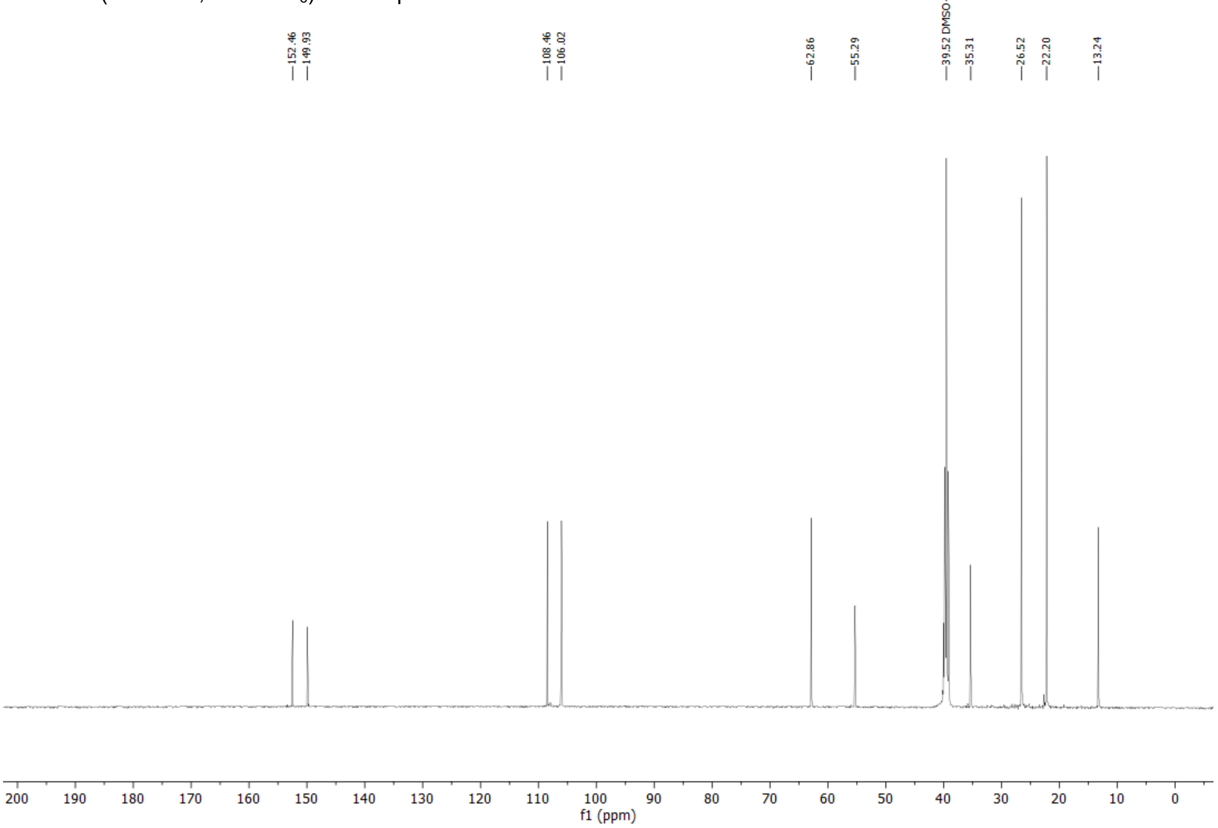

^1H NMR (600 MHz, DMSO-d_6) for compound **18**

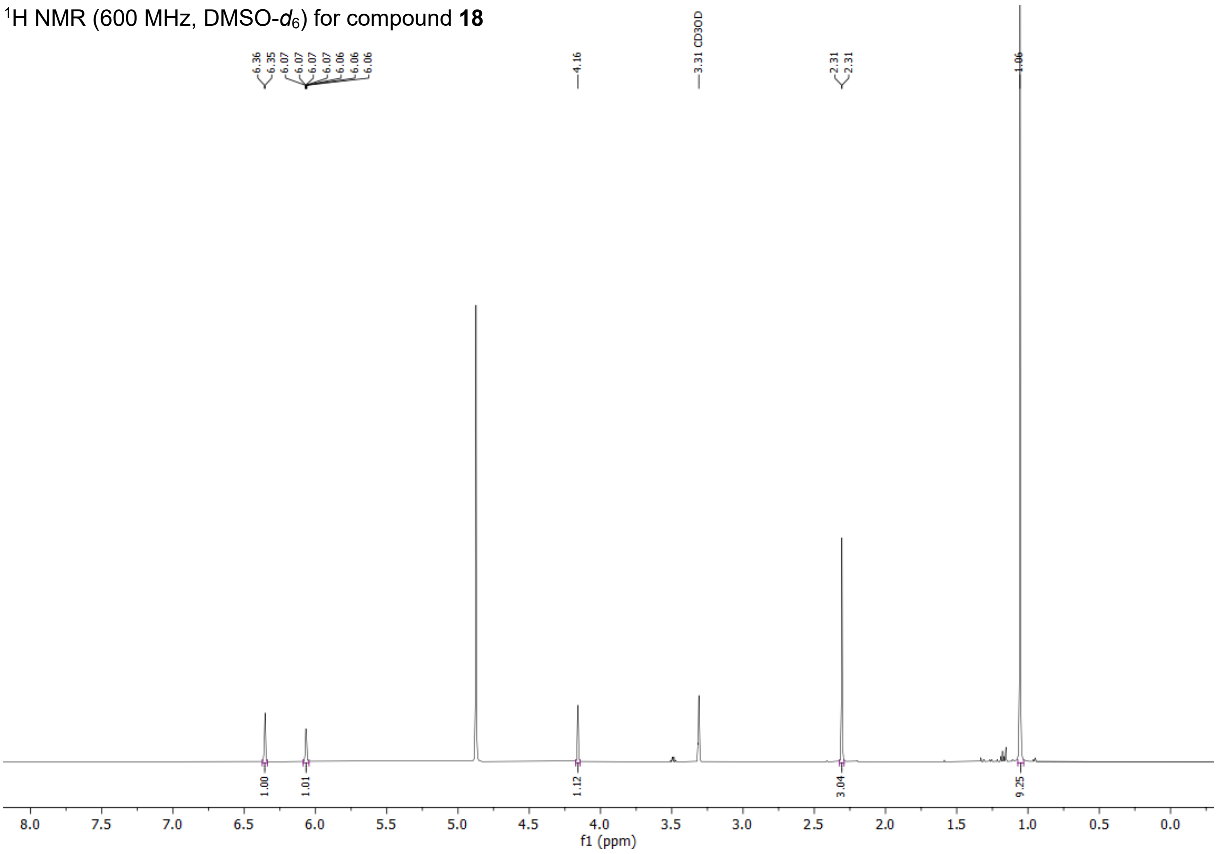

^{13}C NMR (151 MHz, DMSO-d_6) for compound **18**

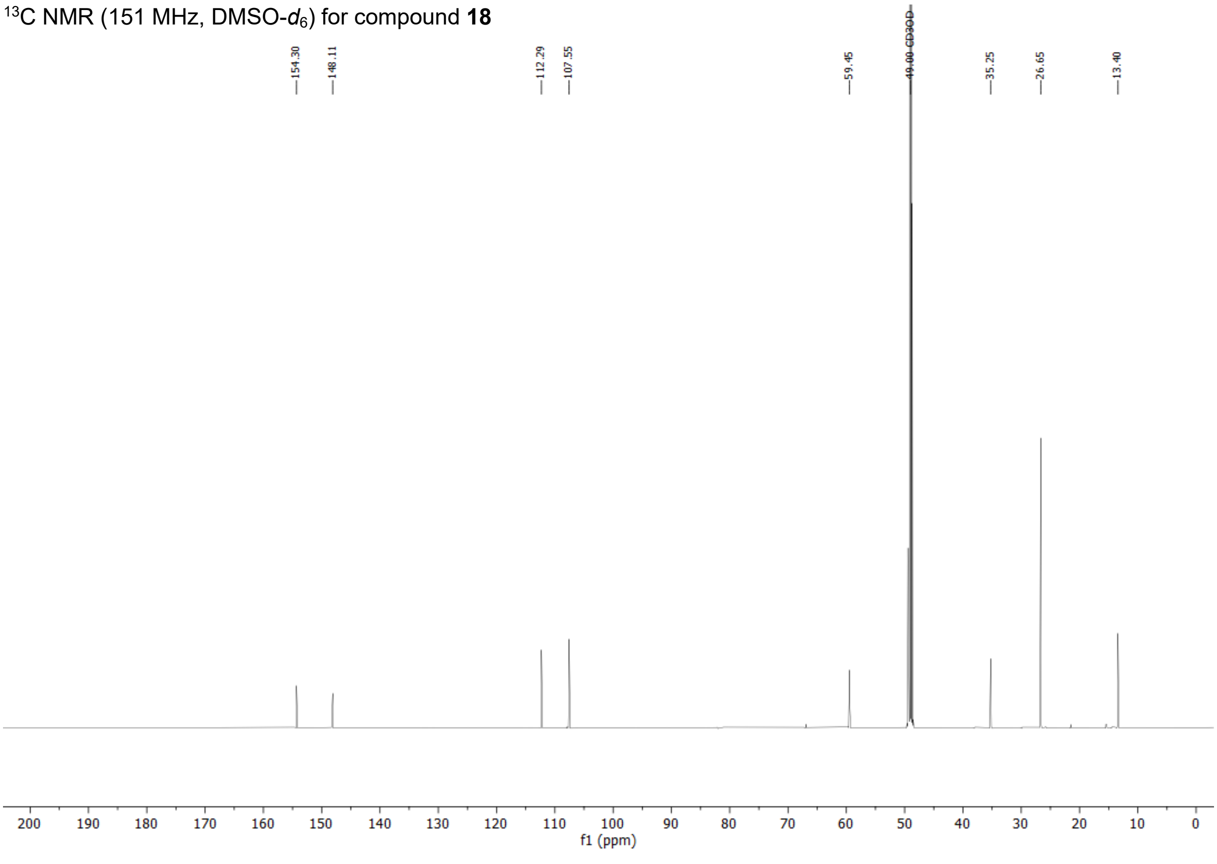

Supplementary HPLC Chromatograms

HPLC chromatogram of **8**:

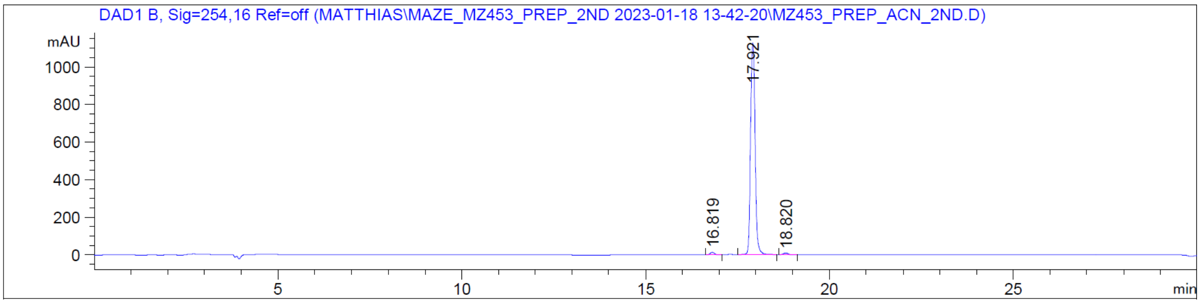


```
=====================================================================
                         Area Percent Report
=====================================================================

Sorted By             :      Signal
Multiplier            :      1.0000
Dilution              :      1.0000
Use Multiplier & Dilution Factor with ISTDs


Signal 2: DAD1 B, Sig=254,16 Ref=off

Peak RetTime Type  Width     Area      Height     Area
  #   [min]        [min]   [mAU*s]     [mAU]       %
----|-------|----|-------|----------|----------|--------|
   1  16.819 BB    0.1385  112.26214   13.02658   1.1315
   2  17.921 VB    0.1370 9718.44336 1122.11121  97.9571
   3  18.820 BB    0.1542   90.42033    9.23734   0.9114

Totals :                  9921.12583 1144.37512
```

HPLC chromatogram of **9a**:

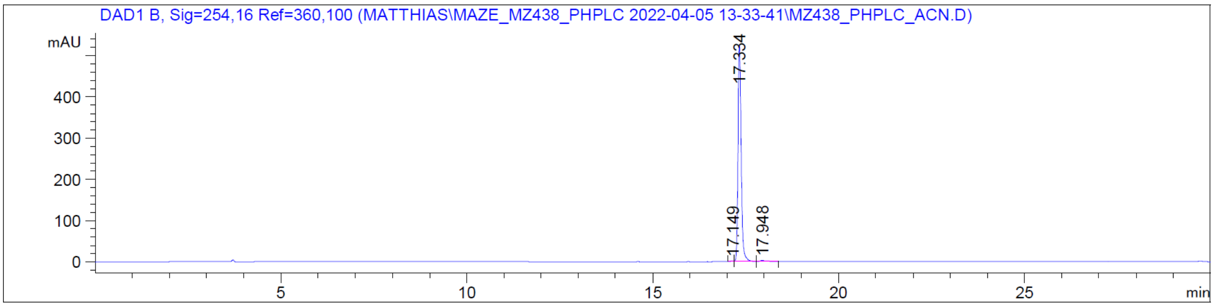


```
=====================================================================
                         Area Percent Report
=====================================================================

Sorted By             :      Signal
Multiplier            :      1.0000
Dilution              :      1.0000
Use Multiplier & Dilution Factor with ISTDs

Signal 2: DAD1 B, Sig=254,16 Ref=360,100

Peak RetTime Type  Width     Area      Height     Area
  #   [min]        [min]   [mAU*s]     [mAU]       %
----|-------|----|-------|----------|----------|--------|
   1  17.149 BV    0.0849   13.15989    2.36613   0.4063
   2  17.334 VB    0.0925 3184.82690  527.34180  98.3266
   3  17.948 BB    0.1834   41.04250    3.04215   1.2671

Totals :                  3239.02929  532.75007
```

HPLC chromatogram of **9b**:

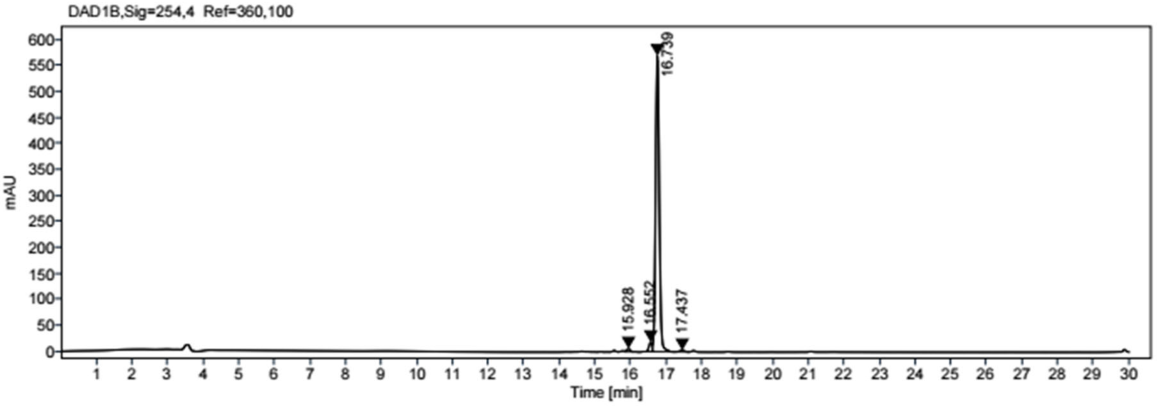

Signal: DAD1B,Sig=254,4 Ref=360,100

RT [min]	Type	Width [min]	Area	Height	Area%
15.928	VB	0.24	41.58	6.08	0.97
16.552	BV	0.19	107.57	18.08	2.52
16.739	VB	0.61	4094.35	568.93	95.83
17.437	BV	0.42	28.83	2.80	0.67
		**Sum**	**4272.33**		

HPLC chromatogram of **9c**:

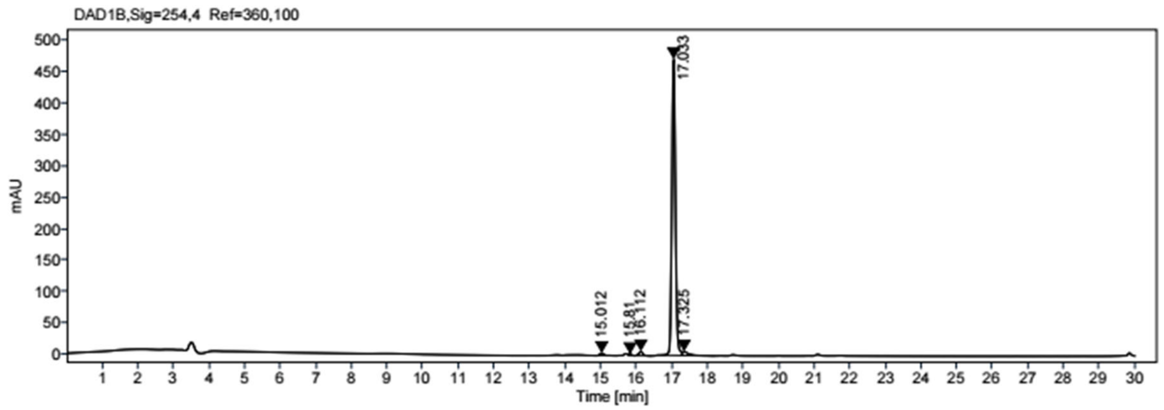

Signal:	DAD1B,Sig=254,4 Ref=360,100				
**RT [min]**	**Type**	**Width [min]**	**Area**	**Height**	**Area%**
15.012	MM m	0.32	26.32	3.68	0.77
15.810	VB	0.16	11.30	1.97	0.33
16.112	BB	0.49	50.60	6.67	1.47
17.033	BV	0.60	3272.32	469.49	95.28
17.325	VB	0.92	73.83	5.82	2.15
		**Sum**	**3434.36**		

HPLC chromatogram of **17** (crude):

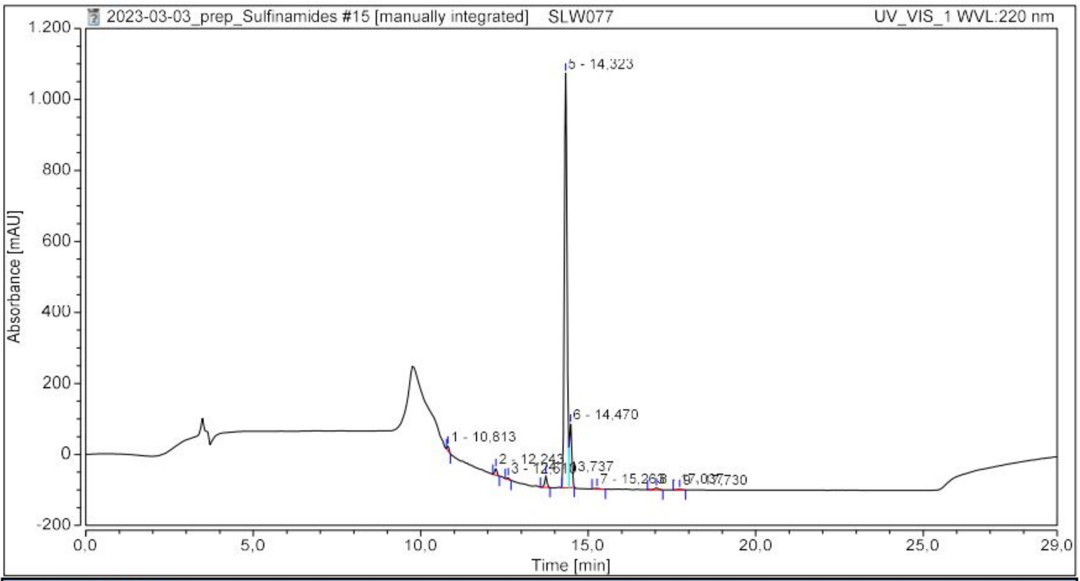

Integration Results							
No.	Peak Name	Retention Time min	Area mAU*min	Height mAU	Relative Area %	Relative Height %	Amount n.a.
1		10,813	0,737	13,283	0,58	0,93	n.a.
2		12,243	1,279	18,596	1,00	1,31	n.a.
3		12,610	0,357	4,797	0,28	0,34	n.a.
4		13,737	2,422	32,763	1,89	2,30	n.a.
5		14,323	107,475	1167,503	83,93	81,96	n.a.
6		14,470	14,563	178,867	11,37	12,56	n.a.
7		15,263	0,303	1,997	0,24	0,14	n.a.
8		17,037	0,679	4,945	0,53	0,35	n.a.
9		17,730	0,239	1,710	0,19	0,12	n.a.
**Total:**			**128,056**	**1424,462**	**100,00**	**100,00**	

HPLC chromatogram of **17** (pure):

Chromatogram

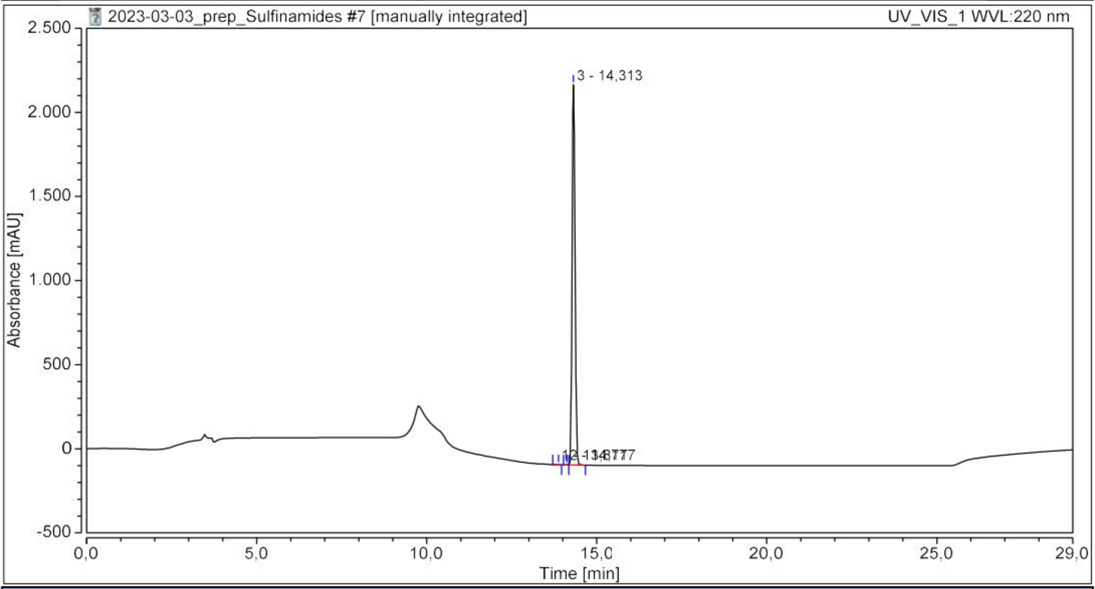

Integration Results							
No.	Peak Name	Retention Time min	Area mAU*min	Height mAU	Relative Area %	Relative Height %	Amount n.a.
1		13,877	0,138	1,504	0,06	0,07	n.a.
2		14,117	0,089	1,299	0,04	0,06	n.a.
3		14,313	227,591	2259,859	99,90	99,88	n.a.
**Total:**			**227,818**	**2262,662**	**100,00**	**100,00**	

Supplementary References

1. Liu, K.; Wu, L.; Yuan, S.; Wu, M.; Xu, Y.; Sun, Q.; Li, S.; Zhao, S.; Hua, T.; Liu, Z. J., Structural basis of CXC chemokine receptor 2 activation and signalling. *Nature* **2020,** *585*, 135-140.
2. Dwyer, M. P.; Yu, Y.; Chao, J.; Aki, C.; Chao, J.; Biju, P.; Girijavallabhan, V.; Rindgen, D.; Bond, R.; Mayer-Ezel, R.; Jakway, J.; Hipkin, R. W.; Fossetta, J.; Gonsiorek, W.; Bian, H.; Fan, X.; Terminelli, C.; Fine, J.; Lundell, D.; Merritt, J. R.; Rokosz, L. L.; Kaiser, B.; Li, G.; Wang, W.; Stauffer, T.; Ozgur, L.; Baldwin, J.; Taveras, A. G., Discovery of 2-hydroxy-N,N-dimethyl-3-{2-[[(R)-1-(5-methylfuran-2-yl)propyl]amino]-3,4-dioxocyclobut-1-enylamino}benzamide (SCH 527123): a potent, orally bioavailable CXCR2/CXCR1 receptor antagonist. *J. Med. Chem.* **2006,** *49*, 7603-7606.
3. Toy, L.; Huber, M. E.; Schmidt, M. F.; Weikert, D.; Schiedel, M., Fluorescent ligands targeting the intracellular allosteric binding site of the chemokine receptor CCR2. *ACS Chem. Biol.* **2022,** *17*, 2142-2152.
4. Huber, M. E.; Toy, L.; Schmidt, M. F.; Vogt, H.; Budzinski, J.; Wiefhoff, M. F. J.; Merten, N.; Kostenis, E.; Weikert, D.; Schiedel, M., A chemical biology toolbox targeting the intracellular binding site of CCR9: fluorescent ligands, new drug leads and PROTACs. *Angew. Chem. Int. Ed.* **2022,** *61*, e202116782.
